# Major alleles of *CDCA7* shape CG methylation in *Arabidopsis thaliana*

**DOI:** 10.1038/s41477-025-02148-w

**Published:** 2025-11-07

**Authors:** Pierre Bourguet, Zdravko J. Lorković, Darya Kripkiy Casado, Valentin Bapteste, Chung Hyun Cho, Anna A. Igolkina, Cheng-Ruei Lee, Magnus Nordborg, Frédéric Berger, Eriko Sasaki

**Affiliations:** 1 Gregor Mendel Institute of Molecular Plant Biology, Austrian Academy of Sciences, Vienna BioCenter, Vienna, Austria; 2 Institute of Ecology and Evolutionary Biology, National Taiwan University, Taipei, Taiwan; 3 Faculty of Science, Kyushu University, Fukuoka, Japan

**Keywords:** Plant genetics, Population genetics, Epigenetics

## Abstract

DNA methylation is a key epigenetic mark that impacts gene expression and represses transposable elements in eukaryotes. Numerous examples of *cis* elements targeted by DNA methylation, particularly at CG sites (mCG), have been reported to be under selective pressure in animals and plants. By contrast, there is limited knowledge of *trans* regulators of mCG leading to adaptation. Here, a genome-wide association study identifies CELL DIVISION CYCLE-ASSOCIATED PROTEIN 7 (CDCA7) as a major *trans* determinant of mCG in natural populations of *Arabidopsis thaliana*. CDCA7 or its paralogue physically binds the chromatin remodeller DECREASE IN DNA METHYLATION 1 (DDM1), which facilitates access of methyltransferases to DNA. Epigenomic analysis shows that while CDCA7 proteins control all DDM1-dependent processes, their predominant function is the maintenance of mCG. We identify a 26-bp promoter indel modulating *CDCA7* expression in natural populations and determining the degree of mCG and transposable element silencing. The geographic distribution of *CDCA7* alleles suggests that new alleles have repeatedly expanded to novel ecological niches, indicating a potential role in local adaptation. Our findings establish CDCA7 proteins as dedicated regulators of mCG and imply that DDM1 relies on alternative partners to regulate other chromatin features. Broadly, they illustrate how changes in global DNA methylation levels through transcriptional regulation of the epigenetic machinery have the capacity to facilitate local adaptation.

## Abstract (web summary)

This genome-wide association study identifies CELL DIVISION CYCLE-ASSOCIATED PROTEIN 7 (CDCA7) as a regulator of DNA methylation in natural *Arabidopsis thaliana* populations. CDCA7 binds the chromatin remodeller DDM1 and modulates the control of CG methylation.

## Main

5-Methylcytosine (5mC) is the predominant form of DNA modification in multicellular eukaryotes^1,2^. 5mC regulates gene expression during development and preserves genome integrity by repressing transposable elements (TEs)^3,4^. While conserved molecular pathways control the genomic distribution of 5mC^5,6^, the epigenome varies between individuals due to genetic differences, environmental influences and stochastic catalytic activity^7–12^. In many organisms, epigenome variation contributes to phenotypic variation at the cellular, individual and population levels^5,13–18^. Key remaining questions are how this epigenome variation was established and whether it contributes to adaptive evolution in nature.

Proteins essential for 5mC maintenance are well characterized in plants^19^. Methylation of CG dinucleotides (mCG) is maintained by METHYLTRANSFERASE 1 (MET1), while non-CG methylation (mCH, where H is A, T or C) requires the chromomethyltransferases (CMT) CMT2 and CMT3 and the RNA-directed DNA methylation (RdDM) pathway. MET1 and CMT2/3 rely on the chromatin remodeller DECREASE IN DNA METHYLATION 1 (DDM1), which facilitates methyltransferase access to DNA by remodelling nucleosomes.

In wild plant populations, 5mC levels are often associated with local climates^20–24^. The 1001 Epigenomes Project revealed 5mC variation for 1,107 worldwide accessions of *Arabidopsis thaliana*, and genome-wide association studies (GWAS) demonstrated that at least some of it had a genetic basis^23^. mCH variation is controlled by genetic variation at *trans*-acting modifiers, including *CMT2*, *CMT3* and *NRPE1*, a subunit of RNA polymerase V^23,25,26^. These regulators influence TE activity, and their allelic distribution shapes geographic clines^25,27^. *Trans* regulators of mCG variation in gene bodies have been studied^9,28,29^, although the function of gene-body methylation remains debated^30^. In contrast, the genetic basis of mCG variation at TEs remains unknown, despite its critical role in transcriptional silencing and genome stability.

Here we investigated *trans* regulators of mCG variation at TEs in natural *A. thaliana* lines. Using GWAS, we identified *CDCA7α*, an orthologue of the human *Cell division cycle-associated protein 7* (ref. ^31^), as a major *trans* regulator of mCG variation. We show that *A. thaliana* CDCA7α and its paralogue, CDCA7β, act as facultative activators of DDM1 and specifically promote its function in depositing mCG. In natural populations, the highly diverged *CDCA7α* promoter region modulates its expression, thus influencing the degree of mCG and TE silencing genome-wide. New *CDCA7α* alleles emerged multiple times after speciation and accompanied expansion to new ecological niches, suggesting a role of mCG regulation in local adaptation.

## Results

### Natural TE mCG is controlled by genetic variants of *CDCA7α*

**Fig. 1:**
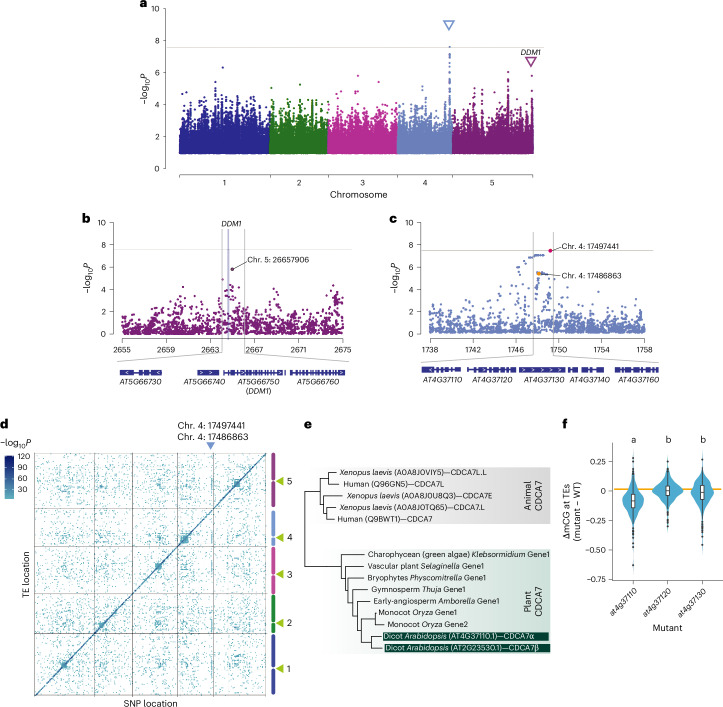
The genetic architecture of natural mCG variation at TEs. **a**, Manhattan plot of the GWAS for mCG on CMT2-targeted TEs. The GWAS in **a**–**c** were conducted using a linear mixed model with correction of population structure. The horizontal lines in **a**–**c** show the Bonferroni-corrected significance threshold (*P* = 0.05). **b**,**c**, Zoomed-in figures showing the *DDM1* region (**b**) and the highest peak (**c**) with genes in these regions. The purple verticle line in **b** shows the location of *DDM1*. **d**, Genetic association for mCG on individual TEs. Associations with −log_10_*P* > 6 are shown. The green triangles indicate centromeric regions. **e**, Simplified phylogenetic tree of CDCA7 in vertebrates and plants. The full tree is shown in Extended Data Fig. 2. **f**, mCG levels of 229 TEs associated with chromosome 4 position 17486863 for loss-of-function mutants, normalized to the WT. The combined violin and box plots show the data distribution. The orange line shows no difference between the WT and mutants. Groups not sharing the same letter are significantly different (two-sided Tukey’s honestly significant difference (HSD) test, *P* < 1 × 10^−7^). This experiment was not replicated, but the effect was confirmed in subsequent experiments (Fig. 2b). The box plots show the median (centre line), the interquartile range (IQR; 25th–75th percentiles; box limits), the minimum and maximum values within 1.5× IQR (whiskers) and outliers (points beyond the whiskers). Source data

**Extended Data Fig. 1:**
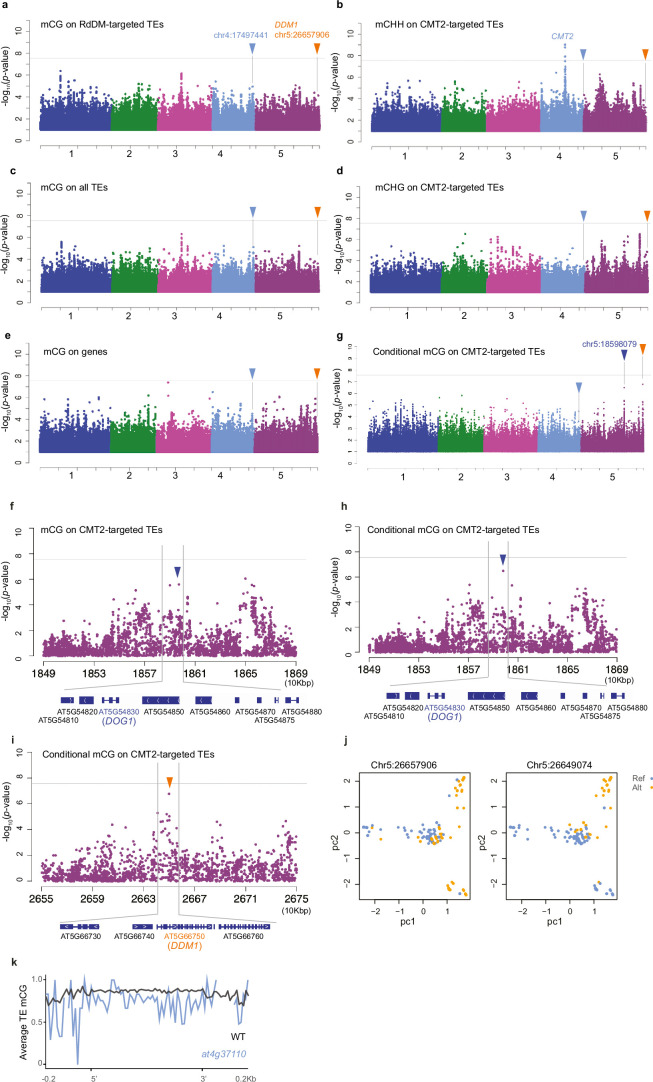
The genetic basis of various DNA methylation contexts in natural populations. GWAS for mCG on RdDM-targeted TEs (**a**), all TEs (**c**), genes (**e**), and CMT2-targeted TEs, conditioned on the genotype of chr4:1749744 (**g**). GWAS for mCHH (**b**) and mCHG (**d**) on CMT2-targeted TEs. Zoom-in figures compare a GWAS peak for mCG on CMT2-targeted TEs around chr5:18598079 (**f**) with the conditioned peak based on the genotype at chr4:1749744 (**h**). The conditioned peak at chr5:26657906 is shown in (**i**). Arrows on the Manhattan plots indicate SNP positions at chr4:17497441 (light blue), chr5:1859079 near *DOG1* (blue), and chr5:26657906 near *DDM1* (orange), respectively. GWAS in **a-i** were conducted using a linear mixed model with correction of population structure. The horizontal lines show the threshold (*p*-value = 0.05) after conducting multiple testing correction using Bonferroni. **b** and **d** are replotted from a previous study^23^, and **e** is replotted from^25^. **j**, local genetic structure of *DDM1* locus by PCA. Lines carrying reference and alternative alleles are mapped for chr5:26657906 (left) and chr:26649074 (right). **k**, Metaplot showing mCG in TEs associated with chr4:17486863 in WT and *at4g37110* mutant.

To explore the genetic architecture of mCG variation, we conducted GWAS using the 1001 Epigenomes dataset (*n* = 774)^23^. We focused on mCG levels from two domains of chromatin in which TEs are repressed by distinct mechanisms: euchromatic regions, represented by RdDM-targeted TEs, and heterochromatic regions, represented by CMT2-targeted TEs^23^ (Methods) (Fig. 1a and Extended Data Fig. 1a–e).

While no genetic variant was associated with average mCG in RdDM-targeted TEs, several peaks were detected in GWAS for average mCG levels in CMT2-targeted TEs on chromosomes 4 and 5 (Fig. 1a, Extended Data Fig. 1a–f and Supplementary Table 1). The association on the right end of chromosome 5 (position 26657906) was not statistically significant but was located just 4.8 kb downstream of the coding region of *DDM1* and covered the coding to the 3′ region of *DDM1* (−log_10_*P* = 5.80; minor allele frequency (MAF), 47%) (Fig. 1b and Extended Data Fig. 1j). This peak may reflect several causative alleles, and one allele is associated with a single nucleotide polymorphism (SNP) at chromosome 5 position 26649074 in the first exon of *DDM1* resulting in an amino acid substitution (V9F). This amino acid is not conserved across DDM1 orthologues in angiosperms, does not belong to a domain of known function (Supplementary Fig. 1a,b) and is not represented in available cryo-electron microscopy structures^32–35^. Given the known major role of *DDM1* in the control of mCG at CMT2-dependent TEs^36,37^, a peak in *DDM1* is not unexpected, and the association is unlikely to be spurious.

The significant peak on chromosome 4 spanned a 15-kb region from positions 17482000 to 17497000 and appeared to involve two distinct haplotypes (Fig. 1c). One haplotype was associated with the highest peak (chromosome 4 position 17497441; −log_10_*P* = 7.6; MAF = 5.3%), and a complete subset of the other haplotype was associated with the other SNP at chromosome 4 position 17486863 (−log_10_*P* = 5.47, MAF = 30.1%). GWAS accounting for the genetic variants at chromosome 4 position 17497441 did not affect the peak at *DDM1*; that is, the associations were independent (Extended Data Fig. 1g–i).

To gain further insight, we conducted GWAS for mCG levels of 6,379 individual TEs commonly found in all 774 lines and containing at least one CG site (Fig. 1d)^26^. Strong associations were primarily observed in *cis* (diagonal), suggesting either direct epigenetic inheritance of mCG or that genetic variation within TEs influences mCG levels, consistent with a previous study on differential mCG variation^38^. In addition, the vertical lines on the graph identified clear *trans* regulation of mCG levels in TEs distributed around pericentromeric regions associated with the SNPs on chromosome 4 discussed above (Fig. 1d). Chromosome 4 positions 17486863 and 17497441 showed associations with 229 and 116 TEs (threshold −log_10_*P* > 6, MAF > 5%), respectively, with a significant overlap (62% for 17486863 and 75% for 17497441) with CMT2-targeted TEs and almost no overlap (5.2% and 0%) with RdDM-targeted TEs.

Two haplotypes represented by chromosome 4 positions 17486863 and 17497441 included several genes but were centred on *AT4G37110* and *AT4G37120*. *AT4G37120* encodes SWELLMAP 2 (SMP2), a step II splicing factor that works redundantly with its homologue *SMP1* (ref. ^39^). *AT4G37110* contains a zinc-finger domain with homology to human CDCA7 (ref. ^40^) (Fig. 1e), which recruits Helicase, Lymphoid-Specific (HELLS)^31,41–43^, the human orthologue of DDM1, making it an excellent candidate for being the causal gene.

Further evidence for this hypothesis was provided by bisulfite sequencing of loss-of-function mutants for *SMP2*, *AT4G37110* and the neighbouring gene *AT4G37130* as control. Unlike the other genes, the *at4g37110* mutant showed significantly lower mCG levels at TEs identified as targets of the *trans* modifier using GWAS (Fig. 1f). These results strongly suggest that the allelic variation of *AT4G37110* is responsible for the identified association on chromosome 4 and that *AT4G37110* is an orthologue of human *CDCA7*.

### CDCA7α and CDCA7β redundantly maintain mCG and heterochromatin features to enforce silencing of TEs

**Fig. 2:**
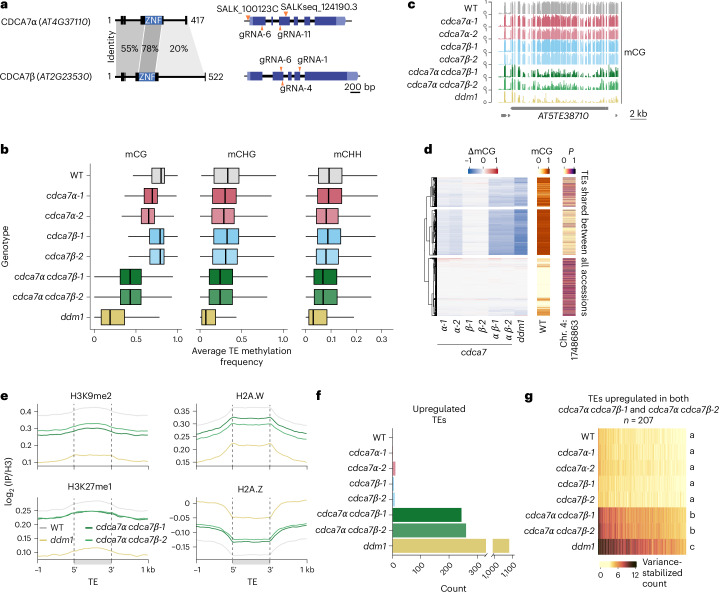
CDCA7α and CDCA7β redundantly maintain mCG, heterochromatin marks and transcriptional silencing. **a**, Protein (left) and gene (right) models for CDCA7α and CDCA7β, with sequence identity scores between domains. Secondary structures (boxes) and low-confidence regions (black lines; predicted local distance difference test (pLDDT), <70) are shown. The orange triangles mark T-DNA insertions and CRISPR–Cas9 guide RNAs (gRNAs). ZNF, zinc-finger domain. **b**, 5mC in the indicated contexts at TEs methylated in the CG, CHG or CHH contexts (*n* = 18,052, 16,739 and 18,037 TEs, respectively). The box plots show the median (centre line), the IQR (25th–75th percentiles; box limits) and the minimum and maximum values within 1.5× IQR (whiskers). Outliers are not plotted. **c**, Genome browser view of mCG levels in the indicated mutants. **d**, Heat map comparing ΔmCG (mutant − WT) for *cdca7* null mutations with the effect of *CDCA7α* natural alleles on mCG, using the association of the SNP at position 17486863 on chromosome 4 with TE mCG (*P*). The analysis was restricted to 6,145 TEs shared between all accessions (*n* = 774). *K*-mean clusters were defined on the basis of ΔmCG. The WT value is shown as a reference. **e**, Metaplots of histone marks and variants normalized to histone H3, at heterochromatic TEs (*n* = 12,548), defined on WT enrichment of H3K9me2, H3K27me1 and H2A.W. The mean is shown, and standard error are too small to be visible. **f**, TEs upregulated in the indicated mutants relative to the WT. **g**, Transcript levels of TEs upregulated in both *cdca7α* *cdca7β* mutants, shown with variance-stabilized counts. Groups not sharing the same letter are significantly different (two-sided Tukey’s HSD test, *P* < 0.05).

**Extended Data Fig. 2:**
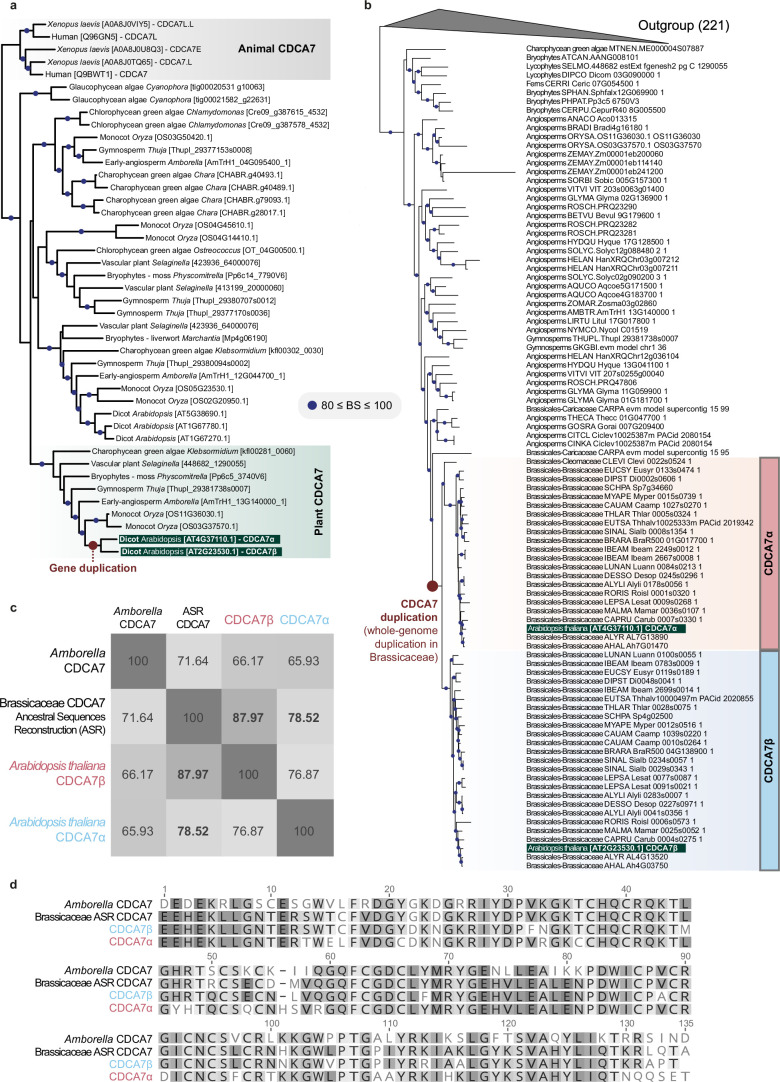
The evolutionary relationships of CDCA7 proteins across different evolutionary scales. **a**, The phylogeny of CDCA7 proteins among representative taxa of Archaeplastida. Xenopus and human CDCA7 proteins provided as an outgroup, previously identified as homologs of plant CDCA7^44^. **b**, The phylogeny of CDCA7 proteins among representative taxa of Viridiplantae, including multiple species from Brassicaceae. **c**, Protein identity comparison of the zinc finger domains of four CDCA proteins: *A. thaliana* CDCA7α and CDCA7β, ancestral sequence reconstruction (ASR) of CDCA7 protein sequence at the node of Brassicaceae CDCA7 duplication (n = 48), and *Amborella* CDCA7 as an outgroup. The average posterior probability of ASR sequence (total 406 residues) is 0.916 (n = 406, median=0.996, σ = 0.151). The zinc finger domain of CDCA7β shows a higher similarity to the ASR sequence compared to CDCA7α, indicating that CDCA7β may be more ancestral than CDCA7α. **d**, ASR of CDCA7 zinc finger aligned with three CDCA7 proteins.

Like *AT4G37110*, *AT2G23530* also contains a zinc-finger domain with high sequence homology to metazoan *CDCA7* (Fig. 1e), suggesting that there might be two bona fide C*DCA7* orthologues in *A. thaliana*^44^. Our study confirms this. Phylogenetic analysis revealed that *CDCA7* duplication occurred in Brassicaceae (Extended Data Fig. 2a,b), coinciding with a whole-genome duplication event^45^. These two *A. thaliana CDCA7* orthologues share similar expression patterns throughout development (Supplementary Fig. 2a). Given its higher expression, *AT4G37110* was designated *CDCA7α*, while *AT2G23530* was named *CDCA7β* (Fig. 2a). According to ancestral sequence reconstruction, the zinc-finger domain of *CDCA7β* is more similar to the ancestral gene than *CDCA7α* (Extended Data Fig. 2c,d). We found no evidence for allelic variation affecting mCG in *CDCA7β*.

**Extended Data Fig. 3:**
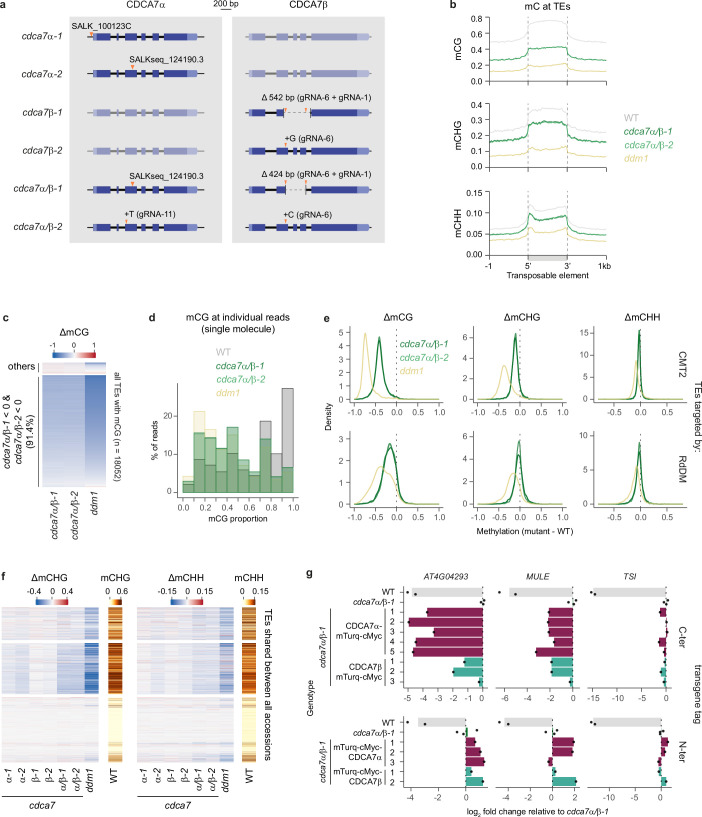
CDCA7α/β primarily affect the DNA methylation of CMT2-targeted TEs. **a**, Examples of mutations used in this study, drawn to scale. Additional *cdca7* mutations are listed in Supplementary Table 2. **b**, Metaplots of 5mC at TEs. Mean and standard error are shown. **c**, mCG changes in the indicated mutants, using all TEs with initial mCG > 5%. **d**, Distribution of average mCG for individual bisulfite reads. **e**, Distributions showing 5mC differences between the indicated mutants and the WT in CG, CHG and CHH contexts, distinguishing TEs targeted by CMT2 or RdDM. **f**, Heatmap comparing Δ5mC (mutant - WT) for *cdca7* null mutations. K-mean clusters were defined based on ΔmCG (Fig. 2c). **g**, RT-qPCR quantification of TE transcripts in *cdca7α/β-1* mutants complemented with a transgene containing a C- or N-terminal tag. Primary transformants were plotted individually to account for differences in transgene expression. Barplots show means, with black points showing biological replicates (n = 2 for WT, n = 3 for *cdca7α/β-1*). Expression levels are normalized to *ACTIN2* and *cdca7α/β-1*.

To investigate the functions of these CDCA7 paralogues, we generated single and double mutants for *cdca7α* and *cdca7β* using transfer DNA (T-DNA) insertions and CRISPR–Cas9, obtaining two independent lines per mutant to control for potential genetic aberrations (Fig. 2a and Extended Data Fig. 3a). CRISPR mutations affected *CDCA7α* expression to various degrees (Supplementary Fig. 2b), but all changes were out of frame (Supplementary Table 2) and led to premature stop codons. We compared *cdca7* mutants with *ddm1* mutants of the same generation (Methods), because the molecular defects caused by *ddm1* loss of function gradually accumulate over consecutive homozygous generations^46,47^.

TEs had reduced mCG levels in the two *cdca7α* mutant alleles, with a stronger effect in *cdca7α-2* (Fig. 2b,c). The T-DNA in *cdca7α-2* disrupts the coding sequence, whereas the T-DNA in *cdca7α-1* is located in the promoter and leads to CDCA7α overexpression, possibly affecting downstream factors (Extended Data Fig. 3a and Supplementary Fig. 2b). Single mutations in *CDCA7β* did not affect mCG levels, but *cdca7α* *cdca7β* double mutants had strongly decreased mCG compared with *cdca7α* (Fig. 2b,c), demonstrating that *CDCA7α* and *CDCA7β* redundantly maintain mCG levels. The *cdca7α* and *cdca7β* mutations had only minimal impacts on mCHG and mCHH levels, with a synergistic effect in *cdca7α* *cdca7β* (Fig. 2b). Compared with *cdca7α* *cdca7β*, the loss of DDM1 had a stronger impact in all contexts (Fig. 2b,c and Extended Data Fig. 3b). The weaker effect of *cdca7α* *cdca7β* was not due to a localized impact on a TE subset but instead due to an intermediate loss of methylation at most TEs, as 91.4% of them had decreased mCG in both *cdca7α* *cdca7β-1* and *cdca7α* *cdca7β-2* (Extended Data Fig. 3c), demonstrating that CDCA7α/β target nearly all TEs. Unlike the wild type (WT), both *cdca7α* *cdca7β* and *ddm1* had a low proportion of fully methylated bisulfite reads (Extended Data Fig. 3d), indicating that the intermediate mCG levels in *cdca7α* *cdca7β* reflected a partial reduction of mCG across most individual DNA molecules, arguing against a decrease restricted to certain cell types. Mirroring our GWAS results, the impact of *cdca7α* *cdca7β* was strongest at CMT2-dependent TEs (Extended Data Fig. 3e). Importantly, TEs with the strongest mCG decrease in *cdca7α* and *cdca7α* *cdca7β* also showed the most significant association of the *CDCA7α* SNP with mCG variation at CMT2-dependent TEs (Fig. 2d and Extended Data Fig. 3f), indicating that null and natural alleles of *CDCA7α* impact the same targets.

Because DDM1 directly controls the genomic distribution of histones H2A.W and H2A.Z, and affects the levels of dimethylated histone H3 lysine 9 (H3K9me2) and monomethylated H3 lysine 27 (H3K27me1) histone post-translational modifications^32,48–51^, we profiled these chromatin marks in *ddm1* and two *cdca7α* *cdca7β* mutants using chromatin immunoprecipitation followed by sequencing (ChIP–seq), focusing on heterochromatic TEs (Methods). Our results showed that *cdca7α* *cdca7β* mutants exhibited decreased levels of H3K9me2, H3K27me1 and H2A.W, while H2A.Z levels increased, resembling *ddm1* but with a milder effect (Fig. 2e).

Furthermore, we quantified the impact of *cdca7* mutations on TE silencing using 3′-tag RNA-seq. Single mutations in *cdca7α* or *cdca7β* had negligible effects, but the double mutants showed over 200 upregulated TEs (Fig. 2f). The extent of TE upregulation in *cdca7α* *cdca7β* was less than in *ddm1* (Fig. 2f,g). To further validate these observations, *cdca7α* *cdca7β-1* was complemented with *CDCA7α* or *CDCA7β*, fused with an amino- or carboxy-terminal tag containing 3xcMyc and mTurquoise2. Constructs with a C-terminal tag partially complemented silencing defects (Extended Data Fig. 3g), confirming the role of *CDCA7α*/*β* in TE silencing. Interestingly, the N-terminal tag inhibited *CDCA7α*/*β*-mediated silencing, suggesting that it interfered with a critical CDCA7 function.

**Extended Data Fig. 4:**
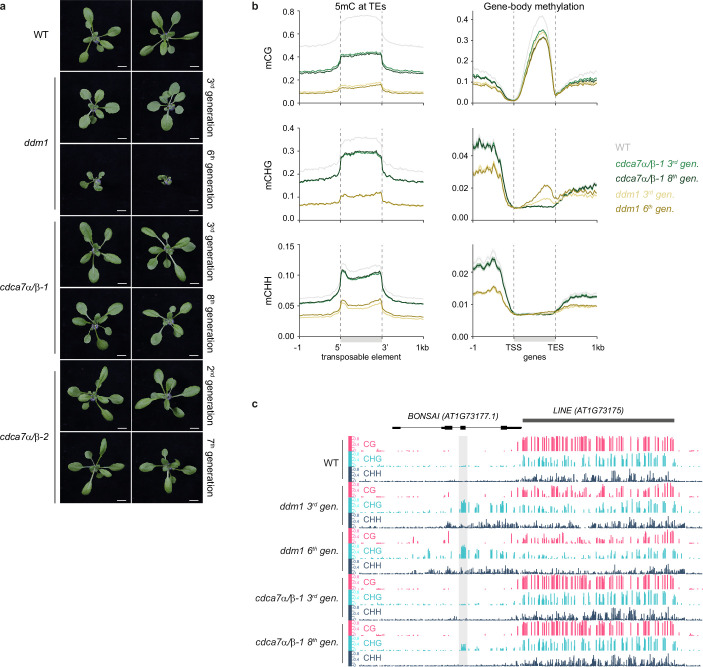
Progressive hypomethylation upon selfing of *cdca7α/β* mutants but no phenotypic aggravation. **a**, Developmental phenotypes of the indicated mutants after 20 days of growth in long days. Scale bar: 10 mm. **b**, Metaplots of 5mC at TEs and genes with gene-body methylation. Mean and standard error are shown. **c**, Genome browser view showing *BONSAI* hypermethylation in the indicated samples.

Unlike *ddm1* mutants, which show developmental defects upon inbreeding^47^, *cdca7α* *cdca7β* mutants maintained a normal phenotype even after eight generations of inbreeding (Extended Data Fig. 4a). Because inbreeding *ddm1* mutants causes progressive mCG reduction and mCH hypermethylation^46^, we assessed generational DNA methylation changes in *cdca7α* *cdca7β-1*. Both *cdca7α* *cdca7β-1* (eighth generation) and *ddm1* (sixth generation) mutants showed decreased mCG levels at TEs and genes, and increased mCHH at TEs, compared with their third-generation counterparts (Extended Data Fig. 4b). However, a critical distinction emerged: ectopic mCHG accumulation at gene-body methylated genes, a hallmark of the *ddm1* *bonsai* (*bns*) phenotype^46,52^, occurred exclusively in *ddm1* mutants. In *cdca7α* *cdca7β*, there was no genic CHG hypermethylation on average, but we could detect a local mCHG increase at the third exon of the *BSN* gene (Extended Data Fig. 4c). These findings suggest that the absence of widespread genic CHG hypermethylation in eighth-generation *cdca7α* *cdca7β* mutants prevents the onset of developmental defects, though further inbreeding could eventually trigger a developmental phenotype through *BSN* hypermethylation.

In summary, *CDCA7α* and *CDCA7β* redundantly maintain heterochromatin marks and TE silencing. *CDCA7α* and *CDCA7β* appear to have identical functions, but *CDCA7α* plays a more prominent role than *CDCA7β*, possibly due to its higher expression (Supplementary Fig. 2a).

### CDCA7α and CDCA7β function with DDM1 to maintain mCG at TEs and gene bodies

**Fig. 3:**
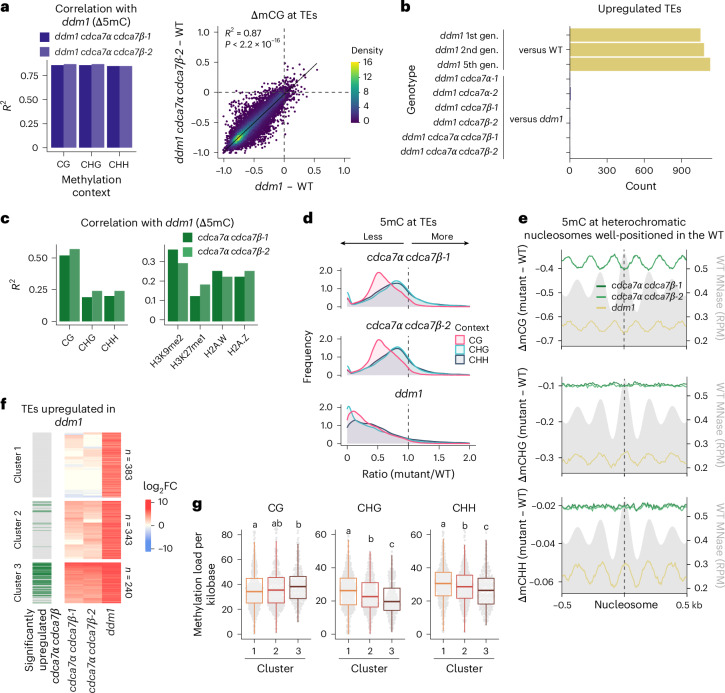
CDCA7α and CDCA7β function through DDM1 to maintain mCG. **a**, Linear correlation of 5mC loss at TEs in *ddm1* *cdca7α* *cdca7β* compared with *ddm1*. Left: squared Pearson correlation coefficients (*R*^2^) for all contexts are represented. All correlations had *P* < 1 × 10^−16^ (two-sided permutation test). Right: an example of mCG loss at TEs between *ddm1* *cdca7α* *cdca7β-2* and *ddm1*. **b**, Number of TEs upregulated in the indicated mutants relative to the WT or *ddm1*. Mutant combinations of *ddm1* and *cdca7* were compared to *ddm1* mutants of the closest generation (gen.). **c**, Linear correlations between *ddm1* and the indicated mutants for 5mC loss (left) and chromatin mark changes (right). Squared Pearson correlation coefficients (*R*^2^) are shown; all correlations had *P* < 1 × 10^−10^ (two-sided permutation test). **d**, Distribution of methylation ratios for TEs (mutant/WT) across genotypes and 5mC contexts. The *x* axis is capped at 2, excluding at most 3.2% of the data. **e**, Metaplots showing 5mC around well-positioned nucleosomes at heterochromatic TEs (*n* = 22,515 nucleosomes). The values are normalized to the WT to compare the impact of mutations on 5mC periodicity. The mean are shown. MNase-seq, shown as reads per million (RPM), and nucleosome positions are publicly available^54^. **f**, Changes in transcript levels of DDM1-silenced TEs (*n* = 966). *K*-mean clustering was performed on *cdca7α* *cdca7β-1* and *cdca7α* *cdca7β-2* log_2_ fold change (FC) values. TE upregulation thresholds in both mutants are highlighted with green horizontal bars. **g**, WT methylation load per kilobase (Methods) at the TE clusters defined in **f**. Groups not sharing the same letter are significantly different (two-sided Dunn tests with Benjamini–Hochberg correction, *P* < 0.05). The box plots show the median (centre line), the IQR (25th–75th percentiles; box limits) and the minimum and maximum values within 1.5× IQR (whiskers). Source data

**Extended Data Fig. 5:**
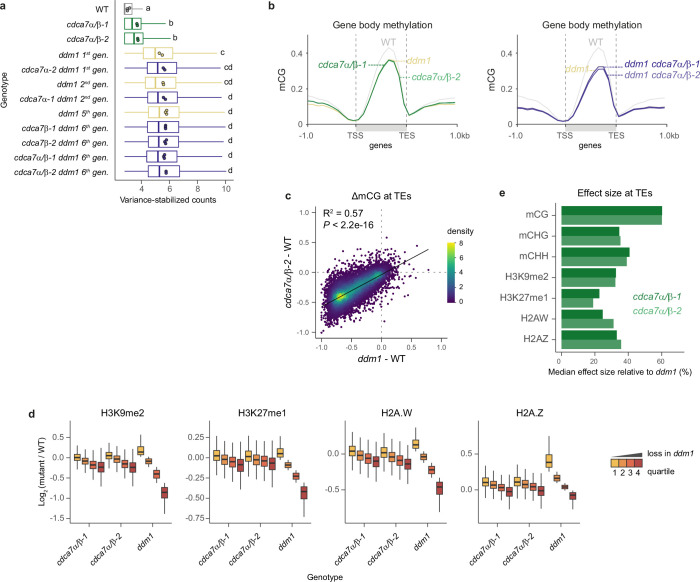
CDCA7α and CDCA7β function through DDM1 to maintain mCG. **a**, Variance-stabilized transcript levels of TEs upregulated in any of the *ddm1* mutants (yellow, n = 1147). Boxplots show the distribution of transcript levels for individual TEs, while mean expression values for all TEs are shown as circles for each biological replicate. Groups not sharing the same letter are significantly different (two-sided Tukey’s HSD test, *P* < 0.05). **b**, Metaplot showing mCG at gene-body methylated genes (n = 9938). Mean and standard error are shown. **c**, Correlation of mCG loss at TEs in *cdca7α/β-2* relative to *ddm1*. Squared Pearson correlation coefficients (R^2^) and a two-sided permutation test-derived *P*-value are shown. **d**, Changes in histone marks and variants at heterochromatic TEs (n = 12548), stratified into quartiles based on the change observed in *ddm1*. **e**, Effect size of *cdca7α/β* mutations relative to *ddm1* across various metrics. In **a** and **d**, box plots display the median (centre line), with the box defining the IQR (25th–75th percentiles). Whiskers extend to a maximum of 1.5 x IQR, and outliers are not plotted. Source data

At the molecular level, *cdca7α* *cdca7β* null mutants resembled *ddm1* (Fig. 2), suggesting that in *Arabidopsis* CDCA7 orthologues and DDM1 operate in a common pathway. To test this genetically, we combined *ddm1* and *cdca7α* *cdca7β* mutations and measured their impact on 5mC and TE silencing. The triple *cdca7α* *cdca7β* *ddm1* mutants showed 5mC levels comparable to those of *ddm1* mutants of the same generation (Fig. 3a). Additionally, introducing any *cdca7* mutation alongside *ddm1* did not affect TE transcript levels (Fig. 3b and Extended Data Fig. 5a). We therefore concluded that CDCA7 orthologues act entirely through DDM1 to regulate 5mC and TE silencing.

The milder effect of *cdca7α* *cdca7β* mutations than *ddm1* (Fig. 2) implies that DDM1 can partially function without CDCA7 orthologues. Looking at gene-body methylation, however, we found that the decrease in mCG was of similar magnitudes in *cdca7α* *cdca7β* and *ddm1*, and in *ddm1* and *cdca7α* *cdca7β* *ddm1* mutants of advanced generations (Extended Data Fig. 5b), showing that DDM1’s role in promoting gene-body methylation is fully reliant on CDCA7α/β.

DDM1 has distinct functions in heterochromatin maintenance: first, depositing H2A.W while preventing H2A.Z deposition^48,51,53^; and second, facilitating access of DNA methyltransferase to nucleosomal DNA through nucleosome remodelling^37,54^. Heterochromatic histone modifications also depend on DDM1, through unknown mechanisms^32,48–50^. CDCA7α/β might therefore differentially affect specific activities of DDM1. To explore this, we compared how *ddm1* and *cdca7α* *cdca7β* mutations affect 5mC, histone variants and modifications. The effects of the mutations had strong linear correlations for mCG (Fig. 3c and Extended Data Fig. 5c; *R*^2^ = 0.52–0.57) but weak ones for mCH (*R*^2^ = 0.19–0.24). Correlations were intermediate for H3K9me2 (Fig. 3c; *R*^2^ = 0.30–0.37), probably reflecting the dependence of H3K9me2 on both mCH^55^ and mCG^56–59^. For H3K27me1 and histone variants H2A.W and H2A.Z, correlations were low, although the overall trends resembled those in *ddm1* (Extended Data Fig. 5d). Therefore, *cdca7α* *cdca7β* mutants primarily resembled *ddm1* in their effect on mCG but not on mCH, histone variant deposition or histone modifications, suggesting that the primary function of the CDCA7α/β–DDM1 pathway is to promote mCG. Strikingly, *cdca7α* *cdca7β* mutants were disproportionately affected in the CG context, whereas all 5mC contexts were equally affected in *ddm1* (Fig. 3d), highlighting the specific role of CDCA7α/β in regulating mCG at TEs. We further quantified the relative contribution of CDCA7α/β to DDM1 functions by comparing the effect size of *cdca7α* *cdca7β* to that of *ddm1* on different heterochromatin features. In contrast with the milder effect on other marks, the loss of CDCA7α/β accounted for 60% of the mCG changes observed in *ddm1* mutants (Extended Data Fig. 5e). Likewise, GWAS pointed towards a main role of CDCA7α/β in mCG regulation (Extended Data Fig. 1b,d). Taken together, these data demonstrate that while DDM1 plays a central role in maintaining multiple heterochromatic marks, CDCA7α/β are primarily involved in mCG maintenance.

**Extended Data Fig. 6:**
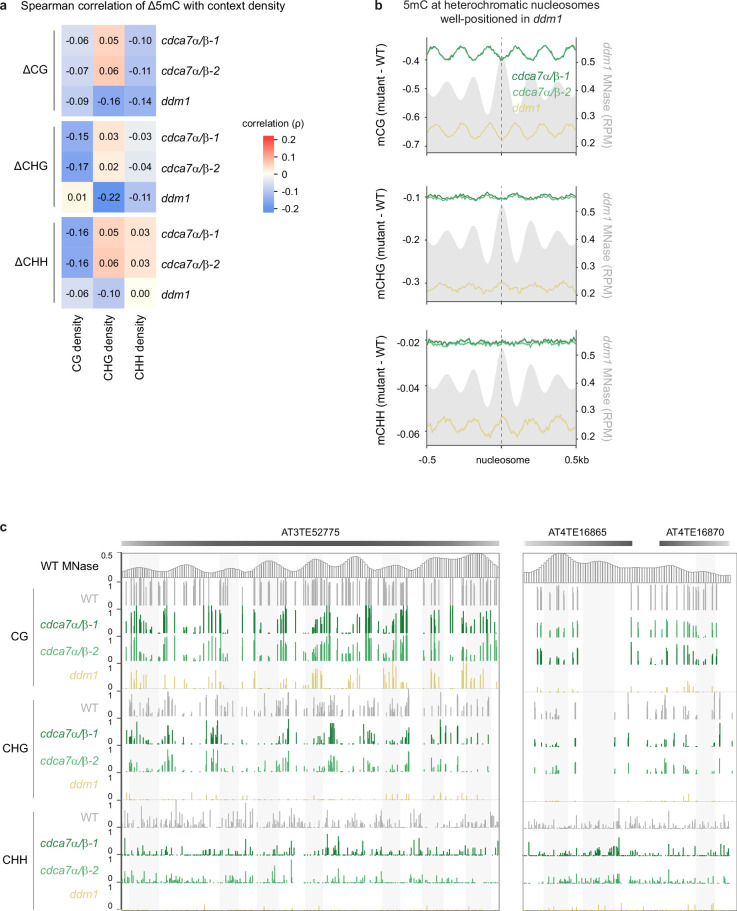
CDCA7α and CDCA7β predominantly maintain mCG. **a**, Spearman correlations coefficients (ρ) of methylation context density (number of CN sites per bin) with 5mC loss in mutants, calculated in 100 bp bins. **b**, Metaplots showing 5mC changes around *ddm1* well-positioned nucleosomes found at heterochromatic TEs (n = 21844). MNase-seq data and nucleosome positions are from^54^. Mean and standard error are shown. **c**, Genome browser views showing *cdca7α/β* methylome data with published WT MNase-seq data (replicate 2 is shown, RPM)^54^. Well-positioned WT nucleosomes are indicated with grey rectangles.

In *met1* mutants, mCG loss leads to the depletion of mCHG, mCHH and H3K9me2, with a concurrent H2A.Z increase^50,57,59–61^, whereas its effects on H3K27me1 and H2A.W are unknown. This suggests that the moderate changes in levels of mCH, H2A variants and H3 modifications in *cdca7α* *cdca7β* mutants are secondary consequences of reduced mCG. Supporting this interpretation, we found that mCHG and mCHH changes in *cdca7α* *cdca7β* mutants were anticorrelated with the density of CG sites (Extended Data Fig. 6a). This differed from *ddm1*, where mCH loss correlated best with CHG density, suggesting that mCH maintenance by DDM1 is mostly independent of CDCA7α/β.

DDM1 promotes the methylation of nucleosome-bound DNA^54^. To determine whether CDCA7α/β contributes to this process, we examined how *cdca7α* *cdca7β* mutations affect 5mC around nucleosomes. Using nucleosome maps from the WT and *ddm1* (ref. ^54^), we found that both *cdca7α* *cdca7β* and *ddm1* mutants lose mCG preferentially within nucleosome cores, whereas adjacent linker DNA is less affected (Fig. 3e, Extended Data Fig. 6b,c and Supplementary Fig. 3). The pattern of mCH loss differed between mutants: *ddm1* showed the greatest reduction in linker regions, whereas *cdca7α* *cdca7β* mutants lost mCH uniformly across both linker and nucleosomal DNA. These results indicate that CDCA7α/β and DDM1 act through a common pathway to maintain nucleosomal mCG, but that the reduction in mCH observed in *ddm1* is driven by a separate mechanism, independent of CDCA7α/β. Together, these data support a model in which CDCA7α/β predominantly maintain mCG at nucleosomal DNA in a DDM1-dependent manner.

**Extended Data Fig. 7:**
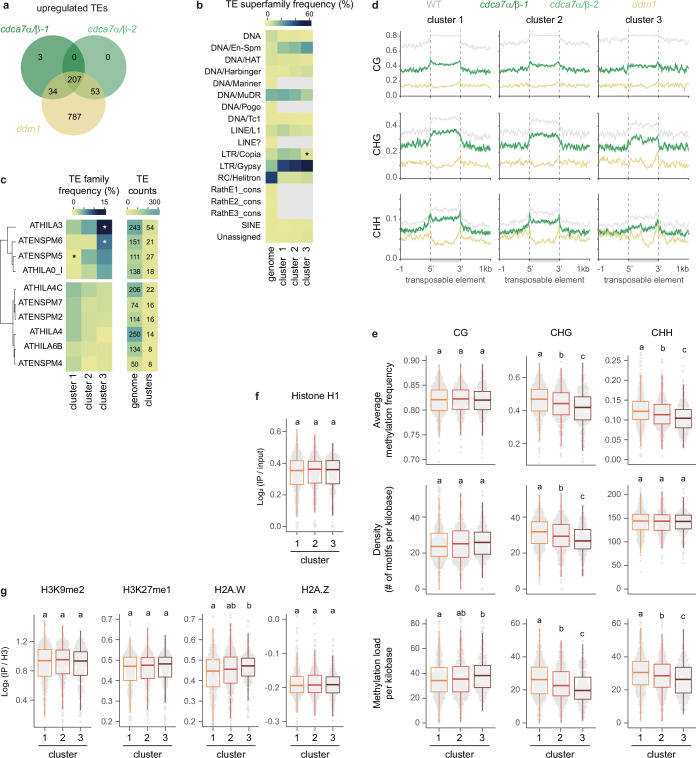
CDCA7α/β silence TEs with high mCG and low mCH abundance. **a**, Overlap of upregulated TEs between mutants. **b**, **c**, Frequency of TE superfamilies and families at *ddm1*-upregulated TEs, clustered in Fig. 3f. Significant differences between cluster frequency and background frequency (other clusters) were evaluated with a two-sided Fisher’s exact test with Benjamini-Hochberg correction (*P* < 0.05). **c**, Only the ten families with most divergent frequencies between cluster 1 and 3 were selected. The absolute number of TE counts in the genome and all clusters is shown. **d**, Metaplots of 5mC at TEs clustered in Fig. 3f. Mean and standard error are shown. **e**, **f**, **g**, DNA methylation metrics (**e**) and WT levels of histone modifications and variants (**f**, **g**) at *ddm1*-upregulated TEs (n = 966), clustered in Fig. 3f. Methylation load per kilobase is reproduced from Fig. 3g. Groups not sharing the same letter are significantly different (two-sided Dunn tests with Benjamini-Hochberg correction, *P* < 0.05). Histone H1 data is from^146^. Box plots display the median (centre line), with the box defining the IQR (25th–75th percentiles). Whiskers extend to a maximum of 1.5 x IQR and the y-axis is zoomed-in, excluding some outliers from the representation. Source data

We next asked why only a subset of the TEs derepressed in *ddm1* are also upregulated in *cdca7α* *cdca7β* (Fig. 2 and Extended Data Fig. 7a). To address this, we clustered those elements by their log_2_ fold change in *cdca7α* *cdca7β* (Fig. 3f) and compared their features. While TE length and GC content did not vary, TEs sensitive to *cdca7α* *cdca7β* were closer to centromeres (Supplementary Fig. 4a,b). Accordingly, they were enriched for the mostly centromeric En-Spm and LTR Gypsy superfamilies^62^ and depleted for the broadly distributed Copia and MuDR elements (Extended Data Fig. 7b). At the family level, the degree of enrichment progressively followed CDCA7α/β dependency (Extended Data Fig. 7c). The loss of CDCA7α/β caused the largest reduction in heterochromatic features at TEs silenced by CDCA7α/β (Supplementary Fig. 4c,d). For mCG, this reduction was most pronounced immediately upstream of the TE 5′ boundary (Extended Data Fig. 7d), suggesting a localized effect at TE promoters, which could be either the cause or the consequence of TE upregulation. Interestingly, TEs that were not upregulated in *cdca7α* *cdca7β* had higher basal levels of mCHG and mCHH and more frequent CHG sites, suggesting that abundant mCH can maintain silencing in the absence of CDCA7α/β (Extended Data Fig. 7e). Calculating the average number of fully methylated cytosines per kilobase at TEs (DNA methylation load; Methods), we found higher mCG loads and lower mCH loads at *cdca7α* *cdca7β*-sensitive TEs (Fig. 3g). These loci also showed the highest H2A.W enrichment (Extended Data Fig. 7f,g), possibly reflecting their pericentromeric enrichment. Collectively, these data show that the dependency of TE silencing on CDCA7α/β is governed by the balance between mCG and mCH. Elements with a high mCG-to-mCH ratio require CDCA7α/β for continued silencing, whereas TEs rich in mCH remain repressed even when mCG decreases in the absence of CDCA7α/β. The data therefore support a specialized role for CDCA7 paralogues in the maintenance of mCG at TEs and gene bodies.

### CDCA7 interacts with the DDM1 H2A.W-binding region

**Fig. 4:**
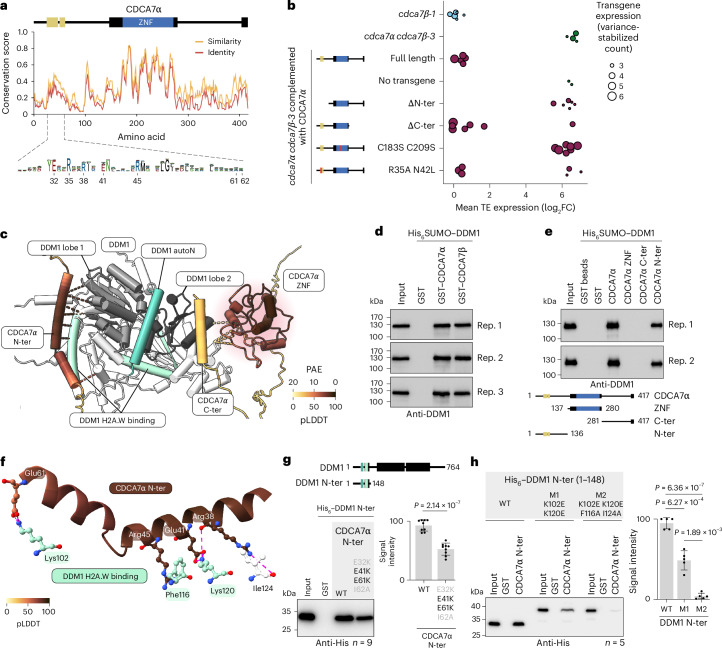
Characterization of CDCA7α/β protein domains and interface with DDM1. **a**, Conservation of CDCA7α among 315 orthologues in Viridiplantae, with domains represented to scale, including the DDM1-interacting α-helices (yellow). A consensus sequence (bottom, *n* = 37) highlights residues predicted to contact DDM1. **b**, TE upregulation in *cdca7α* *cdca7β-3* complemented with various CDCA7α constructs. Each data point is a T_1_ plant, showing the mean log_2_FC (relative to the WT) of TEs upregulated in *cdca7α* *cdca7β-3*, scaled by transgene expression. **c**, Predicted CDCA7α–DDM1 structure, showing predicted contacts ≤3 Å coloured by predicted aligned error (PAE). CDCA7α is coloured by local confidence, using pLDDT. **d**, In vitro pulldown of recombinant CDCA7α and CDCA7β with DDM1, detected via western blot. Three pulldown replicates (rep.) with similar results are shown. **e**, Pulldown of recombinant DDM1 with truncated CDCA7α constructs. Two pulldown replicates with similar results are shown. **f**, Predicted interaction of the CDCA7α N-terminal α-helices with the DDM1 N-terminal part. CDCA7α is coloured by pLDDT, with interacting residues shown in a ball-and-stick representation. Hydrogen bonds and salt bridges ≤4 Å are shown as dashed lines. **g**,**h**, Pulldown of recombinant CDCA7α N terminus (ter) with DDM1 N terminus. The effect of mutations in the CDCA7α N terminus (**g**) and the DDM1 N terminus (**h**). The blots shown are representative of nine and five pulldown replicates for **g** and **h**, respectively, and the quantification shows averages with standard deviations. Differences were evaluated with a two-sided Student’s *t*-test, correcting for multiple testing with Bonferroni’s method for **h**. Source data

Next, we explored the function of the three CDCA7α/β domains using a genetic complementation strategy. The N-terminal domain contains a highly conserved motif (Fig. 4a), the 4CXXC zinc-finger domain binds hemi-methylated CG dinucleotides^31,43,63^ and the C-terminal part of the protein has lower conservation.

**Extended Data Fig. 8:**
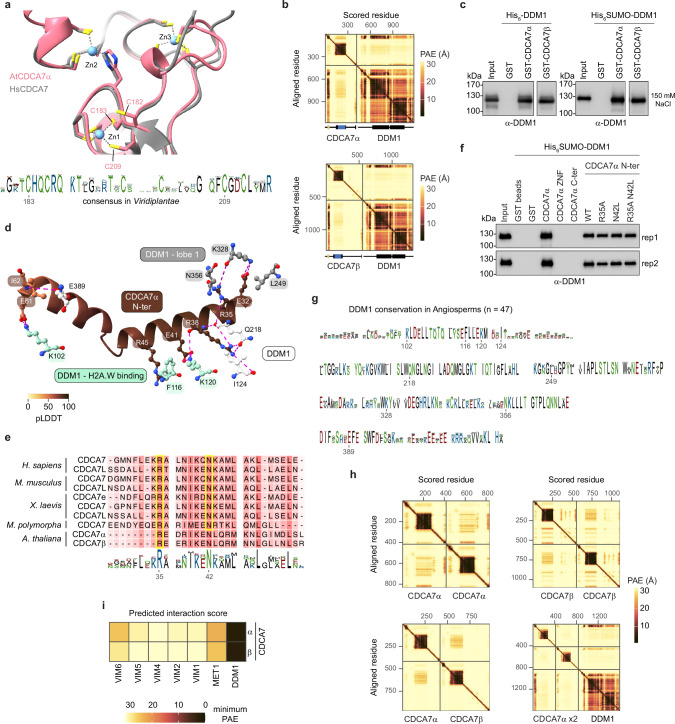
Physical interaction between CDCA7α/β and DDM1. **a**, Alphafold3 prediction of *A. thaliana* CDCA7α zinc finger domain aligned to *H. sapiens* CDCA7 crystal structure (PDB ID: 8TLK). The zinc atoms and their binding residues are shown. The consensus sequence in Viridiplantae is shown at the bottom (n = 37). **b**, Predicted Aligned Error (PAE) from AlphaFold3, modeling the interaction between DDM1 and CDCA7α (top) or CDCA7β (bottom). Protein domains are drawn at the bottom. **c**, In vitro pulldown of recombinant CDCA7α and CDCA7β with DDM1, at 150 mM NaCl. The two pulldowns shown, with different DDM1 tags, gave similar results. **d**, Predicted interaction of CDCA7α N-terminal alpha helices with DDM1. CDCA7α is colored by local confidence, using predicted local distance difference test (pLDDT). Interface residues are shown in a ball-and-stick representation, with hydrogen bonds and salt bridges (≤ 4 Å) indicated as dashed lines. **e**, Alignment of CDCA7 proteins from model species. A yellow box highlights CDCA7 residues predicted to interact with HELLS/DDM1 from the corresponding species. The position of CDCA7α R35 and N42 is indicated on the consensus sequence. **f**, In vitro pulldown of recombinant DDM1 with truncated CDCA7α and mutated N-ter of CDCA7α. A cropped version of this data is shown in Fig. 4e. Two pulldown replicates shown gave similar results. **g**, Conservation in Angiosperms (n = 47) of DDM1 residues predicted to interact with CDCA7α. Numbers indicate residues with predicted contact to CDCA7α. **h**, Alphafold3 PAE plot to predict different protein-protein interactions between CDCA7α, CDCA7β and DDM1. **i**, Alphafold3 minimum PAE score to predict different protein-protein interactions between CDCA7α, CDCA7β and the indicated mCG-regulating proteins. Source data

The global loss of mCG in *met1* or *ddm1* mutants is largely irreversible even after complementation^64–67^, probably because of the semi-conservative nature of mCG maintenance. Accordingly, complementation of *cdca7α* *cdca7β-1* was partial and locus-specific (Extended Data Fig. 3g). To bypass this limitation, we transformed *cdca7α-2* with a *CDCA7α* transgene before the complete loss of endogenous *CDCA7α/β* (Supplementary Fig. 5a,b). We generated transcriptomes from individual T_1_ plants to quantify TE transcripts and transgene expression, which is highly variable between primary transformants^68^. Untransformed *cdca7β-1* and *cdca7α* *cdca7β-3* mutants showed no or few reads mapping to the transgenic tag, reflecting background noise (Fig. 4b). As expected, a full-length CDCA7α transgene restored TE silencing, while sister plants lacking the transgene had elevated TE transcript levels. CDCA7α(ΔC-ter) complemented silencing defects in individuals with high transgene expression, indicating that the C-terminal domain is dispensable for TE silencing. An N-terminal truncation could not be evaluated due to the lack of transgene expression (Fig. 4b). To test the function of the zinc-finger domain, we altered the conserved cysteines Cys183 and Cys209, which coordinate a zinc ion (Extended Data Fig. 8a). The lack of complementation with this construct (Fig. 4b) indicates that the CDCA7α zinc-finger is essential for TE silencing.

To further explore the function of CDCA7α/β domains, we used AlphaFold-Multimer^69^ to model potential interactions with DDM1. High-confidence interactions were identified between CDCA7α/β N termini and DDM1, specifically the H2A.W-binding domain and the N-terminal helicase domain (lobe 1; Fig. 4c and Extended Data Fig. 8b). In vitro pulldown assays with purified recombinant proteins showed that both CDCA7α and CDCA7β bind to DDM1 (Fig. 4d), and these interactions were maintained under more restrictive salt concentrations (Extended Data Fig. 8c). The N-terminal region of CDCA7α alone was sufficient to interact with DDM1 (Fig. 4e), whereas neither the zinc-finger nor the C-terminal part showed an interaction. Remarkably, the N-terminal residues predicted to interact with DDM1 (Fig. 4f and Extended Data Fig. 8d) were highly conserved across the green lineage (Fig. 4a). CDCA7α Arg35 and Asn42 were also conserved in model vertebrates and predicted to interact with HELLS (a DDM1 orthologue) (Extended Data Fig. 8e). However, R35A and N42L substitutions did not disrupt binding to DDM1 (Extended Data Fig. 8f) or the CDCA7α silencing function (Fig. 4b), indicating that other CDCA7α residues can mediate DDM1 binding in *A. thaliana*. Similarly, a CDCA7α N-terminal fragment with four substitutions (E32K, E41K, E61K and I62A) did not reduce DDM1 binding (Supplementary Fig. 6a), suggesting that other contacts compensate or that the model inaccurately predicts some contacts. When we isolated the interface between CDCA7α (1–136) and DDM1 (1–148) N termini, the same four substitutions now reduced the binding to 50% of the WT (Fig. 4g and Supplementary Fig. 6b). Reciprocally, replacing the conserved complementary lysines on the DDM1 N terminus (K102E and K120E) produced an equivalent 50% loss of affinity (Fig. 4h, Extended Data Fig. 8g and Supplementary Fig. 6c). Further altering the remaining predicted contact sites (Phe116 and Ile124) led to a near-complete abrogation of CDCA7α binding. Although these DDM1 mutants migrated anomalously on SDS–PAGE, intact-mass liquid chromatography-electrospray ionization-mass spectrometry (LC-ESI-MS) closely matched their expected masses (Supplementary Fig. 6d,e), demonstrating that the proteins are full-length; this suggests that the mobility shift reflects altered SDS binding. Taken together, our data show the direct interaction between the N-terminal ends of CDCA7α and DDM1, and we further identified four critical interaction residues via in vitro mutagenesis binding assays, although the CDCA7α N-terminal region probably has other contacts with DDM1 (Extended Data Fig. 8d and Supplementary Fig. 6a). These four DDM1 residues lie within the H2A.W-binding module^48^, raising the possibility that CDCA7α and H2A.W compete for DDM1 binding.

Further modelling indicated that CDCA7α/β paralogues cannot bind to each other or themselves, and that DDM1 can bind only one CDCA7α/β paralogue at a time (Extended Data Fig. 8h), consistent with CDCA7α/β acting redundantly (Fig. 2). Furthermore, we found no predicted interaction with MET1 or VIM1/2/4/5/6 (ref. ^19^) that could explain the specificity of CDCA7α/β to mCG (Extended Data Fig. 8i).

In summary, the C-terminal domain of CDCA7α does not contribute to its silencing function, while the zinc-finger is essential. In both CDCA7α and vertebrate CDCA7, the zinc-finger binds hemi-methylated CG^31,43,63^, implying that CDCA7α/β recruit DDM1 to hemi-methylated CG sites, facilitating mCG deposition after DNA synthesis. This recruitment is mediated by a conserved N-terminal domain of CDCA7, which also interacts with HELLS in vertebrates^31,43^. Combined with our previous results (Figs. 2 and 3), our biochemical data indicate that the H2A.W-binding domain of DDM1 interacts directly with the N-terminal domain of either CDCA7α or CDCA7β, and this interaction is crucial for maintaining mCG and repressing TEs.

### Genetic variation in the *CDCA7*α promoter shapes large mCG variation

We next investigated the molecular causes of the *CDCA7α* natural alleles. Although protein sequences showed a cysteine-to-serine substitution at position 182, this cysteine is not conserved in sister species (Supplementary Fig. 7) or in eukaryotes^44^, and it does not coordinate a zinc atom (Extended Data Fig. 8a). There were no amino acid changes in conserved sites within the zinc-finger domain or the DDM1-interacting domain, suggesting that a causal effect on protein activity was unlikely (Supplementary Fig. 7). Therefore, to explore the impact of natural genetic variation on *CDCA7α* regulation, we performed GWAS for *CDCA7α* expression using published transcriptome data of 427 accessions^23,70^.

**Fig. 5:**
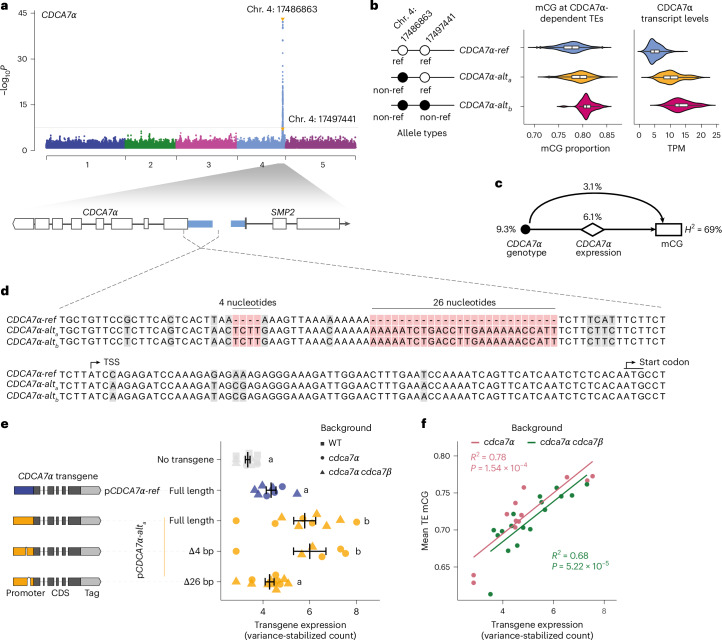
*Cis* polymorphism regulates CDCA7α promoter activity and shapes mCG variation. **a**, GWAS for *CDCA7*α expression. SNPs identified in GWAS for mCG are highlighted with orange arrow heads. GWAS was conducted using a linear mixed model with correction of population structure. The horizontal line indicates the Bonferroni-corrected significance threshold (*P* = 0.05). **b**, The allelic effects of *CDCA7*α on mCG and CDCA7α transcript levels. The violin plots and box plots show the mean mCG levels of CDCA7α-targeted TEs (*n* = 774 lines) and *CDCA7*α expression for each genotype (*n* = 461 lines) in transcripts per million (TPM). The box plots show the median (centre line), the IQR (25th–75th percentiles; box limits), the minimum and maximum values within 1.5× IQR (whiskers) and outliers (points beyond the whiskers). **c**, A mediation model showing the genetic effect of *CDCA7*α alleles on mCG through *CDCA7α* expression. The black dot, diamond and rectangle indicate genotype, gene expression and mCG phenotype, respectively. The arrows show the regulation. *H*^2^, SNP-based heritability. **d**, Sequence alignment around *CDCA7α*’s transcription start site (TSS) in three haplotypes. **e**, Effect of *CDCA7α* promoters on transgene expression, measured via mRNA-seq in complemented *cdca7α* *cdca7β-3* and *cdca7α-2* mutants. Transgenic constructs are shown to the left (to scale, except deletions). Each data point is an individual primary transformant with mean values ± standard error shown in black. Groups not sharing the same letter are significantly different (one-way analysis of variance with two-sided Tukey’s HSD post-hoc test, *P* < 0.05). The sample sizes (*n*) for the groups from top to bottom are 12, 8, 10, 6 and 12. **f**, Correlation of transgene expression with mean TE mCG. Squared Pearson correlation coefficients and *P* values (two-sided permutation test) are shown. Source data

Remarkably, a strong *cis* effect was detected for *CDCA7α* expression, with the most significant SNP identified as chromosome 4 position 17486863 (*CDCA7α-alt*_*a*_ haplotype) (−log_10_*P* = 42.10, MAF = 0.33), the same allele identified in our GWAS for mCG levels (Figs. 1a–d and 5a). Additionally, chromosome 4 position 17497441_non-ref_ (*CDCA7α-alt*_*b*_ haplotype) was also present in this large peak (−log_10_*P* = 5.68, MAF = 0.06). Both haplotypes were associated with two to three times higher *CDCA7α* expression than the reference allele Col-0, as well as elevated mCG levels (Fig. 5b).

To test for a causal link between DNA methylation and *CDCA7α* expression, we performed a mediation analysis^71^. This showed that 65.6% of the *CDCA7α* genetic effect was mediated by *CDCA7α* expression, suggesting that overexpression of *CDCA7α* in the *CDCA7α-alt*_*a*_ and *CDCA7α-alt*_*b*_ haplotypes contributes to the divergence of mCG levels (Fig. 5c). *CDCA7α* expression did not vary more than that of *DDM1* (the coefficients of variation were 55.6% and 67.8%, respectively), but *CDCA7α* expression had the most significant correlation with mCG levels at CMT2-targeted TEs (Spearman’s *ρ* = 0.34, *P* = 9.3 × 10^−13^), whereas DDM1 did not show a significant correlation (*ρ* = 0.07, *P* = 0.19), indicating that the dosage of *CDCA7α*, but not *DDM1* transcripts, regulates mCG. Alignments of the *CDCA7α* promoter region using fully assembled genomes revealed that the region is unusually polymorphic. Two indels, in complete linkage disequilibrium with those SNPs, distinguished *CDCA7α-alt*_*a*_ and *CDCA7α-alt*_*b*_ from the reference-type haplotype (*CDCA7α-ref*) (Fig. 5d). This suggests that different haplotypes in the *CDCA7α* promoter region change *CDCA7α* expression, resulting in diverse mCG levels in wild populations of *A. thaliana*.

**Extended Data Fig. 9:**
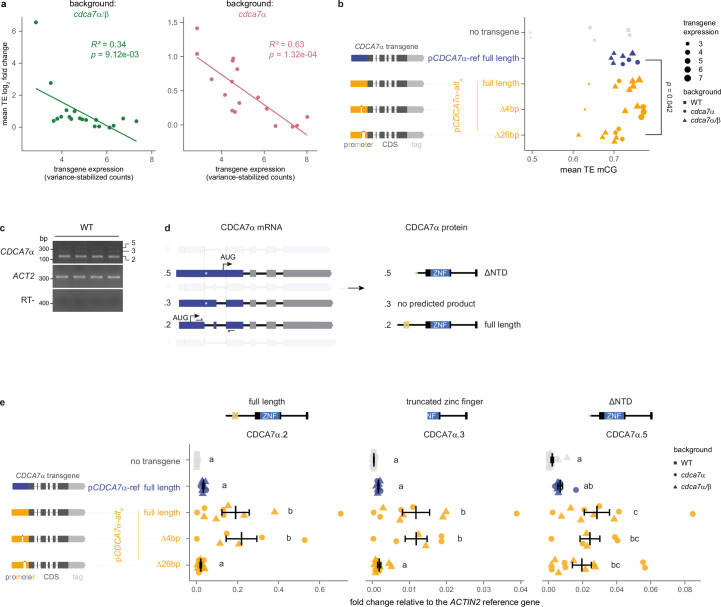
The 26 bp indel in the *CDCA7α* promoter regulates transcript isoform usage. **a**, Correlation of transgene expression with mean TE log_2_(fold change) relative to WT. Squared Pearson correlation coefficients and *p*-values (two-sided permutation test) are shown. **b**, Mean mCG at TEs in complemented *cdca7α/β* and *cdca7α* mutants. Each data point is an individual plant, with point size indicating transgene expression levels. The *p-value* is from a two-sided Student’s test. **c**, *CDCA7α* transcript isoforms detected by RT-PCR in four biological replicates. The *ACTIN2* gene was amplified as a loading control, and a negative control with no reverse transcription is shown (RT-). **d**, *CDCA7α* annotated isoforms^72^ confirmed by Sanger sequencing of RT-PCR products shown in (**c**), are highlighted (left). Primer positions are indicated by arrows, and translation start (AUG) and stop (*) codons are shown. Corresponding protein products are shown (right). **e**, Quantification of *CDCA7*α transcripts isoforms by RT-qPCR. Each data point is an individual primary transformant with mean values ± standard error shown in black. Groups not sharing the same letter are significantly different (two-sided Tukey’s HSD tests, *P* < 0.05). Expression levels are normalized to *ACTIN2*. From top to bottom, sample sizes for each group are 14, 8, 9, 6, 12.

To test the regulatory effect of the *CDCA7α* haplotypes on *CDCA7* expression, we cloned the *CDCA7α-alt*_*a*_ promoter and fused it to the *CDCA7α-ref* coding sequence to complement *cdca7α* and *cdca7α* *cdca7β-3* mutants (Supplementary Fig. 5a). We also generated constructs where the indels present in *CDCA7α-alt*_*a*_ and *CDCA7α-alt*_*b*_ were deleted. In primary transformants, transgene expression was higher with the *CDCA7α-alt*_*a*_ promoter, and this effect depended on the presence of a 26-bp indel but not a 4-bp indel (Fig. 5e). Importantly, *CDCA7α* expression was positively correlated with mCG levels at TEs (Fig. 5f) and inversely correlated with TE expression (Extended Data Fig. 9a), regardless of the mutant background. These findings demonstrate that the 26-bp indel in the *CDCA7α* promoter regulates its expression, which in turn controls DNA methylation levels in natural *A. thaliana* populations. The fact that higher expression of *CDCA7α* led to increased mCG levels further implies that in nature, *CDCA7α* is a limiting factor in promoting mCG and TE repression.

The construct without the 26-bp indel, despite producing *CDCA7α* transcript levels similar to those of the reference promoter (Fig. 5e), was associated with reduced mCG (Extended Data Fig. 9b), suggesting that steady-state *CDCA7α* transcript levels are not sufficient to predict mCG levels. There are six reported *CDCA7α* transcript isoforms^72^. Under our experimental conditions, we detected three: *CDCA7α.2*, *CDCA7α.3* and *CDCA7α.5* (Extended Data Fig. 9c), and their relative abundance varied across conditions (Supplementary Fig. 8). *CDCA7α.2*, the most abundant isoform (Extended Data Fig. 9c), encodes the canonical CDCA7α protein, while *CDCA7α.3* has no annotated protein product and *CDCA7α.5* produces a protein lacking the DDM1-interacting domain (Extended Data Fig. 9d). We found that both *CDCA7α.2* and *CDCA7α.3* were overexpressed in lines with the *CDCA7α-alt*_*a*_ promoter, and this overexpression required the 26-bp indel (Extended Data Fig. 9e), consistent with our quantification of all isoforms (Fig. 5e). However, deleting the 26-bp indel had no strong effect on the *CDCA7α.5* isoform relative to the intact *CDCA7α-alt*_*a*_ promoter (Extended Data Fig. 9e), indicating that this indel regulates isoform usage. *CDCA7α.5*, which lacks the DDM1-interacting domain but retains an intact zinc-finger, could compete with the canonical CDCA7α for binding to target loci, thereby antagonizing DDM1 activity. We hypothesize that an increased relative abundance of *CDCA7α.5* would contribute to the observed mCG reduction in transgenic lines with the *CDCA7α-alt*_*a*_ promoter lacking the 26-bp indel (Extended Data Fig. 9b).

In conclusion, the three *CDCA7α* haplotypes influence DNA methylation and TE repression through different mechanisms. The *CDCA7α-alt*_*a*_ and *CDCA7α-alt*_*b*_ haplotypes, with their unique promoter sequence, drive higher *CDCA7α* expression and corresponding mCG levels than the reference haplotype. Moreover, the 26-bp indel plays a critical role not only in regulating *CDCA7α* expression but also in controlling the balance of transcript isoforms, which has downstream effects on mCG levels. While no genetic difference between the *CDCA7α-alt*_*a*_ and *CDCA7α-alt*_*b*_ haplotypes was observed in the tested promoter region (Fig. 5d), there could be additional structural variants explaining their variation in expression (Fig. 5b).

### The *CDCA7α-ref* allele is derived and predominantly occurs in Europe

**Fig. 6:**
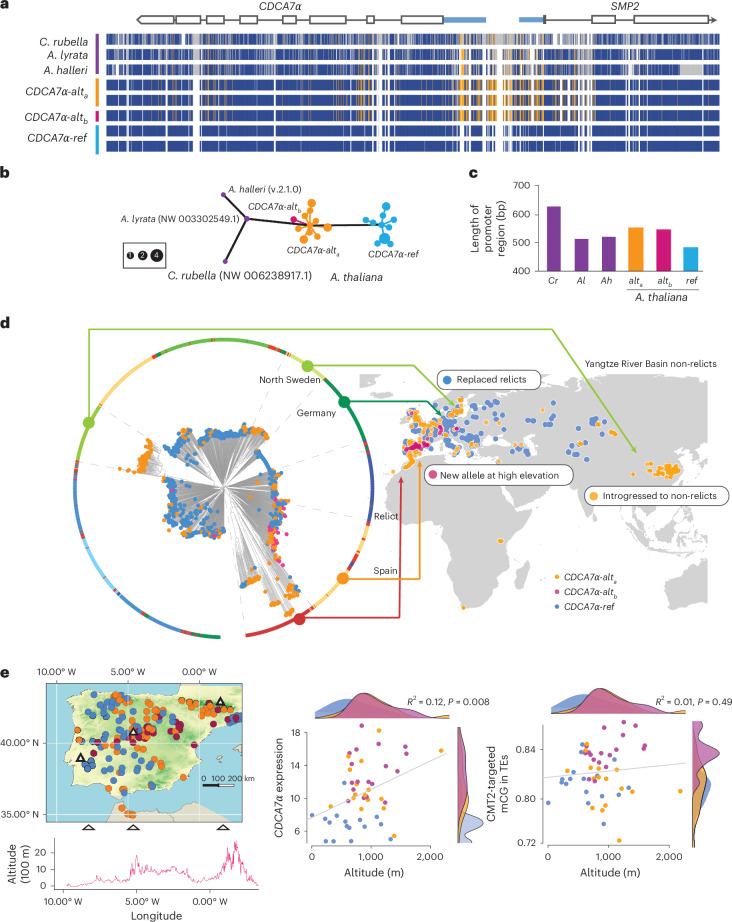
The evolutionary history of *CDCA7α* haplotypes. **a**, Alignment of *CDCA7α* regions in *A. thaliana* and sister species. Blue, orange and grey regions represent the reference, alternative and non-*A. thaliana* alleles, respectively. **b**, Haplotype network of a 4-kb region covering the *CDCA7α* and *SMP2* coding region. **c**, Variation in *CDCA7α* promoter length. **d**, Population structures of 1,323 natural lines with *CDCA7α* alleles and their geographic distribution. The phylogenetic tree is a neighbour-joining tree^75^ with populations (outer circle) and the genotypes of *CDCA7α* haplotypes (at the tips). The map shows an overview of allele distributions. **e**, Altitudinal cline of alleles and mCG variation. The local map of the Iberian Peninsula indicates the altitudes of summits with triangles, and scatter plots show associations between altitude and *CDCA7α* expression (left) and mCG in CMT2-targeted TEs (right), with the allele distributions as density plots. For scatter plots, all data were fitted to a linear regression model, and *P* values were calculated using a two-sided *F*-test. Source data

We investigated the history of the *CDCA7α* promoter region to gain insight into the evolution of the epigenome in *A. thaliana*. To understand which form of the *CDCA7α* promoter is ancestral, we compared the *CDCA7α* haplotypes with closely related species, including *A. lyrata*, *A. halleri* and *Capsella rubella*. These species clearly exhibited higher similarity to the *CDCA7α-alt*_*a*_ and *CDCA7α-alt*_*b*_ haplotypes than to *CDCA7α-ref* (Fig. 6a). The predicted haplotype network supported the conclusion that the *CDCA7α-alt*_*a*_ haplotype, causing higher mCG levels, is ancestral, while *CDCA7α-ref* is derived, and the promoter indels reflect deletions (Fig. 6b). Compared with *CDCA7α-alt*_*a*_, *CDCA7α-ref* has a 12.3% shorter promoter region (Fig. 6c). *CDCA7α-alt*_*b*_, which drives the highest mCG levels, accumulated specific SNPs not shared with related species, and the haplotype network showed that *CDCA7α-alt*_*b*_ was derived from *CDCA7α-alt*_*a*_ (Fig. 6b). The regulation of *CDCA7α* expression has thus undergone multiple changes, resulting in variation in mCG levels at TEs in heterochromatic regions.

The *CDCA7α-alt*_*a*_ haplotype was predominantly found in three regions: (1) northern Sweden, (2) the Iberian Peninsula and (3) eastern Asia, specifically the Yangtze River Basin population, which is the most southeastern edge of native *A. thaliana* habitats (Fig. 6d). Consistent with *CDCA7α-alt*_*a*_ being ancestral, its distribution overlaps with the ancestral-type populations named ‘relict’ observed in northern Sweden and the Iberian Peninsula^73,74^. Relicts have genomes that diverged from those of the current worldwide population, referred to as ‘non-relicts’, and are considered to have been dominant in post-glacial Eurasia^73,74^. Non-relicts comprising over 95% of current populations are probably modern populations that replaced the relicts as they spread to central and western Europe through human activity. Most of the lines carrying *CDCA7α-ref* were non-relicts (Fig. 6d). Although the Yangtze River Basin population is non-relict, expected to have diverged about 60,000 years ago^75^, *CDCA7α-alt*_*a*_ was fixed in this relatively new population (Fig. 6d). This is in agreement with studies suggesting that genomic introgression occurred from local relicts to the Yangtze River Basin population^75^, and the *CDCA7α* region appears to have been introgressed, as shown in our phylogenetic tree (Fig. 6d). Additionally, a prior selection scan using the composite likelihood ratio in Zou et al.^76^ indicated a signature of positive selection over a 15-kb region that includes *CDCA7α*, suggesting that this allele may be involved in local adaptation.

Unlike *CDCA7α-alt*_*a*_, *CDCA7α-alt*_*b*_ was predominantly found in the Spanish population, particularly in the central mountain region of the Iberian Peninsula (Fig. 6d,e). This mountainous area stretches from east to west, exhibiting diverse climates due to its wide range of altitudes, up to 3,000 m above sea level^77^. While *CDCA7α-alt*_*b*_ appears to have been derived from *CDCA7α-alt*_*a*_, non-relict Spanish populations carry *CDCA7α-alt*_*b*_ (Fig. 6d), suggesting that *CDCA7α-alt*_*b*_ probably emerged after *CDCA7α-alt*_*a*_ was introgressed into non-relicts. Across the Spanish population in the Iberian Peninsula, both *CDCA7α-alt*_*a*_ and *CDCA7α-alt*_*b*_ were distributed at higher altitudes than *CDCA7α-ref*, resulting in an altitudinal cline of mCG levels on TEs (Fig. 6e).

**Extended Data Fig. 10:**
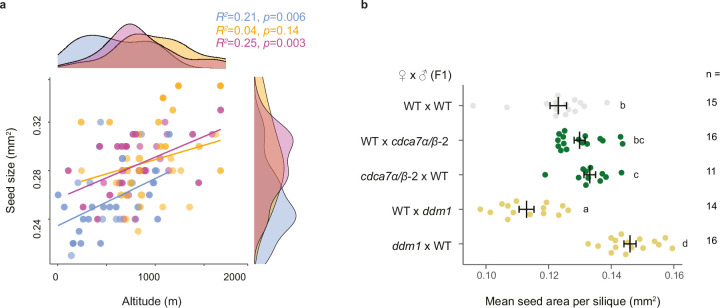
The effect of CDCA7α on seed size. **a**, A scatter plot shows the association between altitude and seed size, with *CDCA7*α allele distribution shown as density plots (reanalyzed data^77^). All data were fitted to a linear regression model, and *p*-values were calculated using a two-sided F-test. **b**, Dot plots show parent-of-origin effects of *cdca7α/β-2* and *ddm1* on seed size, in F1 crosses to WT. Each data point shows the average seed size per silique, with mean values ± standard error shown in black. Sample size for each group is indicated to the right. Groups not sharing the same letter are significantly different (two-sided Tukey’s HSD test, *P* < 0.05). Source data

If *CDCA7α* is directly involved in expansion to new environments, *CDCA7α*-mediated epigenetic changes must impact life-history traits that provide adaptive features. For example, flowering time and seed size are influenced by DNA methylation^78–80^ and contribute to reproductive success in challenging environments^81,82^. While *cdca7α* *cdca7β* mutants did not affect flowering time under laboratory conditions (Supplementary Fig. 9), a maternal effect was observed in seed size (Extended Data Fig. 10a,b). This differed from *ddm1*, which showed the expected parent-of-origin effect^78,79,83^. Although the larger seed size resulting from maternal *cdca7α* *cdca7β* in the F_1_ population did not follow the same direction as the increased seed size in lines carrying *CDCA7α-alt*_*a*_ or *CDCA7α-alt*_*b*_, this difference may be explained by interactions with the genetic background. Further studies will help elucidate the underlying molecular mechanisms. There were no a priori genes involved in seed size regulation within three kilobases of TEs targeted by *CDCA7α*, suggesting that mechanisms other than gene regulation lead to larger seed size, possibly as a consequence of genome-wide loss of mCG. Consistent with the mutant phenotype, seed size variation in the Spanish mountain population showed a clear altitudinal cline and was positively associated with the distribution of *CDCA7α* alleles along altitude (Extended Data Fig. 10a,b). While seed size is a complex trait controlled by multiple loci^81,84^, our results suggest mCG changes through *CDCA7α* alleles potentially contribute to it and may play a role in colonizing new environments.

## Discussion

The establishment and maintenance of the epigenome—particularly mCG, a DNA modification that is transgenerationally inherited—has been much debated^12,85,86^. Our study reveals molecular mechanisms that explain the epigenome variation observed in nature. We identified *CDCA7α* as a new *trans* regulator of mCG in plants and showed that its expression variation has strongly shaped the epigenome of natural populations of *A. thaliana*. The ancestral *CDCA7α* promoter allele, present in relict populations, drives higher *CDCA7α* expression than the derived alleles found in non-relict populations across western and central Europe, including the reference strain Col-0. Worldwide, these changes result in lower mCG levels and reduced TE repression in current populations compared with the ancestral form.

In addition to natural variation in *CDCA7α*, the retention of two *CDCA7* copies following whole-genome duplication (Extended Data Fig. 2) may have influenced mCG dynamics. In jawed vertebrates, *CDCA7* is also duplicated^44^, and the absence of one *CDCA7* paralogue in mice had no effect on TE silencing^87^, suggesting that *CDCA7* paralogues could have redundant functions in animals. In *A. thaliana*, *CDCA7α* has a stronger impact on mCG than *CDCA7β* (Fig. 2). This difference is probably not due to the divergence in their C-terminal domains, as this region was found to be dispensable for TE silencing (Fig. 4a,b). Instead, the stronger impact of *CDCA7α* probably results from its higher expression levels (Supplementary Fig. 2). The redundant roles of *CDCA7α* and *CDCA7β* indicate that *CDCA7* duplication may have increased CDCA7 protein dosage or enhanced robustness against genetic and environmental challenges^88^.

We provide evidence that a *trans* regulator, *CDCA7α*, causes changes in DNA methylation that, in turn, have the capacity to influence natural populations. Changes in the expression level of this limiting factor perturbed mCG levels on TEs and potentially affected seed size, an adaptive trait. DNA methylation could influence the adaptive capacity of the seed via its regulation of seed size^83,89^. Seed size in *A. thaliana* is primarily controlled by an interplay between the outer cell layers that protect the seed and the endosperm that protects and nourishes the embryo^90^. In the endosperm, many genes are expressed preferentially from a single parental allele^91,92^. This imprinted expression pattern results in part from the loss of DNA methylation in the endosperm progenitor cell. Imprinted genes that remain methylated in vegetative tissues but are expressed predominantly from the maternal allele in endosperm typically restrict endosperm growth and, consequently, limit seed size. Such genes are under selection^93–95^ and could relay the impact of DNA methylation on dormancy^96,97^. By influencing the trade-off between producing a few large seeds with higher seedling survival and producing many small, widely dispersed seeds^98^, imprinting helps determine the balance between fecundity and seed quality^91,92^.

Our results support the hypothesis that evolutionary changes in the dosage of epigenetic regulators have caused global changes in epigenetic profiles^86^ (Fig. 5). To date, we have identified several other *trans* regulators controlling mCH, including *CMT2*, *CMT3*, *MIRNA823* (which targets *CMT3* transcripts), *JMJ26* and RNA polymerase V^23,25,26,99^. To this list, we now add *CDCA7α* and *DDM1*, affecting mCG, which is directly inherited. As combinations of epigenetic mutants often exhibit epistasis^100–102^, these allelic interactions—spanning multiple pathways and all DNA methylation contexts—considerably elevate epigenetic diversity and have the potential to modify gene regulatory networks and TE silencing. Such genetically inherited epigenetic variation can become a target of natural selection^86^. Our study provides insights into epigenome variation historically observed in nature at the molecular level. Further studies, including analysis of genetic and epigenetic architecture in other species, will enhance our understanding of the complex ecological significance of the epigenome in the tree of life.

**Fig. 7:**
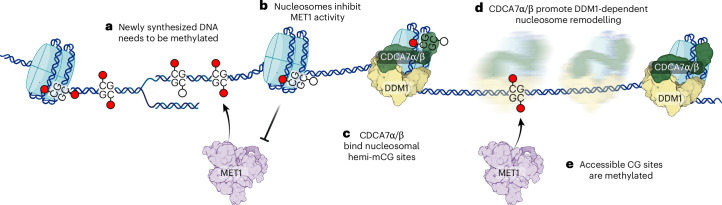
Proposed model of mCG maintenance by CDCA7α/β. **a**–**e**, DNA synthesis incorporates unmethylated cytosines (**a**), which are later methylated by DNA methyltransferases. Nucleosomes are rapidly re-assembled behind the replication fork, inhibiting the activity of the CG methyltransferase MET1 (**b**), while linker DNA is accessible to MET1 activity. The CDCA7α/β zinc-finger domain preferentially binds hemi-methylated CG sites bound to nucleosomes (**c**), facilitating DDM1 recruitment and/or activity. The CDCA7α/β–DDM1 complex remodels the nucleosome (**d**), leaving hemi-methylated sites accessible for methylation by MET1 (**e**). Figure created with BioRender.com.

How does CDCA7 promote mCG? Studies in *Xenopus* egg extracts and mouse embryonic stem cells showed that CDCA7 recruits HELLS (the DDM1 orthologue) to chromatin and activates its nucleosome remodelling activity^31,42,43,103^. Moreover, the zinc-finger of animal CDCA7 and *A. thaliana* CDCA7α bind hemi-methylated CG dinucleotides, which are formed during DNA replication^31,43,63^. Therefore, CDCA7 probably functions by recruiting DDM1/HELLS to hemi-methylated mCG sites^31^, and DDM1/HELLS would then remodel nucleosomes to provide methyltransferases with access to DNA^37,54^ on TEs and gene bodies (Fig. 7). This model is compatible with our observations: CDCA7α/β are required for mCG maintenance (Fig. 2), and they act in the DDM1 genetic pathway (Fig. 3), bind the DDM1 protein (Fig. 4) and maintain mCG at nucleosome-bound DNA (Fig. 3e). Together, this implies that this CDCA7 function is conserved in plants and metazoans.

CDCA7α/β act as facultative activators of DDM1, since they operate through DDM1, but their absence had a weaker impact on mCG than DDM1 loss (Fig. 2). This suggests that DDM1 may function on its own or with factors other than CDCA7α/β. DDM1 alone can remodel nucleosomes in vitro^32,33,104^. In contrast, HELLS requires CDCA7 for in vitro activity^42,105^, and animals have limited amounts of mCH^106^. Together with our observations that CDCA7α/β is specialized in mCG maintenance through DDM1 (Fig. 3), this suggests that CDCA7α/β would promote DDM1 remodelling only for mCG maintenance, implying that DDM1 maintains mCG and mCH through distinct mechanisms. DDM1’s intrinsic remodelling activity may have evolved specifically in plants to maintain mCH independently of CDCA7α/β.

Beyond mCG maintenance, DDM1/HELLS regulates histone variant exchange in plants and animals^32,48,51,107,108^, but the contribution of CDCA7 to this function had not been investigated. Our results indicate that CDCA7α/β are predominantly involved in mCG regulation, with minimal impact on other DDM1 functions (Figs. 2 and 3). This specificity aligns with the fact that *CDCA7* has been lost in most eukaryotes lacking DNA methylation, while *DDM1/HELLS* has been retained^44^. Accordingly, the absence of one CDCA7 paralogue affects mCG but not trimethylated H3 K9 in mice^87^. The comparatively mild reduction of heterochromatic marks other than mCG in *cdca7α* *cdca7β* suggests these are indirect effects, stemming from a primary and more stringent requirement for CDCA7 in DDM1-mediated mCG. This raises the question: how do CDCA7α/β selectively contribute to a single DDM1 function, given that DDM1-dependent heterochromatin marks generally co-occur in the genome? We propose a model where, after DNA synthesis, histone variant incorporation by DDM1 occurs first and in a CDCA7α/β-independent manner. Subsequently, CDCA7α/β recruit DDM1 to hemi-mCG sites to stimulate nucleosome remodelling and facilitate mCG deposition (Fig. 7). This model is consistent with the kinetics of nucleosome assembly being about one order of magnitude faster than mCG deposition after DNA replication^109,110^ and is further supported by observations that CDCA7 preferentially binds hemi-mCG sites located in nucleosomal rather than linker DNA^31^. Mechanistically, the contact of CDCA7α/β with DDM1’s H2A.W-binding module (Fig. 4) may outcompete H2A.W and promote DDM1’s function in nucleosome remodelling at the expense of histone variant incorporation, possibly through conformational changes. Further studies will be essential to pinpoint the specific timing and coordination of CDCA7 in DDM1/HELLS functions. A recent study independently reported a similar, predominant role for CDCA7α/β in regulating mCG, corroborating our conclusions^111^.

## Methods

Additional information is available in the Supplementary Methods.

### Plant material

Seeds sown on soil were stratified at 4 °C for two days in the dark and grown at 21 °C under long-day conditions (16 h light and 8 h dark) in a climatic chamber.

#### Mutants from stock centres and previous studies

The *cdca7α-1* (SALK_100123C), *cdca7α-2* (SALKseq_124190.3) and *ddm1-2* (ref. ^112^) mutant plants and control plants were in the Col-0 genetic background. To reset the effect of the transgenerational aggravation of the *ddm1-2* mutant phenotype^47^, we backcrossed a *ddm1-2* homozygous mutant six times to Col-0, keeping *ddm1-2* heterozygous throughout, and subsequently isolated homozygous *ddm1-2* mutants, which were considered first generation for this study.

#### CRISPR–Cas9 mutagenesis

CRISPR mutants were generated by transforming Col-0, *ddm1-2* second-generation mutants or *cdca7α-2* mutants. To mutate the *CDCA7α/β* genes, we used a CRISPR–Cas9 system based on an intronized *Cas9* gene controlled by the ubiquitous RPS5 promoter^113^. Guide RNAs controlled by a U6 promoter were cloned into pICH47751 or pICH47761, and binary plasmids were assembled using Golden Gate assembly with one or two guide RNAs, the intronized Cas9 gene, a linker for plasmid assembly and a seed coat fluorescent reporter using mVenus^114^ or a Basta resistance gene. Transgenic lines were generated with *Agrobacterium tumefaciens* GV3101 via floral dipping. T_1_ transformed seeds were selected using Basta resistance or seed coat fluorescence. Mutations were identified via PCR and Sanger sequencing. We isolated T_2_ seeds devoid of the transgene and selected mutations. We obtained stable homozygous mutant lines in T_2_ or T_3_ and used their progeny for this study. Details about the primers, guide RNAs and CRISPR mutations used in this study can be found in Supplementary Tables 2 and 3.

#### Complementation

*CDCA7α* and *CDCA7β* transgenes were cloned from genomic DNA to include endogenous regulatory sequences in promoters, 5′ untranslated regions (UTRs) and introns. The primers are listed in Supplementary Table 3. We used the Greengate cloning system^115^ to assemble constructs into a pGGSun backbone^116^. Promoters included 5′ UTRs and were cloned from the first base before the ATG start codon to 792 bp upstream for *CDCA7α* and 1,349 bp upstream for *CDCA7β*. The alternative *CDCA7α-alt*_*a*_ promoter was cloned from *A. thaliana* accession 6966 using the same primers as for the *CDCA7α-ref* promoter, resulting in an 831-bp fragment. Coding sequences contained all introns and exons, except the 3′ UTR and the start and stop codon, according to the tag position. Endogenous *CDCA7β* BsaI sites were removed via mutagenesis, introducing silent mutations (GTC to GTT and GGT to GGA). N-terminal tags had a start codon with 3xcMyc fused with mTurquoise2; C-terminal tags had mTurquoise2 fused with 3xcMyc and a stop codon. All constructs were cloned with the terminator of the UBQ10 gene (628 bp) and an mVenus seed coat reporter gene. Deletions and point mutations were introduced in constructs using in vivo cloning^117^. Transgenic lines were generated with *A. tumefaciens* GV3101 via floral dipping, and transformed seeds were selected using seed coat fluorescence^114^.

### Sample collection for epigenomics

Plants were grown following a randomized pot pattern. For methylomes and transcriptomes of *cdca7* and *ddm1* *cdca7* mutants (Figs. 2 and 3), we pooled rosette leaves from three 31-day-old plants (two rosette leaves each), ground the tissues in liquid nitrogen and split the ground tissues for DNA and RNA extraction. For ChIP–seq, we pooled at least five plants using five rosette leaves each, up to 1.5 g per sample. For methylomes and transcriptomes of complemented *cdca7* mutants (Figs. 4 and 5), we used 28-day-old plants: one rosette leaf (2–3 cm long) was flash-frozen first for RNA extraction, and then all remaining rosette leaves except cotyledons and leaves 1 and 2 were collected for DNA extraction. All generated libraries are listed in Supplementary Table 4, with the number of inbreeding generations for each mutant.

### Tagmentation-based whole genome bisulfite sequencing

#### Library construction and sequencing

Genomic DNA was extracted from rosette leaves with the DNeasy Plant Pro kit (Qiagen, reference no. 69206), following the manufacturer’s instructions, using one to five biological replicates per genotype (Supplementary Table 4). Illumina libraries of sodium-bisulfite-converted DNA were prepared with a tagmentation-based approach^118^ with modifications^119^. Briefly, we tagmented 50 ng of DNA with Tn5 (Molecular Biology Services, IMP). Tagmented DNA was purified with the Zymo DNA Clean and Concentrator kit (Zymo Research) instead of SPRI beads. After oligonucleotide replacement, gap repair and purification, we used the entirety of the eluted DNA for bisulfite conversion, which was performed with the EZ DNA Methylation-Gold Kit from Zymo (reference no. D5006). The rest of the protocol was performed as recommended^118^. Directional libraries were amplified with Nextera-compatible unique dual indices and sequenced on a NovaSeq 6000 instrument using an S4 flow cell to generate paired-end 150-bp reads with around 25 million reads per sample.

#### Tagmentation-based whole genome bisulfite sequencing analysis

Libraries for *cdca7α* *cdca7β* and *ddm1* mutants (Figs. 2 and 3) were randomly subsampled to 30 million reads to improve depth homogeneity, since there were up to twofold more reads in some samples. The data were analysed with the nfcore methylseq pipeline v.2.3.0 (https://nf-co.re/methylseq)^120^ with Nextflow (v.22.10.7). Cytosine positions covered by fewer than three reads (meth_cutoff, 3) were excluded. Tagmentation creates gaps in the double-stranded DNA, which are later filled by unmethylated cytosines during gap repair. These nucleotides were excluded from the analysis by trimming reads with the ‘clip’ and ‘ignore’ options, using M-bias plots to ensure the removal of unmethylated fragment ends. The parameters were as follows: meth_cutoff, 4; comprehensive; cytosine_report; clip_r1, 14; three_prime_clip_r1, 4; three_prime_clip_r2, 2; ignore_3prime_r2, 17. Note that the unmethylated cytosines are incorporated at the 5′ end of read 2, while the options mentioned above suggest we ignored the 3′ end of read 2 instead, but methylseq v.2.3.0 had an issue that made it clip the wrong end of read 2 (see https://github.com/nf-core/methylseq/issues/299). Using M-bias plots, we noticed a 5′ bias on read 1 that resembled the Tn5 sequence bias^121^, and we removed it by clipping 14 nucleotides at the 5′ end of read 1. We also excluded additional small methylation biases at the 3′ ends of read 1 and read 2.

To average biological replicates, we computed the mean methylation level for individual cytosine positions, including all replicates that met the minimum coverage threshold. Positions covered in only one replicate were retained to maximize data inclusion. To compute the average methylation values over annotation sets or 100-bp bins, we used the bigWigAverageOverBed script from UCSC tools (https://github.com/ucscGenomeBrowser/kent-core/tree/master). Only TEs covered in all samples from a common batch were retained. To analyse DNA methylation at TEs, we excluded TEs unmethylated in the WT, using a threshold of 5% methylation for mCG, 3% for mCHG and 2% for mCHH, after which the sample sizes were 18,052, 16,739 and 18,037, respectively. To calculate average values in bins (metaplots), annotations were scaled to an arbitrary size of 1,000 bp, and the methylation value was calculated across annotation length by 25-bp bins and up to 1,000 bp upstream and downstream. This was done with the computeMatrix scale-regions binSize 25 and plotProfile functions from deepTools v.3.3.1. For metaplots at TEs, elements below 200 bp were excluded to minimize scaling artefacts. Gene-body methylation analysis was restricted to protein-coding genes that had more than 10% mCG, less than 10% mCHG and 10% mCHH in the WT and were covered in all samples (*n* = 9,938). To analyse 5mC changes in 100-bp bins, we segmented the genome into non-overlapping 100-bp bins and counted the number of each methylation context per bin using a custom Python script. The average 5mC per bin was calculated with bigWigAverageOverBed, and only bins that were covered in all samples were retained for further analysis. To compute Spearman correlations, we kept bins that had at least 10% mCG, 6% mCHG and 5% mCHH in the WT. To analyse mCG at individual reads (single-molecule level), we excluded reads with four CG sites or less to minimize noise and reads with 0% mCG. The positions of well-positioned nucleosomes in the WT and *ddm1* were extracted from a previous study^54^. To isolate well-positioned nucleosomes at heterochromatic TEs, we reproduced the approach from Lyons and Zilberman^54^, in which TEs in the highest two H3K9me2 quintiles with mCG equal to or above 5% are considered heterochromatic. Metaplots were generated with deepTools computeMatrix reference-point binSize 10. Methylation load integrates methylation levels (the average methylation frequencies of individual cytosines over a TE) with cytosine context density: it is the sum of all individual methylation values in a genomic window, further divided by the size in kilobases, yielding the average number of fully methylated cytosines per kilobase.

### 3′-tag sequencing of mRNAs

#### Library construction and sequencing

Total RNA was extracted using the RNA isolation kit provided by the VBC core facilities. The kit uses a lysis step based on guanidine thiocyanate adapted from a previous study^122^ and carboxylate-modified Sera-Mag Speed beads and was applied using the KingFisher instrument (Thermo).

Libraries were prepared in 96-well plates using a custom protocol described previously^123^. The oligonucleotide d(T) primers used for reverse transcription contained 8-bp unique molecular identifiers (UMIs), 7-bp barcodes for multiplexing and a Truseq adapter. After reverse transcription, samples were pooled, and RNA–cDNA duplexes were tagmented with Tn5. Using primers matching the Truseq and Tn5 adapters, the 3′ ends of mRNA transcripts were subsequently amplified via PCR, while P5 and P7 sequences and plate-specific dual indices were added for Illumina sequencing.

We started from 80 ng of total RNA in 4 µl, adding 1 µl of 0.2 µM primers for reverse transcription. RNAs were denatured for 3 min at 72 °C and kept on ice. We mixed 1 µl of the SMARTScribe reverse transcriptase (Takara) in a final volume of 10 µl with 1× SMARTScribe buffer, 1.75 mM dNTPs, 2 mM DTT and 3 mM MnCl_2_ for 1 h at 42 °C followed by inactivation for 15 min at 70 °C. All samples were pooled together and purified using 1.2× volume of SPRI beads (Molecular Biology Services, IMP). After mixing and 2 min at room temperature, the beads were collected on a magnet, washed two times with 70% ethanol, dried and eluted in 60 µl of 10 mM Tris-HCl pH 8.0. To assemble transposomes, we mixed Tn5 (0.38 mg ml^−1^) with 18 µM of annealed adapters, 50% glycerol and 1× Tn5 loading buffer (6× buffer: 300 mM Hepes KOH pH 7.2, 600 mM NaCl, 0.5 mM EDTA, 6 mM DTT, 0.6% Triton-X-100) and incubated the mixture for 1 h at 37 °C. For tagmentation, 16 µl of pooled RNA–cDNA was mixed with 16 µl of transposome and 32 µl of 2× tagmentation buffer (20 mM Tris (hydroxymethyl)aminomethane, 10 mM MgCl_2_, adjusted to pH 7.6 and 20% (vol/vol) dimethylformamide). After 8 min at 55 °C, we added 16 µl of 0.2% SDS and incubated it for 5 min at room temperature. Nucleic acids were purified with 2× SPRI bead volume as indicated above with a 48-µl elution. For PCR, we pre-incubated the KAPA HiFi HotStart 2X ReadyMix at 95 °C for 90 s to activate the polymerase and amplified the libraries in a final volume of 100 µl using 1 µM primers and 46 µl of purified cDNA–RNA duplexes with the following program: 72 °C for 3 min; 95 °C for 30 s; 12 cycles of 95 °C for 20 s, 65 °C for 30 s and 72 °C for 30 s; and 72 °C for 5 min. The libraries were purified with 0.8× volume of SPRI beads as indicated above and eluted in 62 µl. After controlling for appropriate fragment size distribution on a 5200 Fragment Analyzer System (Agilent), the libraries were sequenced as paired-end 150-bp reads on a NovaSeq S4 (Figs. 2 and 3) or a NovaseqX 10B (Figs. 4 and 5) flow cell.

#### Transcriptome mapping, differential expression and analysis

Reads were demultiplexed, trimmed, collapsed to remove duplicates, mapped and quantified. The code and documentation are available at https://github.com/pierre-bourguet/3-tag-rna-seq, but we provide the most important details below. Read 1, which starts directly in the transcript at the site of Tn5 cutting, was used for mapping. Read 2, which contains the sample-specific index, the UMI, the polyA tail and eventually the 3′ end of the transcript, was used for demultiplexing and UMI extraction. Specifically, we used the first seven bases of read 2 to identify indices specific to each well in the plate, and the next eight bases to extract UMIs. For read 1, the 3′ end was trimmed for polyA and adapter contamination, and reads shorter than 50 bp were discarded. UMIs were prepended to read 1, and these chimeric reads were used to collapse duplicates into a single read with clumpify (bbmap v.38.26)^124^, with no mismatches allowed in the UMI and two mismatches allowed in the transcript sequence. Mapping was performed with STAR v.2.7.11a (ref. ^125^), and mapped reads were counted with Salmon v.1.10.1 in alignment mode^126^ in sense and antisense. We used the TAIR10 genome and AtRTD3 transcriptome ref. ^72^. Because the AtRTD3 GFF and fasta files were sometimes discordant, we regenerated a fasta file from a fixed GFF file using AGAT v.1.0.0 (ref. ^127^). We observed that the 3′ ends of transcripts were often located outside of TE gene annotations, probably because they do not include 3′ UTRs, but within TAIR10 TE annotations. For this reason, and to avoid overlapping annotations, we removed ‘transposable element gene’ annotations from AtRTD3 and added the 31,189 TE annotations from TAIR10 instead.

We used DESeq2 v.1.42.1 (ref. ^128^) for a quantitative analysis of read counts. For a given feature, counts in the sense or antisense orientation were considered as independent features, meaning that a feature can be misregulated both in sense and in antisense. We ran the DESeq2 model in single-strand mode, removing features that did not have at least ten reads in a minimum of three samples. Features were considered differentially expressed at a false discovery rate of 10% (adjusted *P* ≤ 0.1) and an absolute log_2_ fold change relative to the WT equal or greater than 1. To avoid confounding effects of overlapping annotations, we removed TEs misregulated in sense or antisense if they overlapped (1 bp or more) a protein-coding gene in the same or reverse orientation, respectively. We used DESeq2 to normalize counts with the median ratio method (library size normalization) and to apply a variance-stabilizing transformation, which is on a log scale.

To quantify transgene expression, the transgene sequence and annotations were added as an additional chromosome in the fasta and GTF files. The C-terminal *mTurq2*::*3xcMyc* tag allowed us to distinguish transgenic reads from endogenous *CDCA7α* reads. For Fig. 5e, a two-way analysis of variance showed a significant effect of the promoter haplotype (*P* = 5 × 10^−6^) on transgene expression levels, but no effect of the genetic background (*P* = 0.46) and no interaction (*P* = 0.48); therefore, we merged the data from the two different backgrounds.

To analyse *cdca7α* *cdca7β* and *ddm1* upregulated TEs (Fig. 3f–g, Extended Data Fig. 7 and Supplementary Fig. 4), we merged TEs upregulated in both sense and antisense (*n* = 115 out of 1,081 TEs upregulated in *ddm1*). The locations of centromeres were determined using cenH3 ChIP data^129^, by selecting the genomic window with the highest cenH3 enrichment for each chromosome (TAIR10 coordinates: chromosome 1, 15081947–15086107; chromosome 2, 3605658–3627529; chromosome 3, 13587594–13592597; chromosome 4, 3950478–3956032; chromosome 5, 11701785–11736471). Pericentromeric heterochromatin was previously defined on the basis of H3K9me2 signal^130^. To determine the TE families most divergent between cluster 1 and cluster 3, we first calculated the frequency of each TE family within each cluster as a percentage of total TEs in that cluster. We then applied a minimum threshold filter, retaining only families with ≥3% frequency in at least one cluster to focus on sufficiently abundant families for robust statistical comparison. For each qualifying TE family, we computed fold-change ratios between clusters 1 and 3 using the formula ratio = max(cluster_1%, cluster_3%)/max(min(cluster_1%, cluster_3%), 0.3), where a pseudocount of 0.3 was applied to prevent division by zero for families with 0% frequency in one cluster. Fisher’s exact test was performed comparing family frequency in one cluster versus other families in other clusters.

### Reverse transcription-quantitative PCR

We used 21-day-old and 27-day-old rosette leaves to perform the experiments shown in Extended Data Fig. 3g and Supplementary Fig. 5b, respectively. RNA extraction, transcript quantification and normalization were performed as previously reported^131^, except that reactions were run on a Roche LightCycler 96 system with the following program: 10 min at 45 °C, 2 min at 95 °C and 40 cycles of 5 s at 95 °C, 10 s at 60 °C and 5 s at 72 °C. The primers are listed in Supplementary Table 3.

### GWAS

GWAS were conducted using LIMIX^132^ v.3.0.4 with full genome SNPs from the 1001 Genomes Project (10,709,949 SNPs for mCG levels and 3,686,507 SNPs for gene expression) and the following linear mixed model:

1 $${\bf{Y}}=\alpha {\bf{L}}+\beta {\bf{X}}+g+e$$

2 $${\rm{v}}{\rm{a}}{\rm{r}}({\bf{Y}})={\sigma }_{g}^{2}K+{\sigma }_{e}^{2}I$$

where **Y** is the vector of a phenotype, and fixed terms **L** and **X** are *n* × 1 vectors corresponding to a cofactor and a genotype to be tested (SNP) with the parameters *α* and *β*, respectively. $g\sim {\rm{N}}(0,{\sigma }_{g}^{2}K)$ and $e\sim {\rm{N}}(0,{\sigma }_{e}^{2}I)$ are random terms, including the identity-by-state kinship *K* matrix representing the genetic relatedness^133,134^ and the residuals, where *I* is the identity matrix, respectively. *σ*_*g*_ and *σ*_*e*_ represent the parameters for genetic and environmental variance, respectively. The vectors **Y**, **L** and **X** were *z*-scored. Models without a cofactor take *α* = 0. SNPs with MAF ≥ 5% were used for association studies. Bonferroni correction was applied for multiple-testing correction after excluding all SNPs with MAF < 5%.

### Mediation analysis and heritability

The fraction of genetic effects on mCG variation mediated by *CDCA7α* expression was estimated using mediation analysis with a previously published program^71^. Two alleles, *CDCA7α-alt*_*a*_ and *CDCA7α-alt*_*b*_, were fitted into one model together as a single genotype vector: *CDCA7α-ref*, 0; *CDCA7α-alt*_*a*_, 1; and *CDCA7α-alt*_*b*_, 2.

### Local genetic structure

#### Genome alignment of the *CDCA7α* region and haplotype network construction

Fully assembled genome sequences of 27 *A. thaliana* lines^135^, *A. lyrata* v.1.0, *A. halleri* v.2.1.0 and *Capsella rubella* v.1.0 (ref. ^136^) were used for the alignments of the diverged *CDCA7α* promoter region. The numbers of *A. thaliana CDCA7-ref*, *CDCA7α-alt*_*a*_ and *CDCA7α-alt*_*b*_ lines were 14, 9 and 1, respectively*. A. lyrata* and *C. rubella* genomes were downloaded from NCBI, and the *A. halleri* genome was downloaded from Phytozome (https://phytozome-next.jgi.doe.gov/). We extracted a 4-kb region covering the *CDCA7α* and *SMP2* coding sequences on the basis of homology (chromosome 4 positions 17484000–17488000 in the *A. thaliana* Col-0 genome, TAIR10) and made the alignment using CLC Sequence Viewer v.8 with a gap open cost of 10.0 and a gap extension cost of 1.0. After removing all gaps, the alignment was applied to haplotype network construction via minimum spanning network^137^ implemented in popart^138^.

The length of the intergenic region between *CDCA7α* and *SMP2* was estimated as the distance of 1 bp outside the start codons of *CDCA7α.1* and *SMP2.1* (chromosome 4 positions 17486198–17486678; 481 bp in Col-0).

#### Local genetic structure of *DDM1*

The local genetic structure around the DDM1 locus was analysed using principal component analysis according to a previous study^139^. Using the prcomp function in R, we analysed 475 SNPs from 1,135 lines in the 1001 Genomes Project^74^, covering the region from chromosome 5 position 26649000 to position 26658200. Clusters represented by PC1 and PC2 were selected as components indicating the major haplotypes.

### Population structure and geographic distribution

We used the population structure information as genetic groups for 1,135 lines from the 1001 Genomes dataset. The information for an additional 188 lines, including Tanzania and the Yangtze River populations, was extracted from a previous study^75^. The pairwise genetic distance matrix calculated in ref. ^75^ was used to construct the neighbour-joining tree.

Latitude and longitude coordinates for each line were collected from the previous studies^74,76^, and altitude information was estimated using the R package elevatr (https://github.com/USEPA/elevatr).

Local maps (northern Sweden, the Iberian Peninsula and the Yangtze River) and the elevation profile view were generated using QGIS v.3.38.2 (https://www.qgis.org/).

### Public data analysis

#### Molecular phenotypes in natural populations

DNA methylation data from the *Arabidopsis* accessions used in this study were previously described^23,26^. Briefly, bisulfite sequencing data for 774 lines in leaf tissue grown under ambient temperature were used for this analysis. Reads were mapped on each pseudogenome, using a Methylpy pipeline (https://github.com/yupenghe/methylpy). mCG levels were calculated as weighted methylation levels^140^ for individual TEs. We used TAIR10 annotation to define TE regions, and 6,378 TEs having mapped reads in the region in all lines were used for all analyses. CMT2 and RdDM-targeted TEs were defined on the basis of differential methylation level (>0.1) between the WT and *drm1* *drm2* or *cmt2* in Col-0 (ref. ^60^).

Expression data for 461 lines, collected in the 1001 Genomes Project under the same conditions as the DNA methylation data, were used in this study^23,70^. As described in ref. ^70^, raw RNA-seq data were aligned to the TAIR10 genome using STAR v.2.9.6 (ref. ^125^), and gene expression levels were quantified using featurecounts^141^.

#### Seed size phenotypes in natural populations

Seed size and altitude information in the Spanish local population were collected from ref. ^77^. After extracting lines sequenced in the 1001 Genomes Project, we analysed only the genetically Spanish group to exclude the global population structure.

#### *CDCA7α* and *CDCA7β* expression profiles

To analyse the transcript levels of *CDCA7α* and *CDCA7β* across tissues and developmental stages^142^, we kept only tissues from the original dataset^143^ and from ref. ^142^, to limit overrepresentation of embryonic samples, and filtered out senescent tissues.

To analyse *CDCA7α* transcript variants, we aligned Iso-Seq reads to the TAIR10 reference genome with minimap2 v.2.24 using splice-aware long-read parameters (ax splice; uf; secondary, no)^144^. The resulting SAM files were converted to coordinate-sorted BAM files and indexed with samtools v.1.18 (ref. ^145^).

#### Epigenomic datasets

We used previously published datasets for histone H1 ChIP–seq^146^, pericentromeric heterochromatin boundaries^130^, cenH3 ChIP–seq^129^, WT and *ddm1* MNase-seq data, and well-positioned nucleosomes^54^.

### Data processing, plots and statistical analysis

All analyses were performed using R statistical software^147^ (v.4.3.2; https://www.R-project.org/) with the following packages: tidyverse v.2.0.0, ggplot2 v.3.5.1, patchwork v.1.2.0, agricolae v.1.3-7, ggbeeswarm v.0.7.2, MASS v.7.3-60, FSA v.0.10.0 (https://patchwork.data-imaginist.com, https://cran.r-project.org/package=agricolae, https://cran.r-project.org/package=ggbeeswarm, https://cran.r-project.org/package=MASS and https://cran.r-project.org/package=FAS)^148–154^. Two-sided Tukey’s HSD tests were performed using one-way analysis of variance with the aov function, followed by the TukeyHSD function. Other tests were performed with the functions dunnTest from FSA, fisher.test and chisq.test. *P* values were adjusted with p.adjust. The ComplexHeatmap package^155^ was used for heat maps and *k*-mean clustering, using the consensus clustering from 1,000 repeats. Some of the code and description in this study were generated with the assistance of large-language models.

To quantify the effect size relative to *ddm1*, we normalized mutant levels by WT levels (using average 5mC or ChIP levels) for each TE, and this difference was divided by the *ddm1*–WT difference to obtain effect size relative to *ddm1*. Median effect sizes across all TEs were reported to minimize the influence of outliers caused by TEs with low mutant-to-WT differences.

### Reporting summary

Further information on research design is available in the Nature Portfolio Reporting Summary linked to this article.

## Supplementary information

Supplementary Information Supplementary Figs. 1–9, Tables 1–3, Methods and references.

Reporting Summary

Supplementary Tables 4–6 Next-generation sequencing libraries generated in this study and genomes used for phylogenetic analysis.

## Source data

Source Data Fig. 1 Statistical source data.

Source Data Fig. 3 Statistical source data.

Source Data Fig. 4 Unprocessed western blots.

Source Data Fig. 5 Statistical source data.

Source Data Fig. 6 Statistical source data.

Source Data Extended Data Fig. 5 Statistical source data.

Source Data Extended Data Fig. 7 Statistical source data.

Source Data Extended Data Fig. 8 Unprocessed western blots.

Source Data Extended Data Fig. 10 Unprocessed gels.

Source Data Extended Data Fig. 10 Statistical source data.

## Data Availability

The data described in this publication have been deposited in NCBI’s Gene Expression Omnibus and are accessible through GEO Series accession numbers GSE284119 (tagmentation-based whole genome bisulfite sequencing), GSE283989 (whole genome bisulfite sequencing), GSE283987 (ChIP–seq) and GSE284067 (3′-tag mRNA-seq). Newly generated materials are available upon request. Source data are provided with this paper.

## Code availability

The custom code written to analyse 3′-tag mRNA-seq data is available via GitHub at https://github.com/pierre-bourguet/3-tag-rna-seq.
