# Supplementary material for: Major alleles of CDCA7 shape CG methylation in Arabidopsis thaliana
Source: Nat Plants. 2025 Nov 7;11(12):2511–30. doi: 10.1038/s41477-025-02148-w (PMC12711577; doi:10.1038/s41477-025-02148-w)
Supplement: Supplementary file 1 — Supplementary Figs. 1–9, Tables 1–3, Methods and references. [file 41477_2025_2148_MOESM1_ESM.pdf]

nature plants

Supplementary information https://doi.org/10.1038/s41477-025-02148-w

Major alleles of *CDCA7* shape CG methylation in *Arabidopsis thaliana*

In the format provided by the authors and unedited

This supplementary information file includes:

- Supplementary Figure 1. Substitutions in the DDM1 alternative allele affect an aminoacid of unknown function.
- Supplementary Figure 2. CDCA7 expression in Arabidopsis tissues.
- Supplementary Figure 3. DNA methylation periodicity around well-positioned nucleosomes.
- Supplementary Figure 4. Characterization of TEs upregulated in cdca7α/β and ddm1.
- Supplementary Figure 5. Complementation strategy of cdca7α/β mutants.
- Supplementary Figure 6. Physical interaction between CDCA7α and DDM1.
- Supplementary Figure 7. Protein sequences of CDCA7α from A. thaliana haplotypes and other Brassicaceae.
- Supplementary Figure 8. CDCA7α transcript variants detected by Iso-seq in different conditions.
- Supplementary Figure 9. Flowering time of cdca7α/ß null mutants.

- Supplementary Table 1. GWAS peaks
- Supplementary Table 2. Origin and description of CDCA7 mutants
- Supplementary Table 3. List of primers used in this study

- Supplementary Methods.
- Supplementary References.

Other supplementary materials not included in this file:

- Extended Data Figures 1 to 10
- Supplementary Tables 4 to 6

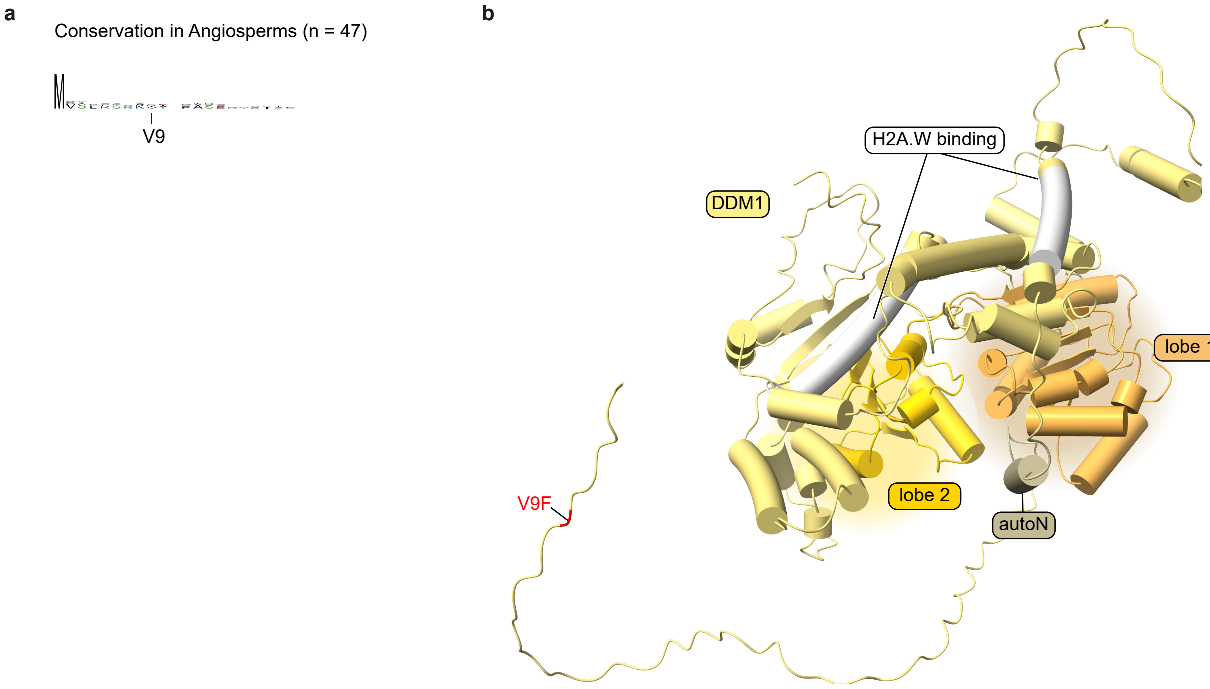

Supplementary Figure 1. Substitutions in the DDM1 alternative allele affect an aminoacid of unknown function.

a, DDM1 consensus sequence among Angiosperms (n = 47 species), showing the position of the amino acid substituted in the *DDM1* alternative allele. **b**, Predicted DDM1 structure, highlighting the amino acid substituted in the *DDM1* alternative allele (red) and the different DDM1 domains. The position of the autoinhibitory coiled coil domain (autoN) and H2A.W binding domains have been reported previously[1,2].

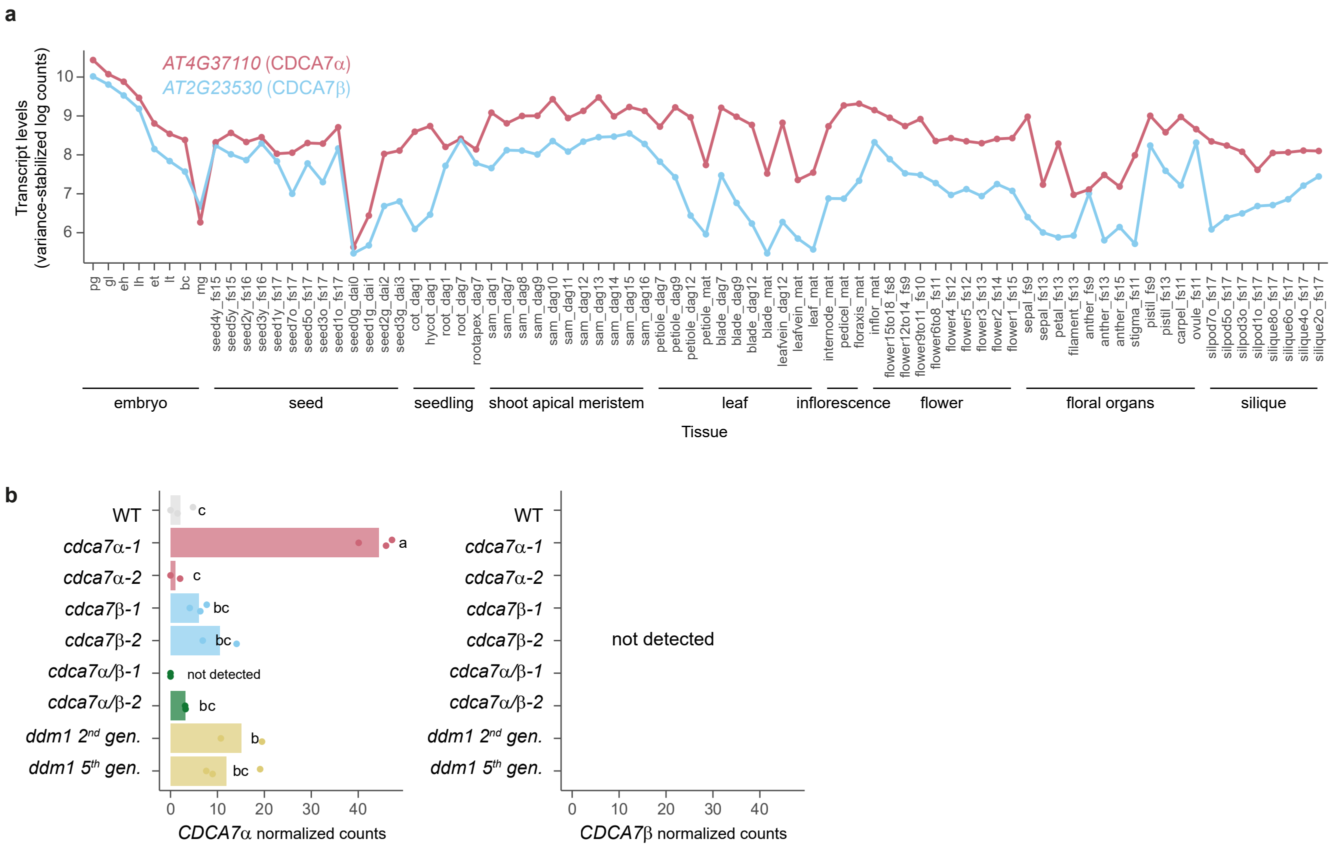

Supplementary Figure 2. CDCA7 expression in Arabidopsis tissues.

a, Transcript levels of *AT4G37110* and *AT2G23530* in transcriptome datasets from various developmental stages, shown as variance-stabilized log counts, from published data[3]. **b**, Transcript levels of *CDCA7α* and *CDCA7β*. Counts were normalized to library size with the median ratio method. Lower-case letters indicate significant differences as determined using two-sided Tukey's HSD test ($P < 0.05$).

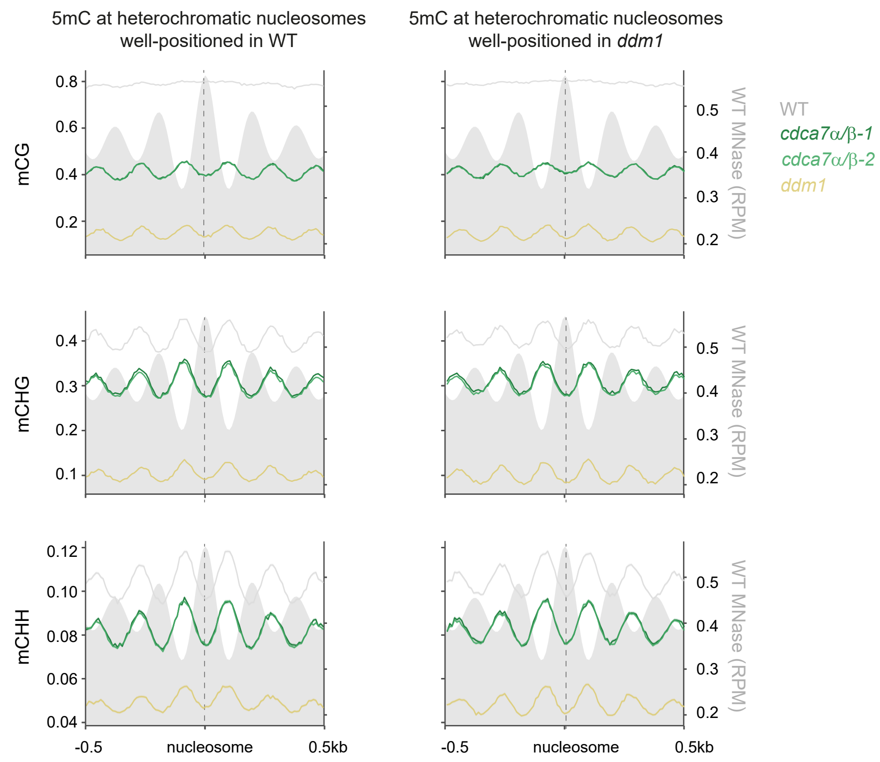

Supplementary Figure 3. DNA methylation periodicity around well-positioned nucleosomes.

Metaplots showing 5mC levels around well-positioned nucleosomes at heterochromatic TEs in WT (n = 22,515 nucleosomes) and *ddm1* (n = 21,844 nucleosomes). MNase-seq and nucleosome positions are publicly available[4].

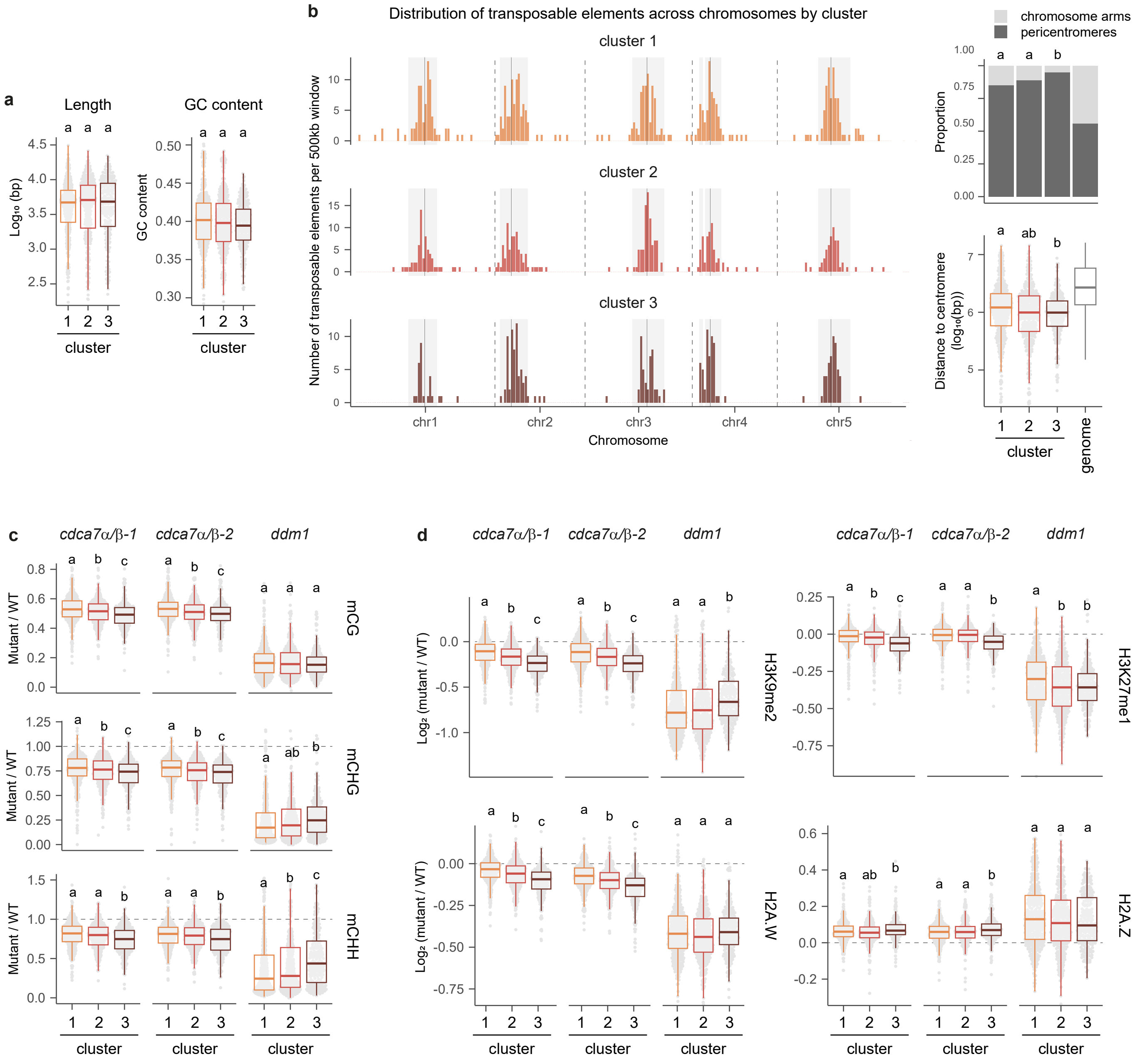

Supplementary Figure 4. Characterization of TEs upregulated in *cdca7α/β* and *ddm1.*

a, Length and GC content of *ddm1*-upregulated TEs (n = 966), clustered in **Fig. 3f**. Statistical groups were determined by two-tailed Dunn tests with Benjamini-Hochberg correction (*P* < 0.05), denoted by lowercase letters. **b**, Distribution of *ddm1*-upregulated TEs across chromosomes. Pericentromeric heterochromatin[5] is indicated by grey rectangles, and centromeres[6] are denoted by a black vertical line. (top right) Proportion of pericentromeric TEs in each cluster, compared with a Chi-square test using Benjamini-Hochberg correction (P < 0.05). (bottom right) Distance of TEs to centromeres. Log-transformed values were used to test differences between groups with two-tailed Dunn tests using Benjamini-Hochberg correction (P < 0.05), denoted by lowercase letters. **c**, 5mC and **d**, histone mark and variant changes in the indicated mutants at *ddm1*-upregulated TEs, clustered in **Fig. 3f.** Statistical groups were determined by two-tailed Dunn tests with Benjamini-Hochberg correction (P < 0.05), denoted by lowercase letters.

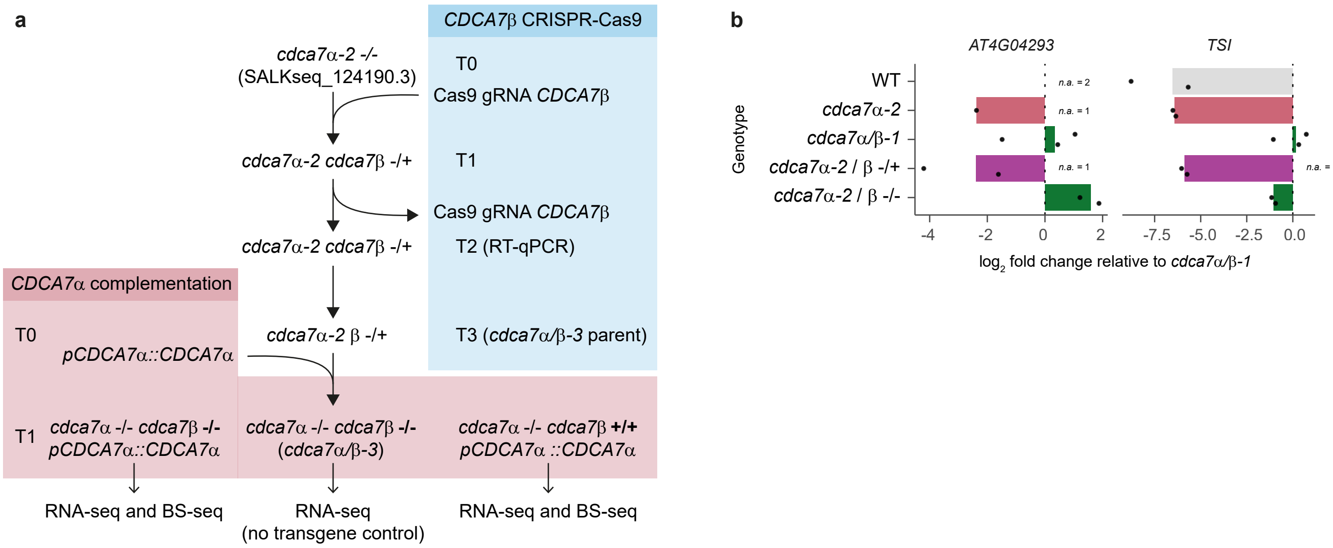

Supplementary Figure 5. Complementation strategy of *cdca7α/β* mutants.

a, Illustration of the genetic scheme used to generate and complement *cdca7α/β* sesquimutants. We mutagenized *CDCA7β* in the *cdca7α-2* mutant background with CRISPR-Cas9. After isolating *cdca7β* mutations in T1, we selected T2 lines with no CRISPR-Cas9 transgene and verified that one *CDCA7β* allele was sufficient to maintain silencing (shown in **b**.). T3 sesquimutants were complemented with a pCDCA7α::CDCA7α-mTurq-cMyc transgene. T1 transformants were isolated with *CDCA7β* homozygous mutant or WT, and we generated transcriptomes (**Fig. 4, 5**) and methylomes (**Fig. 5**). **b**, RT-qPCR quantification of two TE transcripts in *cdca7α/β* mutants, represented as in **a**. In some samples, transcript levels were below the detection limit, indicated by no amplification (n.a.).

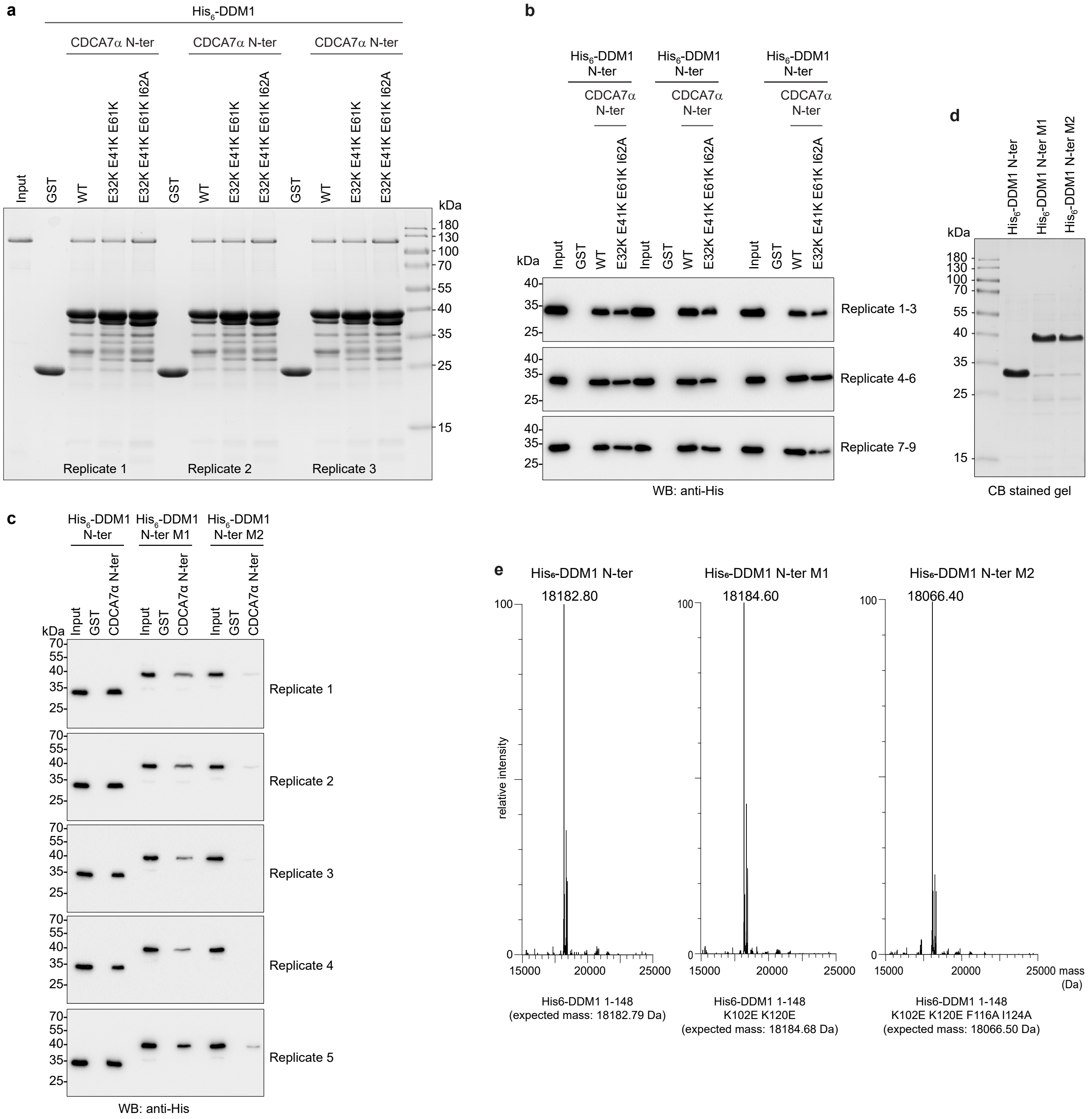

Supplementary Figure 6. Physical interaction between CDCA7α and DDM1.

a, GST-pull-down of His_6-DDM1 (the top band) by GST alone or GST-fused CDCA7α N-terminal fragment (residues 1–136). WT and mutants of CDCA7α were tested in three independent binding reactions. Eluates were resolved by SDS–PAGE and visualised with Coomassie Blue. **b**, GST-pull-down of His_6-DDM1 N-terminus (residues 1–148) with GST-fused CDCA7α N-terminal fragment (residues 1–136). The quadruple CDCA7α mutant retains ~50 % of WT binding capacity (quantification in **Fig. 4g**). **c**, Reciprocal mutagenesis of the DDM1 surface. GST-CDCA7α N-terminal fragment was incubated with His_6-DDM1(1–148) WT, a double mutant (M1: K102E + K120E) or a quadruple mutant (M2: K102E + K120E + F116A + I124A). Five independent pull-downs are shown. (quantified in **Fig. 4h**). **d**, Coomassie-stained SDS–PAGE of the purified His_6-DDM1 proteins used in (**c**). **e**, Intact-mass LC-ESI-MS of the proteins in (**d**). Deconvoluted zero-charge spectra (MaxEnt1) yielded average masses of 18 182.80 Da (WT), 18 184.60 Da (M1) and 18 066.40 Da (M2). The data verify that all proteins are of expected masses.

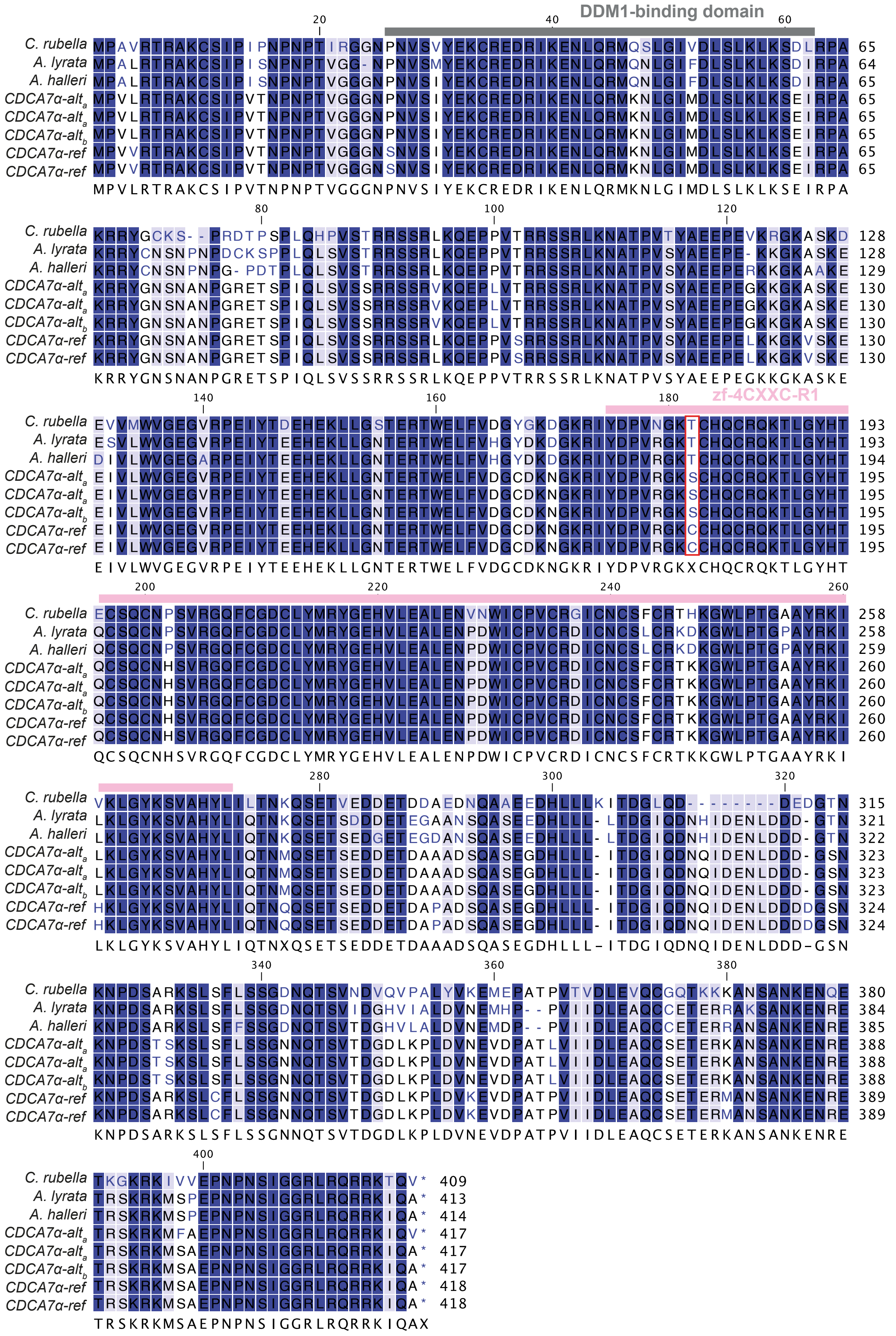

Supplementary Figure 7. Protein sequences of CDCA7α from *A. thaliana* haplotypes and other *Brassicaceae*.

Exon regions were extracted from three CDCA7α alleles and relative species based on a gene model of AtRTD3. The amino acids under the alignment represent consensus sequences. Rectangles indicate the segregating sites between alleles within the conserved domain. The sequences of CDCA7α-alt$_a$ (chr4:17486863$_{non\text{-}ref}$) are extracted from lines 9888 and 6069, according to a previous study[8], the sequence of CDCA7α-alt$_b$ (chr4:17497441$_{non\text{-}ref}$) is from 9543, and the sequences of CDCA7α-ref are extracted from 6909 (Col-0) and 8236.

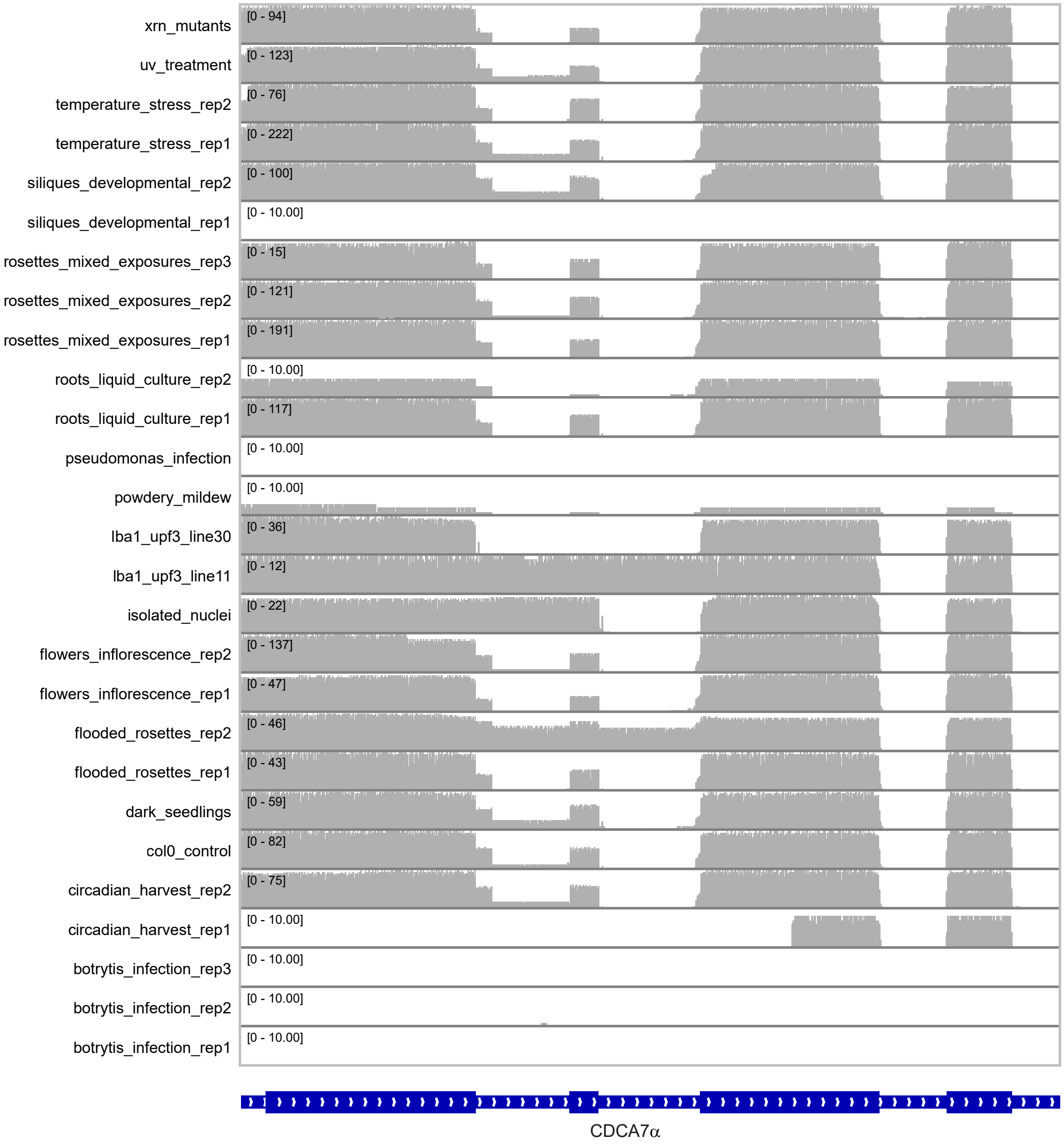

Supplementary Figure 8. *CDCA7α* transcript variants detected by Iso-seq in different conditions.

Iso-seq data from[7], showing varying frequency of transcript variants CDCA7α.2, CDCA7α.3 and CDCA7 α.5. Unnormalized coverage is shown.

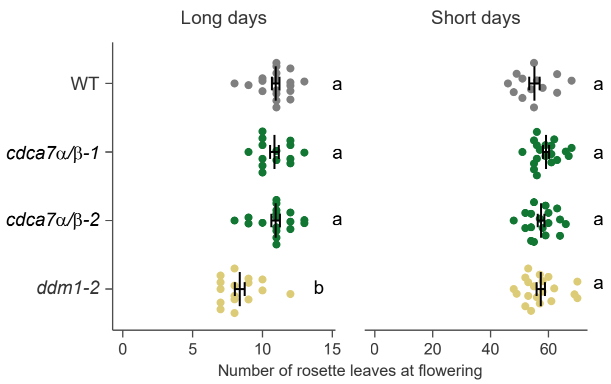

Supplementary Figure 9. Flowering time of *cdca7α/ß* null mutants.

Flowering time, measured as developmental stage at flowering (n = 12 to 19 plants). The statistical groups, indicated by lowercase letters, were determined by one-way ANOVA with two-sided Tukey-Kramer post-hoc tests (P < 1e-05).

Supplementary Table 1. GWAS peaks

Chr	Pos	MAC	-log10(p-value)	Candidate genes (10kbp)
1	14509791	39	6.31 (5.45)	AT1G38440, AT1G38450, AT1G38460, AT1G38550, AT1G38630
4	17486863*	233	5.47 (NA)	AT4G37080, AT4G37090, AT4G37095, AT4G37100, AT4G37110, AT4G37120 (SMP2), AT4G37130, AT4G37140 (ATMES20), AT4G37150 (ATMES9)
4	17497441	41	7.60 (NA)	AT4G37130, AT4G37140 (ATMES20), AT4G37150 (ATMES9), AT4G37160 (SKU5), AT4G37170, AT4G37175, AT4G37180, AT4G37190
5	18598079	95	5.54 (6.50)	AT5G45800 (MEE62), AT5G45810 (CIPK19), AT5G45820 (CIPK20), AT5G45830 (DOG1), AT5G45840, AT5G45850
5	26657906	367	5.80 (6.77)	**AT5G66750 (DDM1)**, AT5G66755, AT5G66760 (SDH1), AT5G66770, AT5G66780, AT5G66790

Peaks were selected at a threshold of -log10(p-value) ≥ 6 in the GWAS or conditional GWAS with 17497441.
* chr4:17486863 is corresponding to a peak detected GWAS for individual TEs

Supplementary Table 2. Origin and description of CDCA7 mutants

mutant	T-DNA insertion	gRNA #1 (mutation)	gRNA #2 (mutation)	background for CRISPR	generation used	generation of homozygous ddm1-2
cdca7a-1	SALK_100123C	-	-	-	-	-
cdca7a-2	SALKseq_124190.3	-	-	-	-	-
cdca7b-1	-	cdca7b gRNA-1 (Δ 542bp)	cdca7b gRNA-6 (Δ 542bp)	Col-0	T3	-
cdca7b-2	-	cdca7b gRNA-1 (none)	cdca7b gRNA-6 (+G)	Col-0	T4	-
cdca7a/b-1	SALKseq_124190.3	cdca7b gRNA-1 (Δ 424bp)	cdca7b gRNA-6 (Δ 424bp)	*cdca7a-2*	T3 & T8	-
cdca7a/b-2	-	cdca7a gRNA-11 (+T)	cdca7b gRNA-6 (+C)	Col-0	T3	-
cdca7a/b-3	SALKseq_124190.3	cdca7b gRNA-1 (Δ 392bp)	cdca7b gRNA-6 (Δ 392bp)	*cdca7a-2*	T4	-
ddm1 cdca7a-1	SALK_100123C	-	-	-	-	G2 (F3 from the cross)
ddm1 cdca7a-2	SALKseq_124190.3	-	-	-	-	G1 (F2 from the cross)
ddm1 cdca7b-1	-	cdca7b gRNA-6 (+A)	cdca7b gRNA-1 (+A)	*ddm1-2 -/- G2*	T4	G6
ddm1 cdca7b-2	-	cdca7b gRNA-4 (+A)	cdca7b gRNA-1 (Δ 27bp)	*ddm1-2 -/- G2*	T4	G6
ddm1 cdca7a/b-1	-	cdca7a gRNA-11 (Δ 28bp)	cdca7b gRNA-6 (+82bp)	*ddm1-2 -/- G2*	T4	G6
ddm1 cdca7a/b-2	-	cdca7a gRNA-11 (+A)	cdca7b gRNA-6 (Δ 80bp)	*ddm1-2 -/- G2*	T4	G6

Supplementary Table 3. List of primers used in this study

experiment	purpose	name	sequence
RT-qPCR	quantifying TE transcripts	ACTIN2 pri3F	GGCTTAAAAAGCTGGGGTTT
RT-qPCR	quantifying TE transcripts	ACTIN2 pri3R	TTGTCACACACAAGTGCATCA
RT-qPCR	quantifying TE transcripts	AT4G04293-qF	GAGGGATCATGTAGCACCAAA
RT-qPCR	quantifying TE transcripts	AT4G04293-qR	GCTCCAACGATTCGAAGAAA
RT-qPCR	quantifying TE transcripts	TSI-qF	CTCTACCCTTTGCATTCATGAATCCTT
RT-qPCR	quantifying TE transcripts	TSI-qR	GATGGGCAAAAGCCCTCGGTTTTAAAATG
RT-qPCR	quantifying TE transcripts	MULE qF	GGCACTTCAATTGTGCTTTTTCCT
RT-qPCR	quantifying TE transcripts	MULE R2	GATACTTGTTGACAAGTGTTTAGCAAGCC
RT-qPCR	quantifying CDCA7α transcript isoforms	CDCA7α.2 qF	CTCTAGACGATCCTCAAGATTGAAG
RT-qPCR	quantifying CDCA7α transcript isoforms	CDCA7α.2 qR	GGTGTAGCGTTCTTCAATCTCG
RT-qPCR	quantifying CDCA7α transcript isoforms	CDCA7α.3 qF	TCAGTCACTGACTTCTCTCGA
RT-qPCR	quantifying CDCA7α transcript isoforms	CDCA7α.3 qR	AGCGTTCTTCAATCTCGAGGA
RT-qPCR	quantifying CDCA7α transcript isoforms	CDCA7α.5 qF	CTCTGTGGTTCTCGTTTCGTTC
RT-qPCR	quantifying CDCA7α transcript isoforms	CDCA7α.5 qR	TCTGCGTAGGAAACTGGTGTAG
cloning	CRISPR-Cas9	CDCA7α_sgRNA_11	ttggtctcaattgTACACCAGTTTCCTACGCAGgttttagagctagaaatag
cloning	CRISPR-Cas9	CDCA7ß_sgRNA_6	ttggtctcaattGCTATGATAAGAATGGGAAAgttttagagctagaaatag
cloning	CRISPR-Cas9	CDCA7ß_sgRNA_1	ttggtctcaattgAACTGGCCCGATTTACAGAAgttttagagctagaaatag
cloning	CRISPR-Cas9	CDCA7ß_sgRNA_4	ttggtctcaattgTGACAAGTCTTACCGTTAAAgttttagagctagaaatag
cloning	recombinant expression in pGEX-4T-1	CDCA7α F EcoR1	tcagaGAATTCATGCCTGTGGTGAGAACGAGAGC
cloning	recombinant expression in pGEX-4T-1	CDCA7α R Sall	tcagaGTCGACTCAAGCTTGAATCTTACGACGCTG
cloning	recombinant expression in pGEX-4T-1	CDCA7ß F EcoR1	tcagaGAATTCATGCTCACCATGAGAACTGAAGC
cloning	recombinant expression in pGEX-4T-1	CDCA7ß R Sall	tcagaGTCGACTTAGGTCCTTCTTCGCTGCCTTAG
cloning	recombinant expression in pGEX-4T-1	CDCA7 F - deletion 1	tcaga gaattc aactcaaacgtctcgatctacga
cloning	recombinant expression in pGEX-4T-1	CDCA7 R - deletion 1	tctgagtcgac ttagggtcggatctcggatttgag
cloning	recombinant expression in pGEX-4T-1	CDCA7 F - deletion 2	tcaga gaattc gccaaaaggcgttacggtaact
cloning	recombinant expression in pGEX-4T-1	CDCA7 R - deletion 2	tctgagtcgac ttatccgagattcttcatcctctga
cloning	cloning CDCA7α promoter	pCDCA7α_5UTR_F-GG_A	GGTGGTGGTCTCCACCTAATTCTAATTTCTTCCTGTGATC
cloning	cloning CDCA7α promoter	pCDCA7α_5UTR_R-GG_B	ACCACCGGTCTCGTGTTTGTGAGAGATTGATGAACTG
cloning	cloning CDCA7ß promoter	pCDCA7ß_5UTR_F-GG_A	GGTGGTGGTCTCCACCTTAATTCGTTGGACGCTTG
cloning	cloning CDCA7ß promoter	pCDCA7ß_5UTR_R-GG_B	ACCACCGGTCTCGTGTTGATGATGATGAAGATGATACTGG
cloning	cloning CDCA7α gene with N-ter tag	CDCA7α_F-GG_C_no_ATG	GGTGGTGGTCTCCGGCTCCTGTGGTGAGAACGAGAG
cloning	cloning CDCA7α gene with N-ter tag	CDCA7α_R-GG_E	ACCACCGGTCTCGGCAGCTTTCTCGATTAGGCTTTTTGG
cloning	cloning CDCA7α gene with C-ter tag	CDCA7α_F-GG_C	GGTGGTGGTCTCCGGCT CT ATGCCTGTGGTGAGAACG
cloning	cloning CDCA7α gene with C-ter tag	CDCA7α_R-GG_D_no_stop	ACCACCGGTCTCGCTGAAGCTTGAATCTTACGACGCTG
cloning	cloning CDCA7α gene with N-ter tag	CDCA7ß_R-GG_E	ACCACCGGTCTCGGCAGGAGATTTTCATAGTATACTTGAAAGTAT
cloning	cloning CDCA7α gene with N-ter tag	CDCA7ß_F-GG_C_no_ATG	GGTGGTGGTCTCCGGCTCTCACCATGAGAACTGAAGC
cloning	cloning CDCA7ß gene with C-ter tag	CDCA7ß_F-GG_C	GGTGGTGGTCTCCGGCT CT ATGCTCACCATGAGAACTG
cloning	cloning CDCA7ß gene with C-ter tag	CDCA7ß_R-GG_D_no_stop	ACCACCGGTCTCGCTGAGGTCCTTCTTCGCTGCC
cloning	remove the endogenous BsaI sites of CDCA7ß	CDCA7ß_BsaImut-F_site1_IVA	AAAGAAACCCCAAAGGTTTCTCTTTACGAACAATGCAGGG
cloning	remove the endogenous BsaI sites of CDCA7ß	CDCA7ß_BsaImut-R_site1_IVA	ACCTTTGGGGTTTCTTTGATTAGTT
cloning	remove the endogenous BsaI sites of CDCA7ß	CDCA7ß_BsaImut-F_site2_IVA	AGGATGAACAATCTGGGACTCTTAAATCTCTCTCGCAAACT
cloning	remove the endogenous BsaI sites of CDCA7ß	CDCA7ß_BsaImut-R_site2_IVA	CCCAGATTGTTCATCCTCTGA

genotyping	sequence CDCA7ß CRISPR mutations	CDCA7ß_gRNA4-6_seq_F	CTCCTCCTCTTCAGCCTTCTCG
genotyping	sequence CDCA7ß CRISPR mutations	CDCA7ß_gRNA1_seq_R	ACGCTTGACTTGGTGTTGTGTC
genotyping	sequence CDCA7α CRISPR mutations	CDCA7α_gRNA11_6beta_seq_F	ATCCGGGTCGAGAAACATCTCC
genotyping	sequence CDCA7α CRISPR mutations / genotype cdca7α-2	CDCA7α_gRNA11_6beta_seq_R	ACACTGGCTGCATTGGGTATGA
genotyping	genotype cdca7α-2 (use with LBb1.3)	LBb1.3	ATTTTGCCGATTTCGGAAC
genotyping	genotype cdca7α-1	SALK_100123.55.70_LP	GCCTTCTCCTACCCACAAAAC
genotyping	genotype cdca7α-1 (use with LBb1.3)	SALK_100123.55.70_RP	GTCGCCATGATTGTTACGATC
genotyping	genotype ddm1-2 (RsaI dCAPS, WT is cut)	ddm1-2 CAPS Fw	GTTGGACAGTGTGGTAAATTCCGCT
genotyping	genotype ddm1-2 (RsaI dCAPS, WT is cut)	ddm1-2 CAPS Rev	GAGCTACGAGCCATGGGTTTGTGAAACGTA

note: CDCA7ß_sgRNA_6 was designed to target both CDCA7α and CDCA7ß but did not induce mutations in CDCA7α

Supplementary Methods

Phylogenetic Analysis of *CDCA7*

To reconstruct the evolutionary history of CDCA7, we constructed orthologous gene clusters (i.e., orthogroups) from two different proteome datasets using sequential, dense taxon sampling across two levels: Archaeplastida (50 species) and Viridiplantae (66 species, enriched for Brassicales) (Supplementary Table 5, 6). This method enabled us to estimate more precisely the timing of the *CDCA7α/β* duplication event. Orthofinder v2.5.2[9] was used to cluster genes in a non-biased manner by comparing each gene to the entire proteome dataset, and DIAMOND[10] for homology search ('-S diamond_ultra_sens'). For the Archaeaplastida-level survey, we included previously-identified metazoan CDCA7s[11] as an outgroup. MAFFT v7.310[12] was used to align protein sequences based on orthogroups. Individual maximum likelihood gene trees were built with IQ-TREE v2.1.2[13], which used model selection ('-m MFP') and an ultrafast bootstrap approximation approach (1,000 replicates). The phylogenetic trees were visualized using iToL v6.7[14].

We used ancestral sequence reconstruction to reconstruct the CDCA7 protein sequence prior to the Brassicaceae CDCA7α/β duplication event. Based on the phylogenetic positions, we selected 48 protein sequences from Brassicaceae CDCA7 homologs and Amborella CDCA7 as an outgroup and aligned them using MAFFT. Following the multiple sequence alignment, IQ-TREE was used to rebuild a phylogenetic tree that will be used as input for the initial ASR analysis. First, we converted the protein sequence alignment to an absence/presence matrix, then used IQ-TREE's ancestral sequence reconstruction ('-asr') option with the rooted phylogenetic tree. The absence/presence ancestral state output was used to trim residues from the protein sequence alignment based on the calculated posterior probability of each residue. The final ancestral sequence reconstruction used the trimmed alignment to generate the ancestral Brassicaceae CDCA7 protein sequence.

Measuring flowering time and seed size

Flowering time was quantified by counting the number of mature rosette leaves at the anthesis of the first flower. Plants were grown in a randomized layout under short-day (8h light/16h dark) or long-day (16h light/8h dark) conditions at 21°C in a climatic chamber. The *cdca7α/β* mutants were in the 5th generation of inbreeding post-CRISPR transformation, while *ddm1-2* mutants were in the 3rd generation.

For seed size measurements, plants were grown in a randomized pattern from seed batches with reduced seed size heterogeneity. Specifically, seeds were sorted on size to exclude the 5% of biggest and smallest seeds, using a Boxeed seed phenotyping robot (Labdeers). Four crosses were performed for each inflorescence, yielding 5 to 47 seeds per silique (mean =

31.08, n = 72). We used either the main floral stem, or the first axillary branch as a female inflorescence. A linear model showed that the mean seed surface area per silique was unaffected by the inflorescence type (P = 0.64) or the number of seeds per silique (P = 0.89), allowing comparisons to be made without accounting for these variables. Seed surface was measured using scanners and ImageJ scripts.

Whole genome bisulfite sequencing (WGBS)

Library construction and sequencing

To measure DNA methylation levels in *cdca7α-2, smp2,* and *AT4G37130* mutants (**Fig. 1e**), genomic DNA was extracted from rosette leaves at 9-true-leaf stage using the GeneJET Plant Genomic DNA Purification Kit (Thermo Scientific) and sheared with an E220 Focused-ultrasonicator (Covaris) to achieve an average fragment size of approximately 350 bp. Sequencing libraries were prepared using the NEBNext Ultra II DNA Library Prep Kit (New England BioLabs) with methylated adapters (New England BioLabs). The adapter-ligated DNA underwent bisulfite conversion using the EZ-96 DNA Methylation-Gold MagPrep Kit (Zymo Research). Bisulfite-treated samples were amplified using EpiMark Hot Start Taq DNA Polymerase and indexed with NEBNext Multiplex Oligos for Illumina (New England BioLabs). All libraries were sequenced on either an Illumina NextSeq 550 or HiSeq 2500 platform.

Estimation of DNA methylation levels

All reads were mapped on the TAIR10 reference genome using a Methylpy pipeline v1.2 (https://github.com/yupenghe/methylpy). DNA methylation levels were estimated as weighted methylation levels for each transposon defined in Araport11 annotation. CMT2- and RdDM-targeted transposons were defined as having differential levels of methylation (> 0.1) between wild-type and *cmt2* or *drm1drm2* in Col-0 as previously described[15]. For each line, average DNA methylation was calculated using all transposons for which at least one read was mapped. Metaplots of mCG at 229 TEs associated with *chr4:17486863* in WT and the loss-of-function *AT4G37110* mutant were generated using the plotProfile function in deepTools v3.1.1[16], following calculation of scaled mCG levels (500 bins) with the computeMatrix function.

Chromatin Immunoprecipitation followed by sequencing

Library construction and sequencing

1.5 g of rosette leaves from 31-d-old plants were fixed in 37 ml 1% formaldehyde in PBS by vacuum-infiltration for 10 min. The cross-linking reaction was quenched by adding glycine to a final concentration of 125 mM. Tissues were frozen in liquid nitrogen and ground in a 5-ml grinding jar on a Retsch Mixer Mill MM 200 at 30 Hz for 45 s. Tubes were kept at room temperature until the powder showed signs of thawing. We added 35 ml of nuclei isolation buffer (NIB, 10 mM MES-KOH pH 5.3, 250 mM sucrose, 10 mM NaCl, 10 mM KCl, 2.5 mM EDTA, 0.1

mM spermine, 0.1 mM spermidine, 2.5 mM ß-mercaptoethanol, 0.3% Triton X-100 and protease inhibitors)[17], followed by vortexing until homogeneous. The extract was filtered through two layers of Miracloth, which were washed with 10 ml of NIB and spun at 3,400 rpm at 4°C for 5 min. Pellets were resuspended in 25 ml of NIB, vortexed and incubated for 10 min on ice to fully dissolve chlorophyll content. Tubes were spun as above and pellets were carefully washed with 5 ml of shearing buffer (10 mM Tris-HCl pH 8.0, 1 mM EDTA, 0.1% SDS, and protease inhibitors) without resuspending and spun again. This washing step was repeated and pellets were resuspended in 0.9 ml of shearing buffer. One ml of the extracted chromatin was transferred to Covaris glass tubes and sonication was conducted at 4°C for 900 seconds per sample (peak power 140.0; duty factor: 5.0; Cycles/Burst: 200). Sheared chromatin was spun for 5 min at 4°C at 15,000 rpm. The supernatant was collected and spun again, and the supernatant was transferred to a 5 ml tube. Fifteen µl of chromatin were kept to control for sonication efficiency.

Chromatin was diluted to 3.5 ml to reduce SDS concentration with a ChIP dilution buffer (16.7 mM Tris-HCl pH 8.0, 167 mM NaCl, 1.2 mM EDTA, 1.1% Triton X-100, 0.01% SDS and protease inhibitors). For preclearing, 200 µl of Dynabeads protein A (Thermofisher, reference 1001D) were added to the chromatin and rotated 1 h at 4°C. Beads were discarded by collecting supernatants after two centrifugations at maximum speed for 30 s. A hundred µl of pre-cleared chromatin was kept at -20°C for input control, and the remaining chromatin was aliquoted with 500 µl per immunoprecipitation (IP) and incubated with 5 µg of anti-H3 (Abcam, ab1791), anti-H2A.W.6/7, anti-H2A.Z.9/11, anti-H3K27me1 (Millipore, 17-643) or anti-H3K9me2 (Abcam, ab1220) antibodies at 4 °C overnight with rotation. After incubation, samples were mixed with 30 µl of protein A magnetic beads, rotated at 4°C for 3 h, and washed two times with a low-salt buffer (20 mM Tris-HCl pH 8.0, 150 mM NaCl, 2 mM EDTA, 1% Triton X-100 and 0.1% SDS), once with a high-salt buffer (20 mM Tris-HCl pH 8.0, 500 mM NaCl, 2 mM EDTA, 1% Triton X-100 and 0.1% SDS), once with a LiCl buffer (10 mM Tris-HCl pH 8.0, 1 mM EDTA, 0.25 M LiCl, 1% IGEPAL CA-630 and 0.1% deoxycholic acid) and twice with TE buffer (10 mM Tris-HCl pH 8.0 and 1 mM EDTA). Elution was done with 200 µl of 0.1 M NaHCO3 and 1% SDS, incubated at 65°C for 15 min. We added 20.4 µl of reverse cross-link buffer (0.4 M Tris-HCl pH 8.0, 2 M NaCl, 10 mM EDTA, 0.6 mg ml−1 proteinase K (Thermo Fisher Scientific)) and incubated at 45°C for 3 h and 65°C for 16 h. RNA was subsequently degraded for 30 min at room temperature with 10 µg of RNase A (Thermo Fisher Scientific), and DNA was purified with the ChIP DNA Clean & Concentrator kit (Zymo, reference D5205).

Libraries were prepared with the NEBNext Ultra II DNA library prep kit for Illumina (New England Biolabs), following the manufacturer's instructions. Fragment size distribution was analyzed on a 5200 Fragment Analyzer System (Agilent), and size selection with SPRI beads (Molecular Biology Services, IMP, Vienna, Austria) was applied to remove large fragments and self-ligated adapters, when necessary. To maximize comparability, if only one sample had undesired fragments, we size-selected all samples for a given antibody. For size selection, briefly, DNA was diluted to 100 µl, we mixed in 60 µl of magnetic SPRI beads, collected the supernatant, and added 40 µl of magnetic SPRI beads, washed two times with 80% ethanol, and eluted DNA. Sequencing was carried out on a NovaSeq 6000 instrument using an S4 flow cell to generate paired-end 150 bp reads with around 25 million reads per sample.

ChIP-seq analysis

The data were analyzed using the nfcore/chipseq pipeline v2.0.0 (https://nf-co.re/chipseq/2.0.0/)[18] with Nextflow (v22.10.7). We used the TAIR10 genome, custom arguments for read length (150 bp), fragment size (275), and blacklisted mitochondrial and chloroplast genomes. To get the average value for each annotation, we used the bigWigAverageOverBed script from UCSC tools (https://github.com/ucscGenomeBrowser/kent-core/tree/master). We further analyzed the data with deepTools v3.3.1[16]. To normalize by H3 or by the WT, we used *bigwigCompare* with a bin size of 10 bp, ignoring non-covered regions with *skipNonCoveredRegions*. To produce average profile plots (metaplots), annotations were scaled to an arbitrary size of 1000 bp and the enrichment value was calculated by 50 bp bins, using *computeMatrix scale-regions*. We defined heterochromatic TEs (n = 12548) as having a minimum level of heterochromatin marks in the WT, using a log_2 ratio (IP / H3) above 0.1 for all replicates of H3K9me2, H3K27me1 and H2A.W, and further filtered out TEs below 100 bp of length.

Protein structure prediction

We used Alphafold3[19] to model protein structures and interactions, with the random seed set to 1. The top-ranking prediction was selected for representation. The predicted structure of CDCA7α with three Zn^{2+} ions was superimposed to *H. sapiens* CDCA7 in a complex with non-B-form DNA containing 5mC (PDB ID 8TLK)[20]. Graphical representations and analyses of structures were done with ChimeraX[21]. To dissect the interaction between CDCA7α/β and DDM1, we used AlphaFold-Multimer[22] with a local implementation of Colabfold[23] and the top-ranking prediction. We used the *alphafold contacts* command to identify interacting residues, and hydrogen bonds and salt bridges were defined with a distance tolerance of 4 Å and an angle tolerance of 40°.

Recombinant protein expression

Cloning

CDCA7α and CDCA7β cDNAs were amplified by RT-PCR with primers indicated in Supplementary Table 3, using RNA purified from WT flowers. We used the canonical CDCA7α.2 transcript variant according to AtRTD3[8]. cDNAs were cloned into pGEX-4T-1 (Cytiva) using *EcoRI*/*SalI*. DDM1 cDNA into pET15b was previously described[24]. Plasmids expressing N-terminal fragments of CDCA7α (1-136) and DDM1 (1-146) and its point mutants in pGEX-4T-1 and pET15b, respectively, were obtained from GenScript.

Expression and purification of recombinant proteins and *in vitro* pull-down experiments

BL21 (DE3) RIL *E. coli* cells transformed with plasmids for expression of full-length CDCA7α, its deletion mutants (**Fig. 4**), and DDM1 were grown at 37°C overnight in 100 ml LB. Cultures were diluted in 1L (CDCA7α) or 2L (DDM1) of LB and grown for three hours at 20ºC and then induced for 7 hours at 20ºC with 1 mM IPTG. For the expression of N-terminal fragments of CDCA7α (1-136) and DDM1 (1-146) containing point mutations or deletions (**Fig. 4**) overnight cultures were diluted 1:100 in 200 mL of LB and grown for 2 hours at 37ºC and then induced for three hours at 37ºC with 1 mM IPTG. For GST-tagged CDCA7α and its deletion mutants (1L cultures), cell pellets were resuspended in 20 ml of extraction buffer (50 mM Tris-HCl pH 8.0, 1 M NaCl, 1 mM DTT, 0.1% Triton X-100) containing protease inhibitors (Roche), 10 µl of benzonase (1 mg/ml) and 50 mg of lysozyme. After sonication (Bioruptor, Diagenode) for 10 min at high intensity (5'' on / 5'' off) and 5 min at medium intensity (5'' on / 5'' off), extracts were centrifuged for 15 min at 4°C at 40,000 × *g*. Extracts were incubated with 500 µl of glutathione Sepharose 4 fast flow (Cytiva) at RT for one hour and then transferred to disposable columns and washed with 5 column volumes of extraction buffer. Proteins were eluted with six 300 µl elution steps with 50 mM Tris-HCl pH 8.0, 500 mM NaCl buffer containing 20 mM reduced glutathione and 1 mM DTT.

For purification of His_6- or His_6SUMO-tagged DDM1, cell pellets from 2 L cultures were resuspended in 25 ml of extraction buffer (50 mM Tris-HCl pH 7.5, 500 mM NaCl, 2 mM DTT, 0.05% NP-40) containing protease inhibitors (Roche), 10 µl of benzonase (1 mg/ml) and 50 mg of lysozyme and processed as for GST-tagged proteins. Extracts were incubated with 500 µl of Ni-NTA beads (Qiagen) for one hour at RT and then transferred to disposable columns and washed with 5 column volumes of extraction buffer containing 5 mM imidazole but without benzonase and lysozyme. Proteins were eluted with six 400 µl elution steps with elution buffer (50 mM Tris-HCl pH 7.5, 500 mM NaCl, 2 mM DTT, 300 mM imidazole).

Purification of CDCA7α (1-136) and DDM1 (1-146) N-terminal fragments containing point mutations (200 mL cultures) was done as described for the full-length proteins except that the cell pellets were resuspended in 5 mL of corresponding extraction buffer.

In all purifications, elution fractions were analyzed on 10-12% SDS-PAGE, pooled, and buffer was exchanged into 20 mM Tris-HCl pH 7.5, 100 mM NaCl, 1 mM DTT by centrifugation over Amicon Ultra-15 30 kDa and 50 kDa cut-off centrifugal filters (Millipore) for GST-tagged CDCA7 and His_6- or His_6SUMO-tagged DDM1, respectively. For CDCA7α (1-136) and DDM1 (1-146) N-terminal fragments containing point mutations buffer exchange was done by centrifugation over Amicon Ultra-15 10 kDa cut-off centrifugal filters (Millipore).

For pull-down, equimolar amounts of GST-tagged proteins along with GST alone were mixed with 5 µg of His_6SUMO-DDM1 or His_6-DDM1 and incubated with 10 µl of magnetic glutathione beads (Thermo Fisher Scientific) for 90 min at RT in binding buffer (20 mM Tris-HCl, pH 7.5, 100 mM NaCl, 1 mM DTT, 0.1% NP-40). Pull-downs with CDCA7α (1-136) and DDM1 (1-146) N-terminal fragments containing point mutants were done in binding buffer containing 150 mM NaCl. Beads were washed 6 times for 5 min with ice cold binding buffer, denatured in 30 µl of 1

× SDS-PAGE loading buffer and 10 µL were loaded on 10% or 15% SDS-PAGE and analyzed by western blotting with anti-DDM1 or anti-His antibodies. Input lanes were loaded with 1/20 of protein used for pull-down.

Intact mass spectrometry

Because DDM1 N-terminal fragments displayed aberrant molecular weights on SDS-PAGE (**Supplementary Fig. 6d**), we analyzed their masses and identity by mass spectrometry. Intact protein samples were diluted in H_2O and 2pmol protein were loaded on an XBridge Protein BEH C4 column (2.5 µm particle size, dimensions 2.1 mm X 150 mm; Waters) using a Vanquish™ Horizon UHPLC System (Thermo Scientific) with a working temperature of 50 °C, 0.1% formic acid (FA) as solvent A, 100% acetonitrile, 0.08% FA as solvent B. Proteins were separated on a 6 min step gradient from 12 to 40 and then to 72% solvent B at a flow rate of 250 µL/min and analyzed on a Synapt G2-Si coupled via a ZSpray ESI source (both Waters). Data were recorded with MassLynx V 4.2 (Waters) and analyzed using the MaxEnt1 process to reconstruct the uncharged average protein mass.

Supplementary References

1. Nartey, W., Goodarzi, A. A. & Williams, G. J. Cryo-EM structure of DDM1-HELLS chimera bound to nucleosome reveals a mechanism of chromatin remodeling and disease regulation. *bioRxiv* (2023) doi:10.1101/2023.08.09.551721.
2. Lee, S. C. *et al.* Chromatin remodeling of histone H3 variants by DDM1 underlies epigenetic inheritance of DNA methylation. *Cell* **186**, 4100–4116.e15 (2023).
3. Klepikova, A. V., Kasianov, A. S., Gerasimov, E. S., Logacheva, M. D. & Penin, A. A. A high resolution map of the Arabidopsis thaliana developmental transcriptome based on RNA-seq profiling. *Plant J.* **88**, 1058–1070 (2016).
4. Lyons, D. B. & Zilberman, D. DDM1 and Lsh remodelers allow methylation of DNA wrapped in nucleosomes. *Elife* **6**, (2017).
5. Bernatavichute, Y. V., Zhang, X., Cokus, S., Pellegrini, M. & Jacobsen, S. E. Genome-wide association of histone H3 lysine nine methylation with CHG DNA methylation in Arabidopsis thaliana. *PLoS One* **3**, e3156 (2008).
6. Maheshwari, S., Ishii, T., Brown, C. T., Houben, A. & Comai, L. Centromere location in

Arabidopsis is unaltered by extreme divergence in CENH3 protein sequence. *Genome Res.* **27**, 471–478 (2017).

7. 1001 Genomes Consortium. Electronic address: magnus.nordborg@gmi.oeaw.ac.at & 1001 Genomes Consortium. 1,135 genomes reveal the global pattern of polymorphism in Arabidopsis thaliana. *Cell* **166**, 481–491 (2016).
8. Zhang, R. *et al.* A high-resolution single-molecule sequencing-based Arabidopsis transcriptome using novel methods of Iso-seq analysis. *Genome Biol.* **23**, 149 (2022).
9. Emms, D. M. & Kelly, S. OrthoFinder: phylogenetic orthology inference for comparative genomics. *Genome Biol.* **20**, 238 (2019).
10. Buchfink, B., Reuter, K. & Drost, H.-G. Sensitive protein alignments at tree-of-life scale using DIAMOND. *Nat. Methods* **18**, 366–368 (2021).
11. Funabiki, H., Wassing, I. E., Jia, Q., Luo, J.-D. & Carroll, T. Coevolution of the CDCA7-HELLS ICF-related nucleosome remodeling complex and DNA methyltransferases. *eLife* (2023) doi:10.7554/elife.86721.
12. Katoh, K. & Standley, D. M. MAFFT multiple sequence alignment software version 7: improvements in performance and usability. *Mol. Biol. Evol.* **30**, 772–780 (2013).
13. Minh, B. Q. *et al.* IQ-TREE 2: New models and efficient methods for phylogenetic inference in the genomic era. *Mol. Biol. Evol.* **37**, 1530–1534 (2020).
14. Letunic, I. & Bork, P. Interactive Tree of Life (iTOL) v6: recent updates to the phylogenetic tree display and annotation tool. *Nucleic Acids Res.* **52**, W78–W82 (2024).
15. Kawakatsu, T. *et al.* Epigenomic Diversity in a Global Collection of Arabidopsis thaliana Accessions. *Cell* **166**, 492–505 (2016).
16. Ramírez, F. *et al.* deepTools2: a next generation web server for deep-sequencing data analysis. *Nucleic Acids Res.* **44**, W160–5 (2016).
17. Lorković, Z. J., Hilscher, J. & Barta, A. Use of fluorescent protein tags to study nuclear organization of the spliceosomal machinery in transiently transformed living plant cells. *Mol.*

Biol. Cell **15**, 3233–3243 (2004).

18. Ewels, P. A. *et al.* The nf-core framework for community-curated bioinformatics pipelines. *Nat. Biotechnol.* **38**, 276–278 (2020).
19. Abramson, J. *et al.* Accurate structure prediction of biomolecular interactions with AlphaFold 3. *Nature* **630**, 493–500 (2024).
20. Hardikar, S. *et al.* The ICF syndrome protein CDCA7 harbors a unique DNA binding domain that recognizes a CpG dyad in the context of a non-B DNA. *Sci. Adv.* **10**, eadr0036 (2024).
21. Meng, E. C. *et al.* UCSF ChimeraX: Tools for structure building and analysis. *Protein Sci.* **32**, e4792 (2023).
22. Evans, R. *et al.* Protein complex prediction with AlphaFold-Multimer. *bioRxiv* 2021.10.04.463034 (2022) doi:10.1101/2021.10.04.463034.
23. Mirdita, M. *et al.* ColabFold: making protein folding accessible to all. *Nat. Methods* **19**, 679–682 (2022).
24. Osakabe, A. *et al.* The chromatin remodeler DDM1 prevents transposon mobility through deposition of histone variant H2A.W. *Nat. Cell Biol.* **23**, 391–400 (2021).
