# Supplementary material for: Major alleles of CDCA7 shape CG methylation in Arabidopsis thaliana
Source: Nat Plants. 2025 Nov 7;11(12):2511–30. doi: 10.1038/s41477-025-02148-w (PMC12711577; doi:10.1038/s41477-025-02148-w)
Supplement: Supplementary file 2 — Reporting Summary [file 41477_2025_2148_MOESM2_ESM.pdf]

nature portfolio

Corresponding author(s): Pierre Bourguet; Frédéric Berger; Eriko Sasaki

Last updated by author(s): Sep 23, 2025

Reporting Summary

Nature Portfolio wishes to improve the reproducibility of the work that we publish. This form provides structure for consistency and transparency in reporting. For further information on Nature Portfolio policies, see our Editorial Policies and the Editorial Policy Checklist.

Statistics

For all statistical analyses, confirm that the following items are present in the figure legend, table legend, main text, or Methods section.

n/a	Confirmed	
☐	☒	The exact sample size (*n*) for each experimental group/condition, given as a discrete number and unit of measurement
☐	☒	A statement on whether measurements were taken from distinct samples or whether the same sample was measured repeatedly
☐	☒	The statistical test(s) used AND whether they are one- or two-sided<br>*Only common tests should be described solely by name; describe more complex techniques in the Methods section.*
☐	☒	A description of all covariates tested
☐	☒	A description of any assumptions or corrections, such as tests of normality and adjustment for multiple comparisons
☐	☒	A full description of the statistical parameters including central tendency (e.g. means) or other basic estimates (e.g. regression coefficient) AND variation (e.g. standard deviation) or associated estimates of uncertainty (e.g. confidence intervals)
☐	☒	For null hypothesis testing, the test statistic (e.g. *F*, *t*, *r*) with confidence intervals, effect sizes, degrees of freedom and *P* value noted<br>*Give P values as exact values whenever suitable.*
☒	☐	For Bayesian analysis, information on the choice of priors and Markov chain Monte Carlo settings
☐	☒	For hierarchical and complex designs, identification of the appropriate level for tests and full reporting of outcomes
☐	☒	Estimates of effect sizes (e.g. Cohen's *d*, Pearson's *r*), indicating how they were calculated

Our web collection on statistics for biologists contains articles on many of the points above.

Software and code

Policy information about availability of computer code

Data collection

Provide a description of all commercial, open source and custom code used to collect the data in this study, specifying the version used OR state that no software was used.

Data analysis

For analysis and plotting:
R Statistical Software (R v4.3.2; https://www.R-project.org/) with the following packages: tidyverse 2.0.0, ggplot2 3.5.1 (Wickham, 2016; Wickham et al., 2019), patchwork 1.2.0, agricolae 1.3-7 , ggbeeswarm 0.7.2, MASS 7.3-60, elevatr (https://patchwork.data-imaginist.com, https://cran.r-project.org/package=agricolae, https://cran.r-project.org/package=ggbeeswarm, https://cran.r-project.org/package=MASS, https://github.com/USEPA/elevatr).

Genome-wide bisulfite analyses:
Methylpy pipeline v1.2 (https://github.com/yupenghe/methylpy)
The nfcore methylseq pipeline v2.3.0 (https://nf-co.re/methylseq) (Ewels et al., 2020) with Nextflow (v22.10.7)
bigWigAverageOverBed script from UCSC tools (https://github.com/ucscGenomeBrowser/kent-core/tree/master)

3' tag-sequencing of messenger RNAs:
custom script (https://github.com/pierre-bourguet/3-tag-rna-seq)
DESeq2 v1.42.1 (Love, Huber and Anders, 2014)

Measurement of expression in 1001 genomes:
STAR v.2.9.6 (Dobin et al. 2013)
featurecounts (Liao et al. 2014)

Chip-seq:
The nfcore/chipseq pipeline v2.0.0 (https://nf-co.re/chipseq/2.0.0/) (Ewels et al., 2020) with Nextflow (v22.10.7)
UCSC tools (https://github.com/ucscGenomeBrowser/kent-core/tree/master)
deepTools v3.3.1 (Ramírez et al., 2016)

Protein structure prediction:
Alphafold3 (Abramson et al., 2024)
Colabfold (Mirdita et al., 2022)
ChimeraX (Meng et al., 2023)

Genome-wide association study:
LIMIX version 3.0.4 (Lippert et al., 2014)

Mediation analysis and heritability:
Published programs written by R (Sasaki, Frommlet and Nordborg, 2018)

Geographical analysis:
QGIS 3.38.2 (https://www.qgis.org/)

Pylogenetic analysis:
MAFFT v7.310 (Katoh and Standley, 2013)
IQ-TREE v2.1.2 (Minh et al., 2020)
ape in R v3.3.1 (Paradis, E. and Schliep, K., 2019)
iToL v6.7 (Letunic and Bork, 2024)

For manuscripts utilizing custom algorithms or software that are central to the research but not yet described in published literature, software must be made available to editors and reviewers. We strongly encourage code deposition in a community repository (e.g. GitHub). See the Nature Portfolio guidelines for submitting code & software for further information.

Data

Policy information about availability of data

All manuscripts must include a data availability statement. This statement should provide the following information, where applicable:

- Accession codes, unique identifiers, or web links for publicly available datasets
- A description of any restrictions on data availability
- For clinical datasets or third party data, please ensure that the statement adheres to our policy

The data described in this publication have been deposited in NCBI's Gene Expression Omnibus and are accessible through GEO Series accession number GSE284119 (T-WGBS), GSE283989 (WGBS), GSE283987 (ChIP-seq), GSE284067 (3' tag mRNA-seq).

Research involving human participants, their data, or biological material

Policy information about studies with human participants or human data. See also policy information about sex, gender (identity/presentation), and sexual orientation and race, ethnicity and racism.

Reporting on sex and gender	Not applicable
Reporting on race, ethnicity, or other socially relevant groupings	Not applicable
Population characteristics	Not applicable
Recruitment	Not applicable
Ethics oversight	Not applicable

Note that full information on the approval of the study protocol must also be provided in the manuscript.

Field-specific reporting

Please select the one below that is the best fit for your research. If you are not sure, read the appropriate sections before making your selection.

☒ Life sciences ☐ Behavioural & social sciences ☐ Ecological, evolutionary & environmental sciences

For a reference copy of the document with all sections, see nature.com/documents/nr-reporting-summary-flat.pdf

Life sciences study design

All studies must disclose on these points even when the disclosure is negative.

Sample size	For the population genomics study, we used 774 lines for DNA methylation analysis and 461 lines for gene expression analysis. Since the identified genetic variants were confirmed through experiments, we expect the sample size is sufficient to meet the objectives of this study. For the confirmation of allelic effects, we used one line per allele because the sequences are shared by several lines carrying the alleles and the representative sequence is expected to be sufficient to confirm the allelic effects.<br>For the experimental part, sample sizes were not estimated in advance.
Data exclusions	Libraries with insufficient read coverage or low quality were excluded.
Replication	Independent biological replicates were used, and mutant phenotypes were further validated by using two independent CRISPR-Cas9 lines for each mutant combination. The number of biological replications is described in the corresponding figure legends or the Methods section. In the population genomics studies, no replication was used because each genome sequence was confirmed by comparison with other accessions. Furthermore, the reproducibility of phenotypes was confirmed through their association with genotypes.
Randomization	Plants were grown in a randomized pattern to mitigate position effects.
Blinding	Blinding tests are not common for this type of experiment, as objective values were measured with appropriate controls in all experiments.

Reporting for specific materials, systems and methods

We require information from authors about some types of materials, experimental systems and methods used in many studies. Here, indicate whether each material, system or method listed is relevant to your study. If you are not sure if a list item applies to your research, read the appropriate section before selecting a response.

Materials & experimental systems

n/a	Involved in the study	
☐	☒	Antibodies
☒	☐	Eukaryotic cell lines
☒	☐	Palaeontology and archaeology
☒	☐	Animals and other organisms
☒	☐	Clinical data
☒	☐	Dual use research of concern
☐	☒	Plants

Methods

n/a	Involved in the study	
☐	☒	ChIP-seq
☒	☐	Flow cytometry
☒	☐	MRI-based neuroimaging

Antibodies

Antibodies used	anti-H3 (Abcam, ab1791), anti-H2A.W.6/7 (custom), anti-H2A.Z.9/11 (custom), anti-H3K27me1 (Millipore, 17-643) or anti-H3K9me2 (Abcam, ab1220)
Validation	Custom antibodies were validated in previous studies:<br>anti-H2A.W.6 & anti-H2A.Z.9 (Yelagandula et al. 2014 Cell)<br>anti-H2A.W.7 (Lorkovic et al. 2017 Current Biology)<br>anti-H2A.Z.11 (Jamge et al. 2023 Elife)

Dual use research of concern

Policy information about dual use research of concern

Hazards

Could the accidental, deliberate or reckless misuse of agents or technologies generated in the work, or the application of information presented in the manuscript, pose a threat to:

No	Yes	
☒	☐	Public health
☒	☐	National security
☒	☐	Crops and/or livestock
☒	☐	Ecosystems
☒	☐	Any other significant area

Experiments of concern

Does the work involve any of these experiments of concern:

No	Yes	
☒	☐	Demonstrate how to render a vaccine ineffective
☒	☐	Confer resistance to therapeutically useful antibiotics or antiviral agents
☒	☐	Enhance the virulence of a pathogen or render a nonpathogen virulent
☒	☐	Increase transmissibility of a pathogen
☒	☐	Alter the host range of a pathogen
☒	☐	Enable evasion of diagnostic/detection modalities
☒	☐	Enable the weaponization of a biological agent or toxin
☒	☐	Any other potentially harmful combination of experiments and agents

Plants

Seed stocks

cdca7α-1 (SALK_100123C), cdca7α-2 (SALKseq_124190.3), ddm1-2 (Vongs et al., 1993) mutant and control plants were in the Col-0 genetic background. To reset the effect of the transgenerational aggravation of the ddm1-2 mutant phenotype (Kakutani et al., 1996), we backcrossed a ddm1-2 homozygous mutant six times to Col-0, keeping ddm1-2 heterozygous throughout, and subsequently isolated homozygous ddm1-2 mutants which were considered first generation for this study.

Novel plant genotypes

All transgenic lines were generated with Agrobacterium tumefaciens GV3101 by floral dipping. CRISPR mutants were generated by transforming Col-0, ddm1-2 second-generation mutants, or cdca7α-2 mutants. We obtained stable homozygous mutant lines in T2 or T3, and used their progeny for this study. Details about primers, guide RNAs and CRISPR mutations used in this study can be found in Supplementary Tables 2 and 3.
For complementation, CDCA7α and CDCA7β were cloned from genomic DNA to include endogenous regulatory sequences in promoters, 5'UTRs, and introns. Deletions and point mutations were introduced in constructs using in vivo cloning (García-Nafría et al. 2016). The generations and number of lines used for each experiment are indicated in the text and figure legends.

Authentication

CRISPR-Cas9-induced mutations were identified by Sanger sequencing in primary transformants, and the transgene was segregated out for subsequent generations, where we confirmed the mutations by Sanger sequencing. We used two independent CRISPR-Cas9 lines for each mutant, to control for potential genetic aberrations. Complementations with CDCA7α and CDCA7β confirmed that no confounding off-target mutations contributed to the measured phenotypes. For complementations, multiple independent primary transformants were analyzed.
Since the sgRNA #6 for CDCA7β had the potential to mutate CDCA7α, we verified mutations in CDCA7α for that particular guide.

ChIP-seq

Data deposition

☒ Confirm that both raw and final processed data have been deposited in a public database such as GEO.

☐ Confirm that you have deposited or provided access to graph files (e.g. BED files) for the called peaks.

Data access links
May remain private before publication.

https://www.ncbi.nlm.nih.gov/geo/query/acc.cgi?acc=GSE283987

Files in database submission

cdca7ab_1_H2AW_log2_H3_R1.bigWig
cdca7ab_1_H2AW_log2_H3_R2.bigWig
cdca7ab_1_H2AZ_log2_H3_R1.bigWig
cdca7ab_1_H2AZ_log2_H3_R2.bigWig
cdca7ab_1_H3K27me1_log2_H3_R2.bigWig
cdca7ab_1_H3K9me2_log2_H3_R1.bigWig
cdca7ab_1_H3K9me2_log2_H3_R2.bigWig
cdca7ab_1_H3_R1.bigWig
cdca7ab_1_H3_R2.bigWig
cdca7ab_1_input_R1.bigWig
cdca7ab_1_input_R2.bigWig
cdca7ab_2_H2AW_log2_H3_R1.bigWig
cdca7ab_2_H2AW_log2_H3_R2.bigWig
cdca7ab_2_H2AZ_log2_H3_R1.bigWig
cdca7ab_2_H2AZ_log2_H3_R2.bigWig
cdca7ab_2_H3K27me1_log2_H3_R1.bigWig
cdca7ab_2_H3K27me1_log2_H3_R2.bigWig
cdca7ab_2_H3K9me2_log2_H3_R1.bigWig
cdca7ab_2_H3_R1.bigWig

cdca7ab_2_H3_R2.bigWig
cdca7ab_2_input_R1.bigWig
cdca7ab_2_input_R2.bigWig
Col_0_H2AW_log2_H3_R1.bigWig
Col_0_H2AW_log2_H3_R2.bigWig
Col_0_H2AZ_log2_H3_R1.bigWig
Col_0_H2AZ_log2_H3_R2.bigWig
Col_0_H3K27me1_log2_H3_R1.bigWig
Col_0_H3K27me1_log2_H3_R2.bigWig
Col_0_H3K9me2_log2_H3_R1.bigWig
Col_0_H3K9me2_log2_H3_R2.bigWig
Col_0_H3_R1.bigWig
Col_0_H3_R2.bigWig
Col_0_input_R1.bigWig
Col_0_input_R2.bigWig
ddm1_H2AW_log2_H3_R1.bigWig
ddm1_H2AW_log2_H3_R2.bigWig
ddm1_H2AZ_log2_H3_R1.bigWig
ddm1_H2AZ_log2_H3_R2.bigWig
ddm1_H3K27me1_log2_H3_R1.bigWig
ddm1_H3K27me1_log2_H3_R2.bigWig
ddm1_H3K9me2_log2_H3_R1.bigWig
ddm1_H3K9me2_log2_H3_R2.bigWig
ddm1_H3_R1.bigWig
ddm1_H3_R2.bigWig
ddm1_input_R1.bigWig
ddm1_input_R2.bigWig
cdca7ab_1_H2A.W.6_7_rep1_R1.fastq.gz
cdca7ab_1_H2A.W.6_7_rep1_R2.fastq.gz
cdca7ab_1_H2A.W.6_7_rep2_R1.fastq.gz
cdca7ab_1_H2A.W.6_7_rep2_R2.fastq.gz
cdca7ab_1_H2A.Z.9_11_rep1_R1.fastq.gz
cdca7ab_1_H2A.Z.9_11_rep1_R2.fastq.gz
cdca7ab_1_H2A.Z.9_11_rep2_R1.fastq.gz
cdca7ab_1_H2A.Z.9_11_rep2_R2.fastq.gz
cdca7ab_1_H3K27me1_rep2_R1.fastq.gz
cdca7ab_1_H3K27me1_rep2_R2.fastq.gz
cdca7ab_1_H3K9me2_rep1_R1.fastq.gz
cdca7ab_1_H3K9me2_rep1_R2.fastq.gz
cdca7ab_1_H3K9me2_rep2_R1.fastq.gz
cdca7ab_1_H3K9me2_rep2_R2.fastq.gz
cdca7ab_1_H3_rep1_R1.fastq.gz
cdca7ab_1_H3_rep1_R2.fastq.gz
cdca7ab_1_H3_rep2_R1.fastq.gz
cdca7ab_1_H3_rep2_R2.fastq.gz
cdca7ab_1_input_rep1_R1.fastq.gz
cdca7ab_1_input_rep1_R2.fastq.gz
cdca7ab_1_input_rep2_R1.fastq.gz
cdca7ab_1_input_rep2_R2.fastq.gz
cdca7ab_2_H2A.W.6_7_rep1_R1.fastq.gz
cdca7ab_2_H2A.W.6_7_rep1_R2.fastq.gz
cdca7ab_2_H2A.W.6_7_rep2_R1.fastq.gz
cdca7ab_2_H2A.W.6_7_rep2_R2.fastq.gz
cdca7ab_2_H2A.Z.9_11_rep1_R1.fastq.gz
cdca7ab_2_H2A.Z.9_11_rep1_R2.fastq.gz
cdca7ab_2_H2A.Z.9_11_rep2_R1.fastq.gz
cdca7ab_2_H2A.Z.9_11_rep2_R2.fastq.gz
cdca7ab_2_H3K27me1_rep1_R1.fastq.gz
cdca7ab_2_H3K27me1_rep1_R2.fastq.gz
cdca7ab_2_H3K27me1_rep2_R1.fastq.gz
cdca7ab_2_H3K27me1_rep2_R2.fastq.gz
cdca7ab_2_H3K9me2_rep1_R1.fastq.gz
cdca7ab_2_H3K9me2_rep1_R2.fastq.gz
cdca7ab_2_H3_rep1_R1.fastq.gz
cdca7ab_2_H3_rep1_R2.fastq.gz
cdca7ab_2_H3_rep2_R1.fastq.gz
cdca7ab_2_H3_rep2_R2.fastq.gz
cdca7ab_2_input_rep1_R1.fastq.gz
cdca7ab_2_input_rep1_R2.fastq.gz
cdca7ab_2_input_rep2_R1.fastq.gz
cdca7ab_2_input_rep2_R2.fastq.gz
Col_0_H2A.W.6_7_rep1_R1.fastq.gz
Col_0_H2A.W.6_7_rep1_R2.fastq.gz
Col_0_H2A.W.6_7_rep2_R1.fastq.gz
Col_0_H2A.W.6_7_rep2_R2.fastq.gz
Col_0_H2A.Z.9_11_rep1_R1.fastq.gz

Col_0_H2A.Z.9_11_rep1_R2.fastq.gz
Col_0_H2A.Z.9_11_rep2_R1.fastq.gz
Col_0_H2A.Z.9_11_rep2_R2.fastq.gz
Col_0_H3K27me1_rep1_R1.fastq.gz
Col_0_H3K27me1_rep1_R2.fastq.gz
Col_0_H3K27me1_rep2_R1.fastq.gz
Col_0_H3K27me1_rep2_R2.fastq.gz
Col_0_H3K9me2_rep1_R1.fastq.gz
Col_0_H3K9me2_rep1_R2.fastq.gz
Col_0_H3K9me2_rep2_R1.fastq.gz
Col_0_H3K9me2_rep2_R2.fastq.gz
Col_0_H3_rep1_R1.fastq.gz
Col_0_H3_rep1_R2.fastq.gz
Col_0_H3_rep2_R1.fastq.gz
Col_0_H3_rep2_R2.fastq.gz
Col_0_input_rep1_R1.fastq.gz
Col_0_input_rep1_R2.fastq.gz
Col_0_input_rep2_R1.fastq.gz
Col_0_input_rep2_R2.fastq.gz
ddm1_H2A.W.6_7_rep1_R1.fastq.gz
ddm1_H2A.W.6_7_rep1_R2.fastq.gz
ddm1_H2A.W.6_7_rep2_R1.fastq.gz
ddm1_H2A.W.6_7_rep2_R2.fastq.gz
ddm1_H2A.Z.9_11_rep1_R1.fastq.gz
ddm1_H2A.Z.9_11_rep1_R2.fastq.gz
ddm1_H2A.Z.9_11_rep2_R1.fastq.gz
ddm1_H2A.Z.9_11_rep2_R2.fastq.gz
ddm1_H3K27me1_rep1_R1.fastq.gz
ddm1_H3K27me1_rep1_R2.fastq.gz
ddm1_H3K27me1_rep2_R1.fastq.gz
ddm1_H3K27me1_rep2_R2.fastq.gz
ddm1_H3K9me2_rep1_R1.fastq.gz
ddm1_H3K9me2_rep1_R2.fastq.gz
ddm1_H3K9me2_rep2_R1.fastq.gz
ddm1_H3K9me2_rep2_R2.fastq.gz
ddm1_H3_rep1_R1.fastq.gz
ddm1_H3_rep1_R2.fastq.gz
ddm1_H3_rep2_R1.fastq.gz
ddm1_H3_rep2_R2.fastq.gz
ddm1_input_rep1_R1.fastq.gz
ddm1_input_rep1_R2.fastq.gz
ddm1_input_rep2_R1.fastq.gz
ddm1_input_rep2_R2.fastq.gz

Genome browser session (e.g. UCSC): https://genome.ucsc.edu/s/pbourguet/ChIPseq_CDCA7_GWAS_MS

Methodology

Replicates	We used two independent mutant lines for each CRISPR mutant. The number of biological replicates for each line was 1 to 2 for ChIP-seq.
Sequencing depth	28-80 M input reads, 98% average unique mappers, paired-end 150 bp reads.
Antibodies	anti-H3 (Abcam, ab1791), anti-H2A.W.6/7 (custom), anti-H2A.Z.9/11 (custom), anti-H3K27me1 (Millipore, 17-643) or anti-H3K9me2 (Abcam, ab1220)
Peak calling parameters	We did not use peak calling.
Data quality	We looked at multiple metrics to evaluate data quality:<br>- base calling quality<br>- % of trimmed bases<br>- mapping rates<br>- duplication rates<br>- agreement with the independent mutant lines and biological replicates<br>- efficiency & fingerprint
Software	nfcore/chipseq pipeline v2.0.0 (https://nf-co.re/chipseq/2.0.0/) (Ewels et al., 2020)
