# Supplementary material for: Major alleles of CDCA7 shape CG methylation in Arabidopsis thaliana
Source: Nat Plants. 2025 Nov 7;11(12):2511–30. doi: 10.1038/s41477-025-02148-w (PMC12711577; doi:10.1038/s41477-025-02148-w)
Supplement: Supplementary file 6 — Unprocessed western blots. [file 41477_2025_2148_MOESM6_ESM.pdf]

Source data for Figure 4d

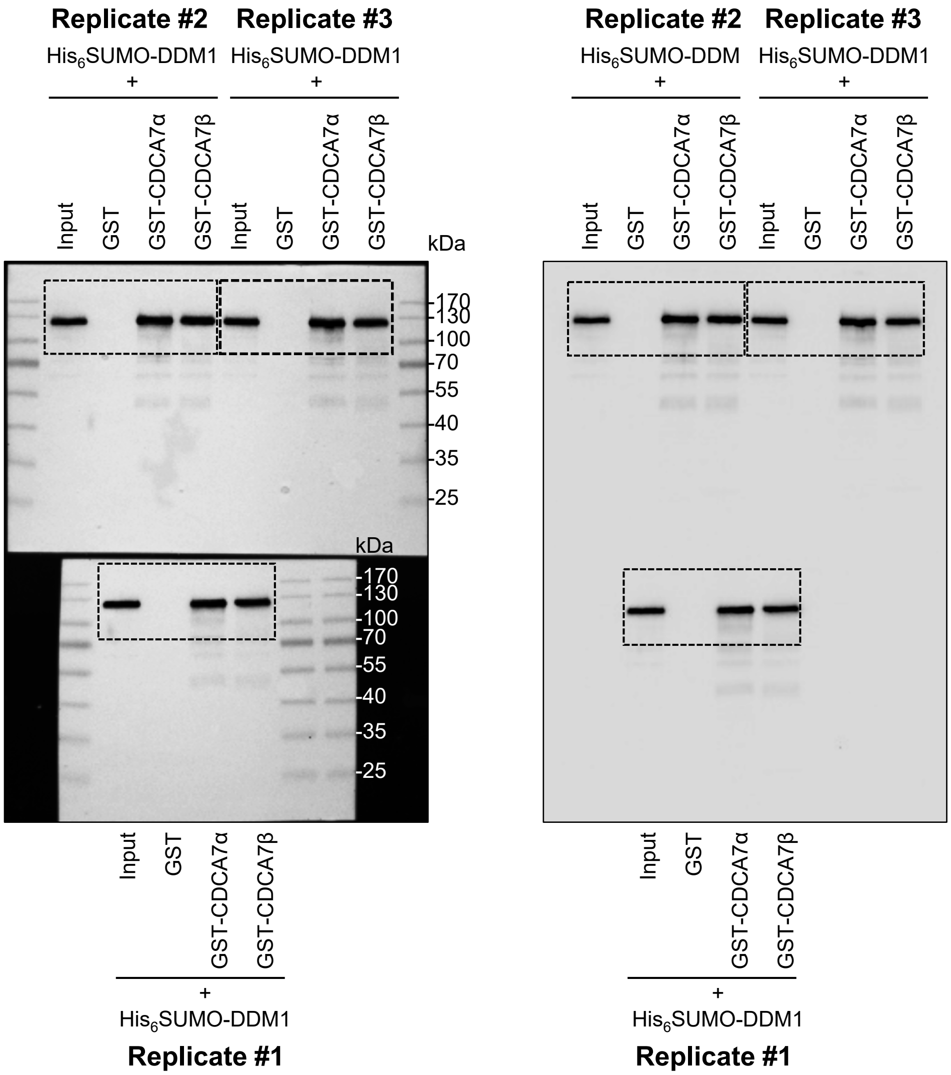

All WB: anti-DDM1

Source data for Figure 4e

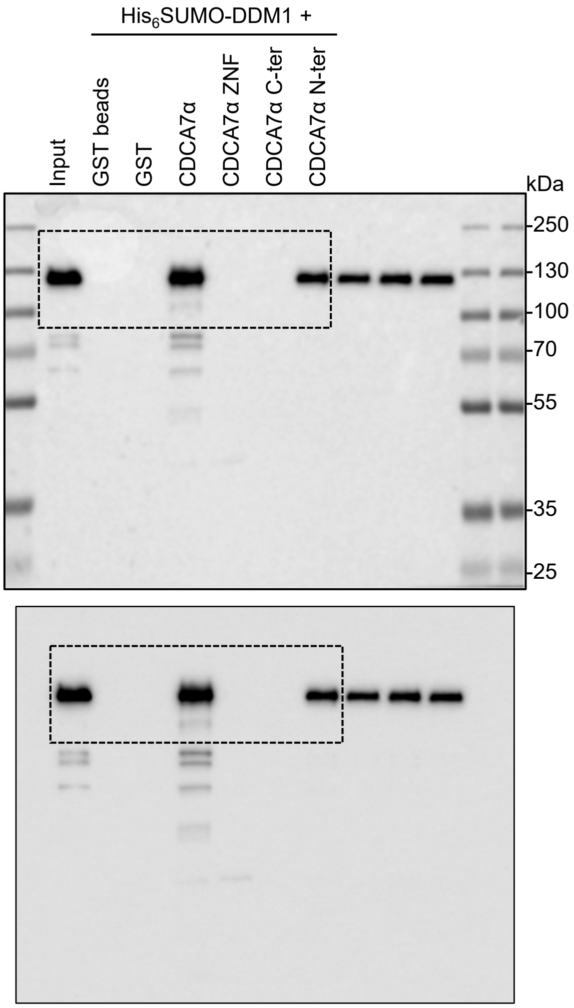

Replicate #1

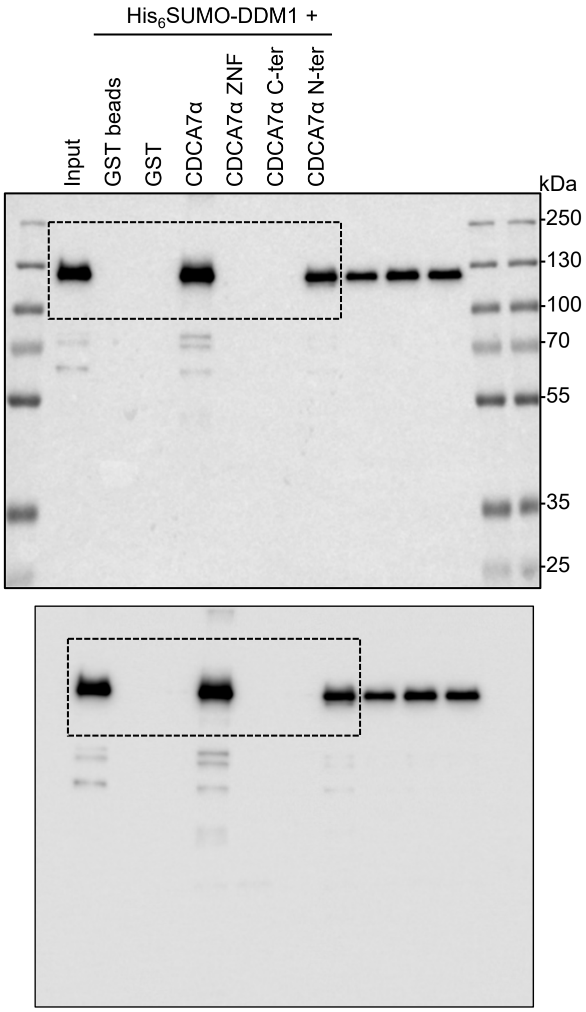

Replicate #2

All WB: anti-DDM1

Source data for Figure 4g and S6b

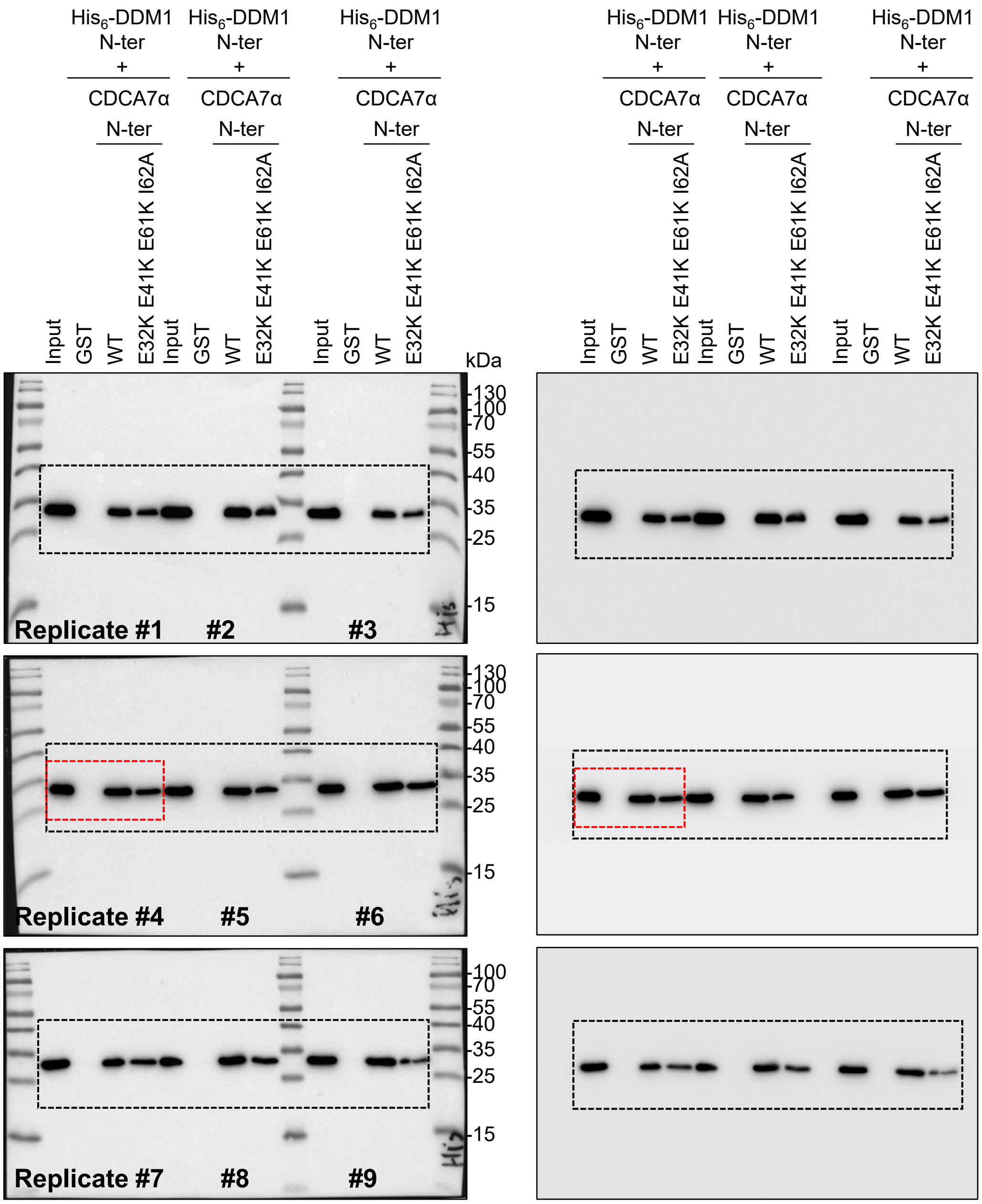

All WB: anti-His

Source data for Figure 4h and S6c

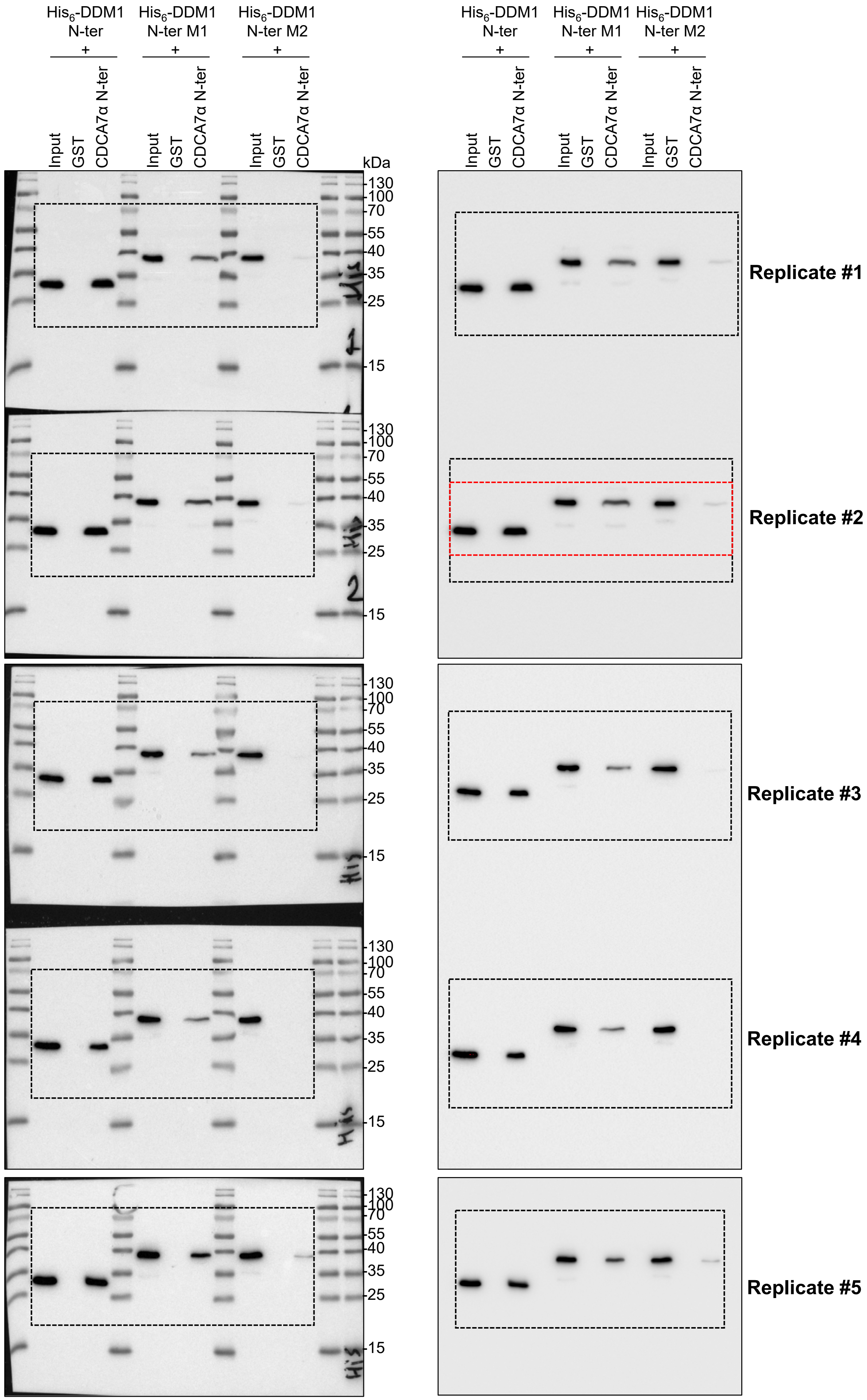

All WB: anti-His
