# Supplementary material for: Major alleles of CDCA7 shape CG methylation in Arabidopsis thaliana
Source: Nat Plants. 2025 Nov 7;11(12):2511–30. doi: 10.1038/s41477-025-02148-w (PMC12711577; doi:10.1038/s41477-025-02148-w)
Supplement: Supplementary file 11 — Unprocessed western blots. [file 41477_2025_2148_MOESM11_ESM.pdf]

# Source data for Extended Data Figure 8c

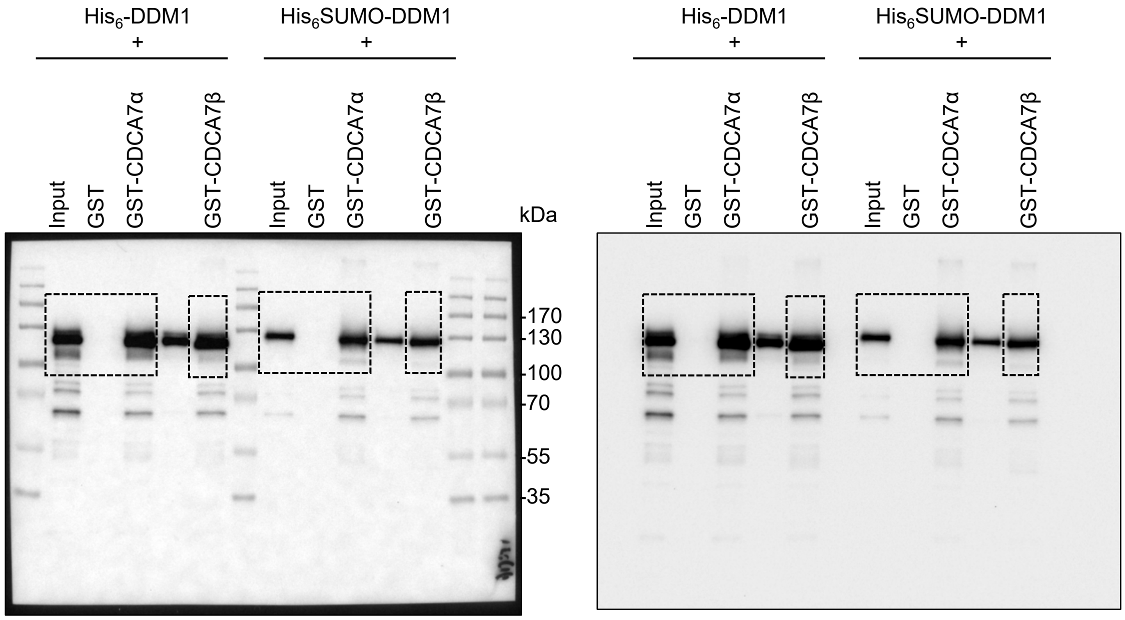


WB: anti-DDM1

## Source data for Extended data Figure 8f

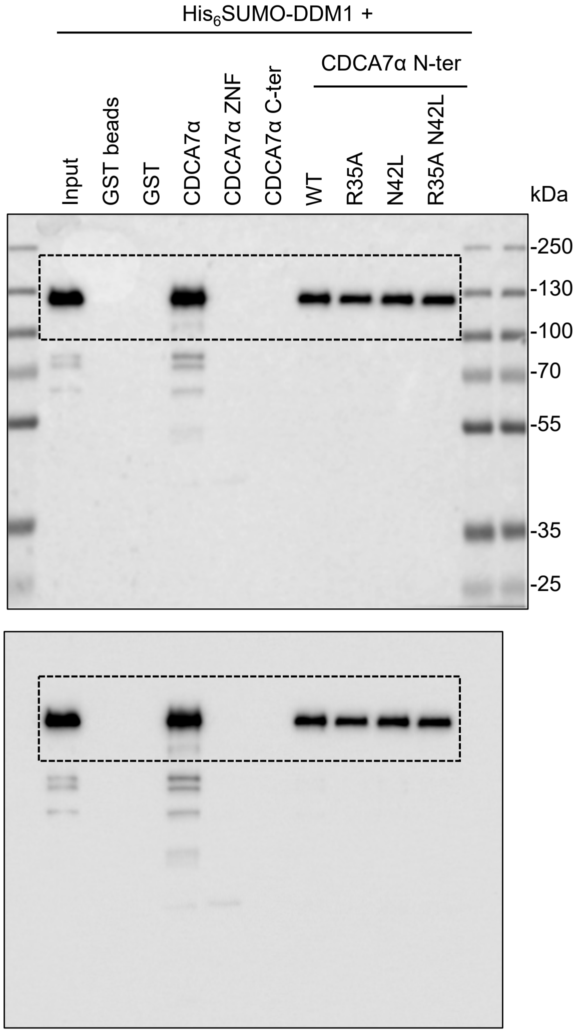


Replicate 1

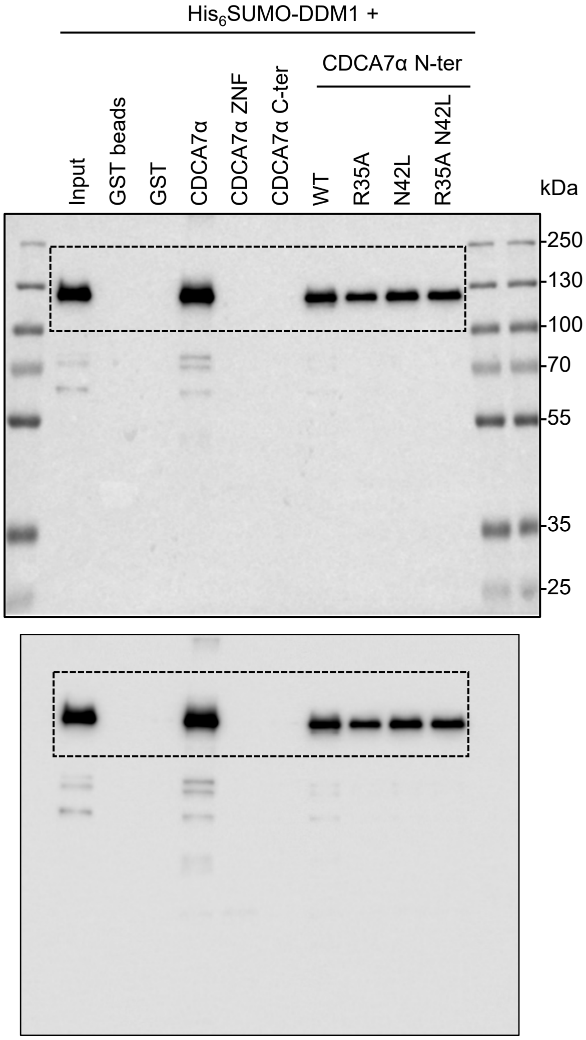


Replicate 2

All WB: anti-DDM1
