# Supplementary figures and images for: Major alleles of CDCA7 shape CG methylation in Arabidopsis thaliana
Source: Nat Plants. 2025 Nov 7;11(12):2511–30. doi: 10.1038/s41477-025-02148-w (PMC12711577; doi:10.1038/s41477-025-02148-w)

Source data for Extended data Figure 10b

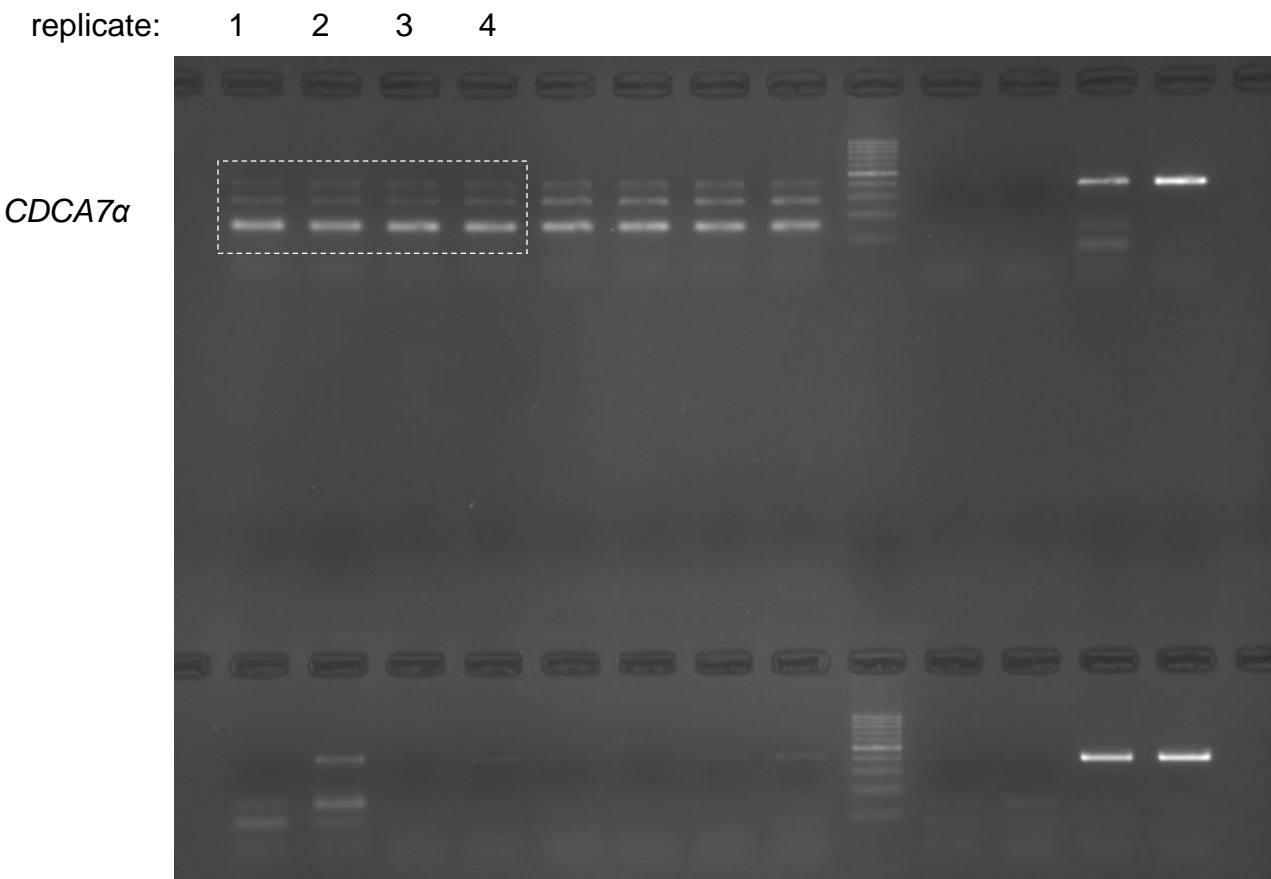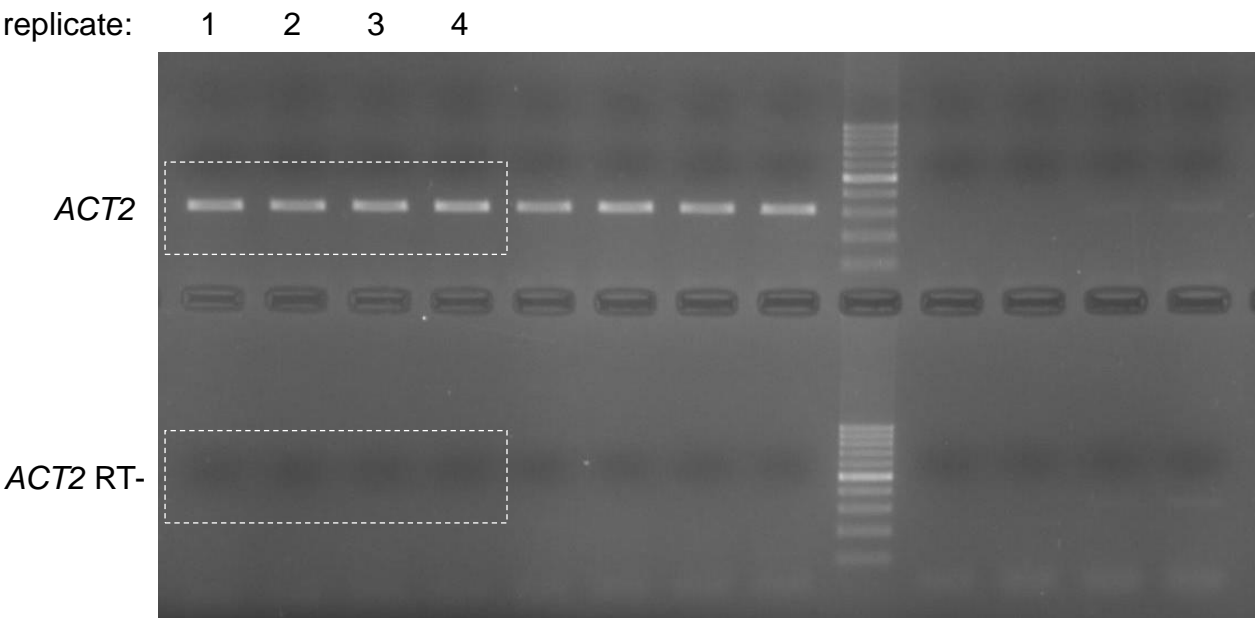

Supplement: Supplementary file 12 — Unprocessed gels. [file 41477_2025_2148_MOESM12_ESM.pdf]
